# Natural selection has driven the recurrent loss of an immunity gene that protects *Drosophila* against a major natural parasite

**DOI:** 10.1073/pnas.2211019120

**Published:** 2023-08-08

**Authors:** Ramesh Arunkumar, Shuyu Olivia Zhou, Jonathan P. Day, Sherifat Bakare, Simone Pitton, Yexin Zhang, Chi-Yun Hsing, Sinead O’Boyle, Juan Pascual-Gil, Belinda Clark, Rachael J. Chandler, Alexandre B. Leitão, Francis M. Jiggins

**Affiliations:** ^a^Department of Genetics, School of Biological Sciences, University of Cambridge, Downing Street, Cambridge CB2 3EH, United Kingdom; ^b^Department of Biochemical Sciences, School of Biosciences, University of Surrey, 388 Stag Hill, Guildford, GU2 7XH, United Kingdom; ^c^Biosciences Department, Università degli Studi di Milano, Via Celoria 26, Milano, MI 20133, Italy; ^d^School of Biomolecular and Biomedical Science, University College Dublin, Dublin D04 V1W8, Ireland; ^e^Facultad de Ciencias, Universidad Autónoma de Madrid, C. Francisco Tomás y Valiente 7, 28049 Madrid, Spain

**Keywords:** loss of function, *Leptopilina boulardi*, melanization, C-type lectin, *cis*-regulatory polymorphism

## Abstract

Polymorphisms in immunity genes can have large effects on susceptibility to infection. To understand the origins of this variation, we have investigated the genetic basis of resistance to the parasitoid wasp *Leptopilina boulardi* in *Drosophila melanogaster.* We found that increased expression of the gene *lectin-24A* after infection by parasitic wasps was associated with a faster cellular immune response and greatly increased rates of killing the parasite. *lectin-24A* encodes a protein that is strongly up-regulated in the fat body after infection and localizes to the surface of the parasite egg. In certain susceptible lines, a deletion upstream of the *lectin-24A* has largely abolished expression. Other mutations predicted to abolish the function of this gene have arisen recurrently in this gene, with multiple loss-of-expression alleles and premature stop codons segregating in natural populations. The frequency of these alleles varies greatly geographically, and in some southern African populations, natural selection has driven them near to fixation. We conclude that natural selection has favored the repeated loss of an important component of the immune system, suggesting that in some populations, a pleiotropic cost to *lectin-24A* expression outweighs the benefits of resistance.

## Significance

Genetic differences between individuals can have a large effect on susceptibility to infectious disease. We have identified a gene called *lectin-24A* that is important in the immune response that protects fruit flies against one of their main natural enemies—parasitic wasps. However, in nature, many flies carry mutated copies of this gene that are likely to be no longer functional. We found that the high frequency of these loss-of-function mutations can only be explained if they have a selective advantage in some populations. We conclude that this genetic variation in susceptibility is maintained because in some locations, susceptible flies are fitter than resistant flies.

Parasites can impose strong selection on host populations, driving resistance alleles up in frequency when infection is common (1, 2). Despite the advantages of resistance, genetic variability in susceptibility to infection is abundant in humans (3, 4), plants (5, 6), and insects (7–10). The polymorphisms underlying this variation may be transient as resistant alleles are spread through populations, or they can be maintained by temporal and spatial differences in selection pressures (11, 12) or negative frequency-dependent selection (13).

Variability in susceptibility can be maintained in populations when resistance trades-off with other fitness-related traits. These costs may occur in the absence of infection (14), due to the diversion of resources from growth and reproduction (15, 16), autoimmune damage (17), or when resistance to one pathogen increases susceptibility to a different pathogen (18). However, not all resistance alleles are costly (19), and over time, compensatory mutations that reduce or negate fitness costs may spread (20, 21). Alternatively, fitness costs could be avoided by reverting to susceptibility when the pathogen pressure is low (22).

Early demonstrations of the costs of evolving resistance came from *Drosophila* and parasitoid wasps, where populations selected for increased resistance had reduced competitive ability (23, 24) and lower feeding rates (25). Female parasitoid wasps lay their eggs inside the larvae of *Drosophila*, and if the host is unable to mount a successful immune response, the parasitoid larva feeds on the host tissue and eventually kills it. Flies can kill parasitoid wasps through a cellular immune response known as melanotic encapsulation, in which hemocytes (blood cells) are recruited to the parasitoid egg and surround it (26). The capsule is then melanized, killing the parasitoid egg. Despite parasitoids being common in nature, there is considerable variation within and between populations in susceptibility to the parasitoids *Asobara tabida* (Braconidae) (27) and *Leptopilina boulardi* (Figitidae) (28). Early work found polymorphisms in two regions of chromosome 2R that are involved in resisting parasitoid infection: one for resistance against *L. boulardi* and the other against *A. tabida* (29–31). However, a later study using populations that were artificially selected for resistance to the parasitoid *A. tabida* identified a 600-Kbp region on chromosome 2R that did not overlap with the previously identified loci (32). This could be the result of differences in the host genetic backgrounds or because intense artificial selection favors different loci to those favored by natural selection in the field (33). In the light of these observations and a lack of concrete evidence for selection acting on specific genes or alleles, we have attempted to identify the genetic basis of resistance against *L. boulardi* in a natural population of *Drosophila melanogaster*.

## Results

### Resistance to Parasitoid Infection Is Associated with a Faster Immune Response.

**Fig. 1.**
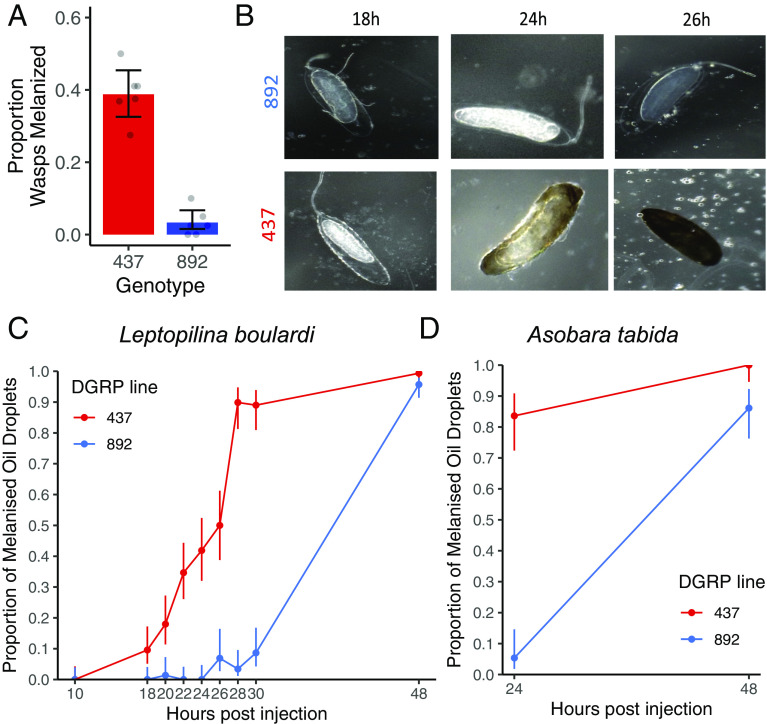
Genetic differences in the immune response to parasitoid infection. (*A*) The proportion of *L. boulardi* wasp embryos melanized in two inbred lines, DGRP-437 and DGRP-892. Each point is an independent replicate (six/line), bars are 95% CIs, and the number of larvae across all assays is above the bar. (*B*) Wasp embryo melanization at 18, 24, and 26 h after infection. (*C* and *D*) Larvae were injected with oil droplets containing homogenized *L. boulardi* (*C*, n_DGRP-437_ = 853, n_DGRP-892_ = 816) or *A. tabida* (*D*, n_DGRP-437_ = 128, n_DGRP-892_ = 128). Bars represent SEs.

We selected two inbred lines, DGRP-437 and DGRP-892, that had a marked difference in their ability to survive parasitoid infection to investigate the genetic basis of parasitoid resistance (Fig. 1*A*; binomial GLMM, *z* = −7.6, *P* < 0.001). The antiparasitoid defense response involves the wasp being surrounded by immune cells called hemocytes and melanized. In the resistant DGRP-437, the melanization phenotype becomes apparent at 24 h post-infection (hpi), and the wasp embryo is completely melanized at 26 hpi (Fig. 1*B*). In contrast, no melanization is seen in the susceptible DGRP-892 in the first 26 hpi (Fig. 1*B*).

This host immune response must be fast to succeed because once the wasp larva emerges from the egg chorion 24 to 48 hpi, it is mobile and better at escaping the cellular capsule (34). Parasitoids can suppress host immunity by injecting venoms along with the egg. To examine the speed of the immune response in the absence of this immune suppression, we triggered the immune response by injecting larvae with droplets of mineral oil containing homogenized parasitoid wasps (Fig. 1*C*). Both *Drosophila* lines had melanized the oil droplets by 48 h post-injection. However, the resistant line mounted this immune response faster—89.8% of the oil droplets were melanized at 28 h post-injection compared to 3.4% in the susceptible line (binomial GLM, logistic regression χ^2^ = 432.1, *df* = 1, *P* < 0.001). This difference is not specific to the parasitoid *L. boulardi,* as we obtained similar results when injecting oil droplets containing *A. tabida* homogenate (Fig. 1*D*; binomial GLM, genotype: logistic regression χ^2^ = 97.3, *df* = 1, *P* < 0.001; time post-injection: logistic regression χ^2^ = 111.5, *df* = 1, *P* < 0.001)]

### Resistance Results from Epistatic Interactions between Genes on Different Chromosomes.

**Fig. 2.**
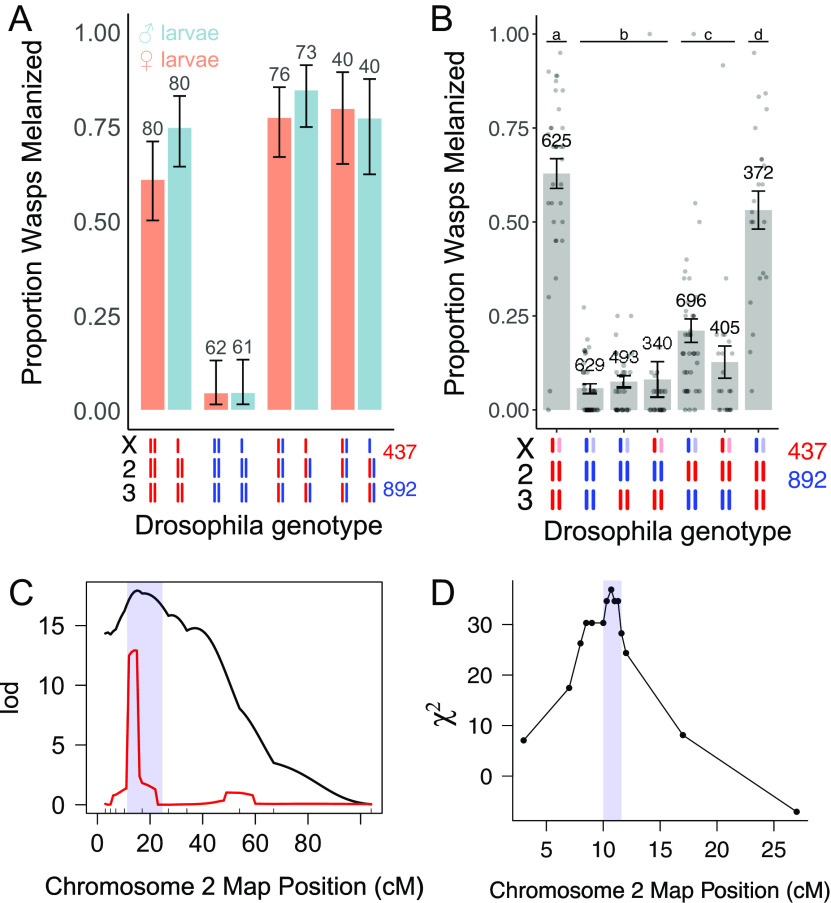
A single locus on chromosome II is associated with resistance to parasitoid wasp infection. (*A*) The mean proportion of wasps melanized in the F_1_ progeny of crosses between two *Drosophila* lines. Bars are 95% CIs. (*B*) The mean proportion of wasps melanized in lines with different chromosome combinations. Bars are SEs, and points are replicates of 20 larvae. Letters show significantly different groups (Tukey’s test, *P* < 0.05 between groups). Numbers above bars in (*A*) and (*B*) indicate the number of larvae that were assayed. (*C*) QTL on chromosome II associated with the melanization rate measured by dissecting larvae. The black line is interval mapping and the red line composite interval mapping. The blue region is the CI on the QTL location (1.5 LOD drop). *X* axis ticks are marker locations. (*D*) Fine-scale QTL in the region containing the gene. Only informative recombinants were genotyped. The χ^2^ statistic represents the deviation from the expected Mendelian frequency (0.5) of each marker among 152 adults that contained a capsule and tested positive for parasitoid wasp DNA. The shaded region indicates the 95% CI (χ^2^ drop of 4.6).

We next investigated the genetic basis of resistance. We chose to use classical genetic crosses rather than a genome-wide association study based on our experience of identifying genetic polymorphisms affecting susceptibility to infection. Specifically, this approach can identify genetic variants in the presence of epistasis, allelic heterogeneity, and low allele frequencies. This can be challenging using genome-wide association studies in *Drosophila,* as the available resources mean such analyses frequently use under 200 fly lines. When we crossed the resistant and susceptible lines, the F_1_ progeny were highly resistant, indicating that resistance was dominant (Fig. 2*A*). By swapping whether the resistant parent was the mother or father, we generated male offspring that only differed in their X chromosome (Fig. 2*A*). Neither the sex of progeny (likelihood ratio test, χ^2^ = 3.46, *P* = 0.06) nor the X genotype (Tukey’s HSD, *z* = 0.45, *P* = 0.65) had a significant impact on susceptibility (Fig. 2*A*). These results demonstrate that resistance is an autosomal dominant trait.

To identify the chromosomes affecting susceptibility, we generated lines carrying varying combinations of the X, II, and III chromosomes (Fig. 2*B* and *SI Appendix*, Fig. S1). When comparing lines that differ only in their second chromosome, having chromosome II from the resistant parent always resulted in greater melanization (Fig. 2*B*). When paired with a third chromosome from the resistant parent, swapping chromosome II could convert a fully resistant line into a fully susceptible line (Fig. 2*B*). However, when paired with a third chromosome from the susceptible parent, chromosome II only had a small effect. This is reflected in a statistical interaction between the two chromosomes (GLM with logit link, Wald test: *χ^2^*= 24.7, *P* < 0.001), indicating that there is a multiplicative epistatic interaction between the second and third chromosomes.

### A Major Effect Locus on Chromosome II Affects Resistance.

We used quantitative trait locus (QTL) mapping to locate the region on chromosome II affecting susceptibility to infection. We crossed fly lines that differed only in the second chromosome (X^892^; II^892^; III^437^ and X^892^; II^437^; III^437^) and then backcrossed the F_1_ progeny to the susceptible parent. The resulting larvae were parasitized, and we genotyped 386 individual larvae using 10 molecular markers spanning chromosome II. We identified a single region where the chromosome II genotype was associated with differences in susceptibility (Fig. 2*C*, black line). Based on a 1.5 logarithm of the odds (LOD) drop, the QTL encompassed 11 to 25 cM (Fig. 2*C*, blue box). Composite interval mapping, which searches for additional QTLs while accounting for the main peak, indicated that there is a single locus on chromosome II affecting susceptibility (Fig. 2*C*, red line).

As this QTL contained many genes, we conducted a second round of genetic mapping using only flies that were recombinant within the QTL. We repeated the genetic cross, parasitized the backcrossed larvae, and selected adults that contained visible capsules in their body and had therefore likely survived infection (susceptible flies were killed). We genotyped these individuals using molecular markers flanking the QTL (3 to 27 cM) to identify recombinants. Out of 1,486 adults, 298 had a recombination breakpoint between 3 cM and 27 cM—a recombination fraction of 0.20. We genotyped 12 molecular markers within this region for 152 individuals where we could amplify wasp DNA to confirm that they had been infected. As we only genotyped resistant flies, we tested whether marker allele frequencies departed from the 50:50 Mendelian expectation. Sixty-one out of 348 uninfected flies had a breakpoint between 3 cM and 27 cM, which is only marginally lower than expected suggesting little segregation distortion. A χ^2^ drop to identify informative markers was selected by simulating 1,000 datasets based on the observed risk ratio estimated from nonrecombinant flies and the observed recombination fraction. The χ^2^ drop defined a region that included the gene in 95% of simulations. Using this approach, we identified a single QTL at 10.3 cM on the left arm of chromosome II (Fig. 2*D*). By simulating 1,000 replicate datasets, we estimated that the 95% CI on the location of the QTL contained 84 protein-coding genes and 23 long noncoding RNAs (10.0 to 11.6 cM, genome v6: 3.43-4.03Mbp, *SI Appendix*, Table S1). Among the 1,188 nonrecombinant flies, 906 carried the resistant allele and 282 the susceptible allele, indicating a risk ratio of 3.21 (homozygous susceptible versus heterozygotes).

### A C-Type Lectin Underlies Resistance.

**Fig. 3.**
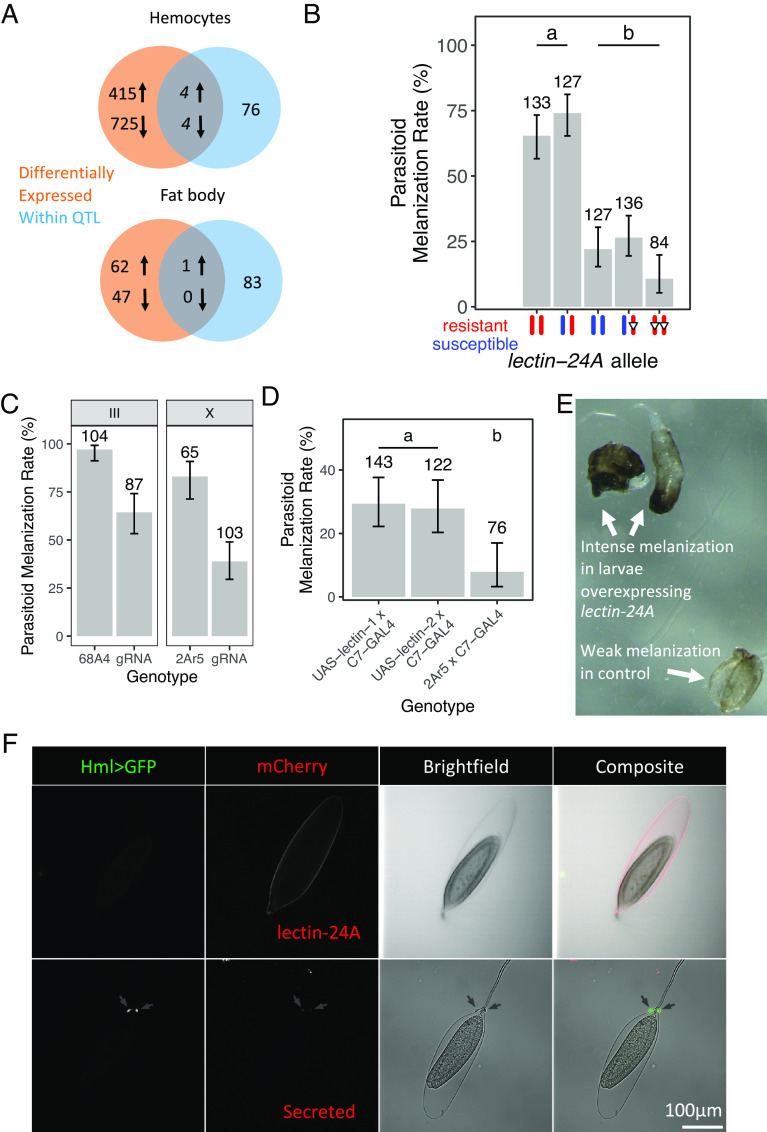
*lectin-24A* is required for parasitoid resistance. (*A*) Protein-coding genes within the QTL and differentially expressed after immune induction (log_2_ fold change > 2). The gene in the intersection of the fat body Venn diagram is *lectin-24A.* (*B*) Mean parasitoid melanization rate in lines with resistant, susceptible, and mutated (open inverted triangles, *Δ129*) *lectin-24A*. Error bars represent 95% CIs. Tukey’s test, *P* < 0.05 between groups. (*C*) Mean parasitoid melanization rates in larvae expressing *Act-Cas9* and guide RNAs targeting *lectin-24A* compared to control larvae. Guide RNAs on either the X or the III chromosome were used, and 2Ar5 and 68A4 are the lines in which the construct was microinjected. Each of these lines was crossed to Act-Cas9; II^437^; III^437^, and the F_1_ heterozygotes were assayed. Error bars represent 95% CIs. A Fisher’s exact test was used to identify significant differences for each pair of comparisons. (*D*) Mean parasitoid melanization rates in larvae overexpressing *lectin-24A* under the *C7-GAL4* driver in the larval fat body compared to control larvae. UAS-lectin-1 and UAS-lectin-2 are independent insertions of a construct expressing guide RNAs targeting *lectin-24A*, and 2Ar5 is the original line in which the construct is microinjected into. Error bars represent 95% CIs. Tukey’s test, *P* < 0.05 between groups. Numbers above bars in (*B*–*D*) indicate the number of larvae that were assayed. (*E*) Representative image of partially melanized wasp larva and melanized wasp eggs in *Drosophila* larvae overexpressing *lectin-24A* and control larvae. (*F*) Confocal microscopy image of wasp eggs dissected 2 to 12 h post-infection. The presence of hemocytes (arrows) was checked with HmlΔ-GAL4 UAS-GFP and confirmed in the brightfield (BF) channel. lectin-24A-mCherry can be observed on the egg chorion in regions without hemocytes (top row). The same is not observed in secreted-mCherry alone (bottom row). The scale bar represents 100 μm.

Parasitoid wasp infection induces a large transcriptional response in the two main immune tissues of *Drosophila—*the fat body and hemocytes. Using our open access RNA sequencing data (35), we searched for genes within the QTL that were up-regulated after antiparasitoid immune induction in these tissues (log_2_ fold change > 2). We found that eight genes were differentially expressed in hemocytes and one gene*, lectin-24A*, in the fat body (Fig. 3*A*). When we measured the expression of these nine genes in the lines we are studying, only *lectin-24A* showed differential induction following parasitic wasp infection (*SI Appendix*, Fig. S2). *lectin-24A* has previously been found to be massively up-regulated following parasitoid wasp infection (36–38). Furthermore, the marker most strongly associated with the melanization rate in the fine-scale QTL analysis is located within *lectin-24A*. As lectins are important receptors in innate immune systems, we focused on this gene.

To test whether *lectin-24A* is necessary for resistance to parasitoid wasps, we created a germline mutation in the resistant *lectin-24A* allele using CRISPR-Cas9 in the resistant X^892^; II^437^; III^437^ flies. We created a 4-bp insertion that introduced a premature stop codon 129 bp downstream of the start codon, which we named *lectin-24A^Δ129^*. The change in reading frame introduces a premature stop codon and abolishes the carbohydrate-binding domain of the protein. This mutation made flies susceptible to infection (Fig. 3*B*; Tukey’s HSD test: *P* < 0.001).

To confirm this result, we generated somatic *lectin-24A* mutants in the F_1_ progeny of a cross between flies that ubiquitously express Cas9 and fly lines that we created to express guide RNAs targeting the *lectin-24A* gene (*SI Appendix*, Fig. S3*A*). This efficiently generated somatic mutants (*SI Appendix*, Fig. S3*B*), and the mutant larvae showed significantly reduced melanization rates when parasitized compared to larvae that express Cas9 but not the gRNAs (Fig. 3*C*; Fisher’s exact test: *P* < 0.001). We repeated this experiment using an independently generated fly line that carried the guide RNA on a different chromosome and obtained the same result (Fig. 3*C*; Fisher’s exact test: *P* < 0.001).

We next overexpressed *lectin-24A* in the larval fat body. We generated flies carrying a UAS-driven Flag-tagged lectin-24A construct with the DGRP-437 coding sequence, under the control of the *C7-GAL4*, which drives expression in the larval fat body. The overexpression of *lectin-24A* increased the melanization rate in *Drosophila* larvae against parasitic wasp infection (Fig. 3*D*; Tukey’s HSD test: *P* < 0.005 between groups). In larvae overexpressing *lectin-24A,* we frequently observed partially melanized wasp larvae (Fig. 3*E*). There was also a striking and consistent increase in the intensity of melanization between the larvae overexpressing *lectin-24A* and the controls (Fig. 3*E*).

lectin-24A is a C-type lectin, a family of proteins which frequently act as pattern recognition receptors in the innate immune system due to their specificity in binding ligands (39). To investigate the role of *lectin-24A* in the immune response, we created transgenic flies that expressed lectin-24A fused to mCherry fluorescent protein under the control of the gene’s native promoter. When larvae from these lines were infected by a parasitoid, the protein localized to the surface of the wasp egg at an early time point (Fig. 3*F*). To understand at what point in the immune response this occurred, we also visualized hemocytes using both brightfield microscopy and by expressing GFP in plasmatocytes (at this time point lamellocytes have not yet differentiated). This revealed that lectin-24A is found on the parasitoid egg before hemocytes attached to the egg (Fig. 3*F*). This is consistent with this molecule being an opsonin involved in the initial recognition of the parasite, guiding the subsequent cellular immune response.

C-type lectins are named due to their ability to bind to specific carbohydrates in a calcium-dependent manner (39). By aligning the peptide sequence of the lectin-24A carbohydrate recognition domain with other members of the protein family, we found that residues required for the interaction with the calcium ions have been lost, suggesting that it is not involved in calcium-dependent carbohydrate binding (*SI Appendix*, Fig. S4*A*). This is reminiscent of another well-characterized group of C-type lectins that have lost the calcium-binding ability—natural killer cell receptors—which bind ligands including proteins (40). Alongside conserved cysteines involved in forming the Ca^2+^ binding site, lectin-24A contains an additional cysteine within the carbohydrate domain and four cysteines elsewhere in the protein (*SI Appendix*, Fig. S4*A*), suggesting that it may form multimers. By expressing affinity-tagged lectin-24A in *Drosophila* cells, we confirmed the protein forms tetramers (*SI Appendix*, Fig. S4*B*).

### A *Cis-*Regulatory Polymorphism in *lectin-24A* Is Associated with Resistance.

**Fig. 4.**
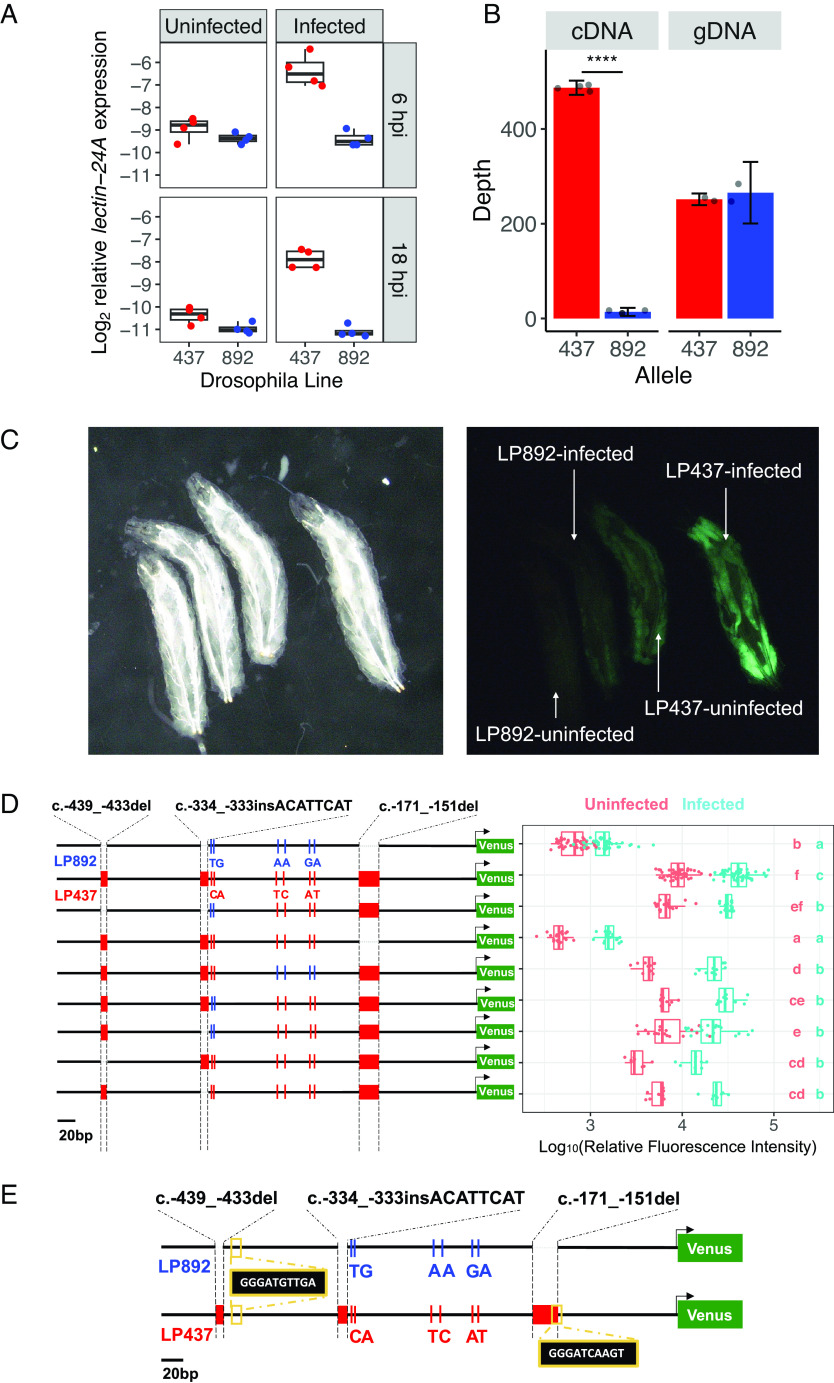
*Cis*-regulatory polymorphisms in *lectin-24A.* (*A*) Expression of *lectin-24A* 6 and 18 h post-infection (hpi) with parasitoid wasp. (*B*) Allele-specific expression of *lectin-24A* in heterozygous flies. Read counts (depth) of the resistant (DGRP-437) and susceptible (DGRP-892) allele for complementary DNA (cDNA) and genomic DNA (gDNA). Asterisks indicate a Welch *t* test, *P* < 0.0001. (*C*) Expression of Venus driven by the sequence upstream of the susceptible (LP892) or resistant *lectin-24A* (LP437) imaged 24 hpi under brightfield (*Left*) or GFP filter (*Right*). (*D*) Reporter constructs with different combinations of variants upstream of *lectin-24A* in the susceptible (DGRP-892, blue) and resistant (DGRP-437, red) lines and expression of Venus. Each point represents a sample of 15 larvae. Letters are Tukey’s test, *P* < 0.05 between groups, *P* > 0.05 within groups. (*E*) Putative binding site of the NF-κB transcription factors in the lectin-24A promoter regions of DGRP-437 and DGRP-892.

We used qPCR to examine whether the resistant and susceptible copies of *lectin-24A* differed in their expression (Fig. 4*A*). In uninfected larvae, the resistant *lectin-24A* is expressed at a higher level than the susceptible *lectin-24A*. After infection, there was a ~2.5-fold upregulation of the resistant *lectin-24A*, but the susceptible copy was not induced (6 and 18 hpi, Fig. 4*A* and *SI Appendix*, Fig. S2; ANOVA, effect of *Drosophila* line: *F* = 41.69, *df* = 1, *P* < 0.001).

To determine whether *lectin-24A* expression is controlled in *cis* or *trans*, we crossed the two lines, infected them, and Illumina-sequenced the *lectin-24A* transcript in the heterozygous F_1_ progeny. The sequence reads were assigned to the resistant and susceptible *lectin-24A* using SNPs that differ between the two lines, allowing us to measure their relative expression. In these heterozygous flies, we found that the expression of the resistant *lectin-24A* was 34 times greater than the susceptible *lectin-24A* (Fig. 4*B*; Welch *t* test: *t* = 135.6, *df* = 4.7737, *P* < 0.001). As the two alleles of *lectin-24A* are present in the same cells, they share the same *trans*-regulatory environment, these differences in expression are controlled in *cis.*

Many *cis*-regulatory elements are found a short distance upstream of the gene they control. The *lectin-24A-mCherry* transgene described above included 489 bp of sequence upstream of the start codon of the resistant *lectin-24A*, and this is strongly induced after infection (*SI Appendix*, Fig. S5). However, when the equivalent transgene was made from the susceptible *lectin-24A,* there was no detectable expression (*SI Appendix*, Fig. S5). To confirm this result, we cloned these regulatory sequences in front of GFP to create fluorescent reporters that were inserted into the *Drosophila* genome. Recapitulating the results from the *lectin-24A-mCherry* construct, the sequence upstream of resistant *lectin-24A* drove strong reporter expression in the fat body, but the equivalent sequence upstream of the susceptible *lectin-24A* did not (Fig. 4*C*). To accurately quantify expression, we measured fluorescence in proteins extracted from larvae. Confirming the microscopy results (Fig. 4*C*), we observed a ~30-fold difference in fluorescence between the reporter lines carrying the regulatory sequence of the resistant and susceptible *lectin-24A* (Fig. 4*D*, top two constructs). While *lectin-24A* was never up-regulated after infection in DGRP892 (Fig. 4*A*), the DGRP-892 promoter can drive GFP induction in our transgenic flies, albeit from a low level (Fig. 4*C*). The reason for this is unknown, but it suggests that the behavior of this regulatory sequence depends on its genomic location. Together, this demonstrates that this region contains a *cis-*regulatory polymorphism that differs between the resistant and susceptible *lectin-24A*.

Comparing the resistant and susceptible *lectin-24A*, the *cis*-regulatory sequence used in the reporter constructs differed by three insertion-deletion polymorphisms (indels) and six single nucleotide polymorphisms (SNPs; Fig. 4*D*, top two rows). To identify which of these causes the differences in expression, we created seven more transgenic fly lines, each carrying a reporter construct that had a different combination of alleles at these sites (Fig. 4*D*). When we introduced a 21 bp indel (c.-171_-151del) found upstream of the susceptible *lectin-24A* into the resistant *lectin-24A* reporter, it greatly reduced the expression levels (Fig. 4*D*). In contrast, swapping alleles of the other polymorphic sites only resulted in minor but statistically significant changes in expression (~threefold). Therefore, we conclude that the 21 bp indel (c.-171_-151del) is primarily responsible for the differential *lectin-24A* expression in the resistant and susceptible lines.

To understand why the 21 bp deletion reduces *lectin-24A* expression, we predicted binding sites of *Drosophila* immunity-related transcription factors (41–43). A putative binding site of the NF-κB transcription factors Dif and dorsal is lost with the 21 bp deletion (c.-171_-151del) (Fig. 4*E*). These transcription factors are controlled by the Toll pathway—a major immune signaling pathway—so the loss of this binding site might cause the loss of *lectin-24A* expression.

### In a Population, *lectin-24A* Expression Is Associated with Susceptibility.

**Fig. 5.**
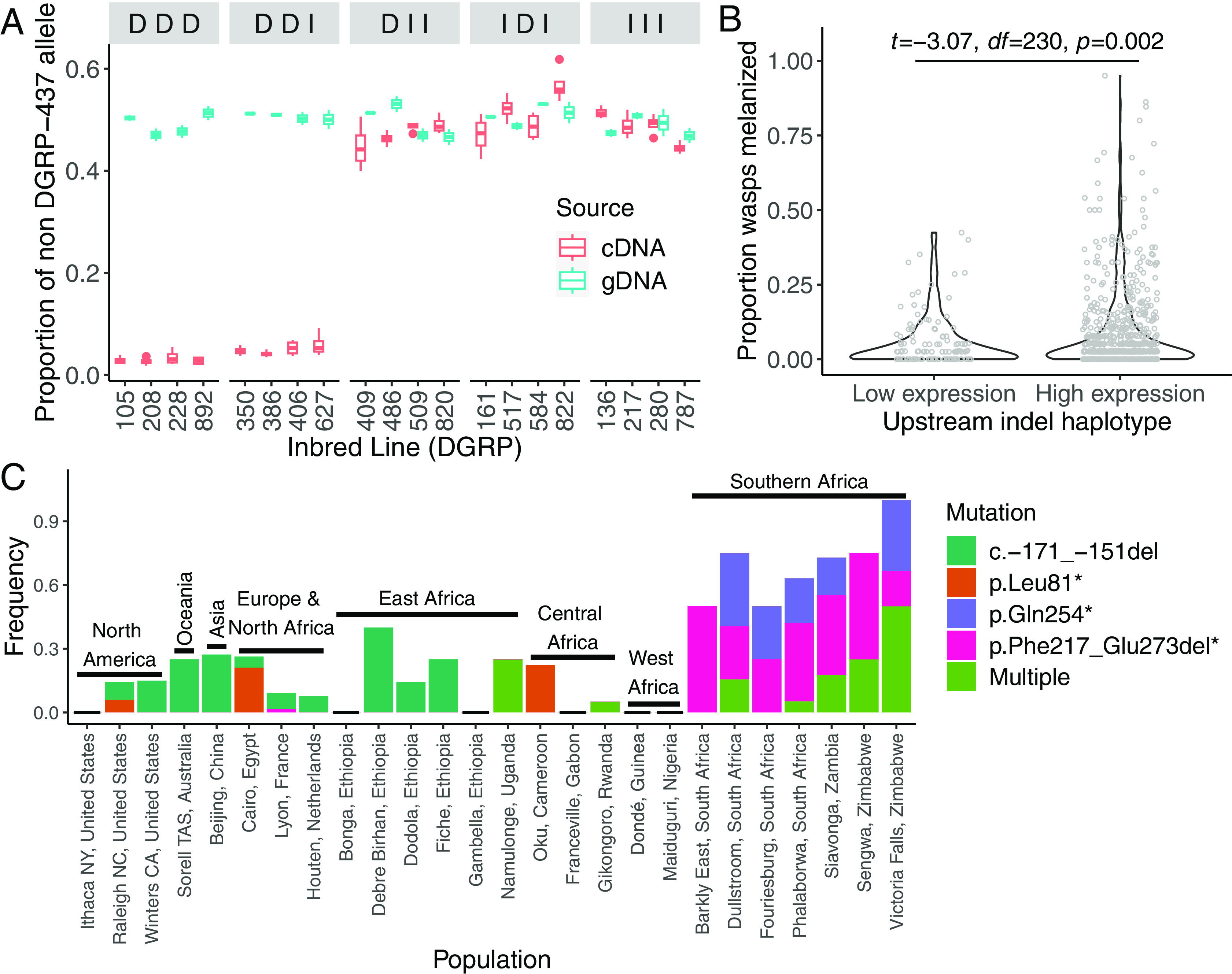
The frequency of predicted loss-of-function alleles of *lectin-24A* in natural populations. (*A*) Twenty-four hours post-infection, *lectin-24A* complementary DNA (cDNA) and genomic DNA (gDNA) was sequenced in the F_1_ progeny of a cross between DGRP-437 and 20 different inbred DGRP lines. The lines are grouped by their indel haplotype, which is ordered as c.-439_-433del, c.-334_-333insACATTCAT, and the 21 bp indel (c.-171_-151del), and the deletion “D” or insertion “I” state for each indel is depicted. We estimated the frequency of reads from the test line as opposed to DGRP-437. We had two technical and two biological replicates per cross. Data for DGRP-892 are also presented in Fig. 3. (*B*) Melanization rates in adult flies from 145 DGRP lines where the three upstream *lectin-24A* indels were characterized. Lines are grouped by whether they have the upstream indel haplotypes capable of expressing *lectin-24A* at a high level. The 2 to 10 replicate measurements for melanization rates per DGRP line are shown as individual points. A total of 123 lines have the *lectin-24A* expressing haplotypes (DII, IDI, III), and 22 lines have the haplotypes that do not express *lectin-24A* (DDD, DDI). A Welch two-sample *t* test was used to identify significant differences between the line means of the two groups. (*C*) The frequency of variants that abolish expression (the 21 bp indel as known as c.-171_-151del) or introduce premature stop codons (other variants). Multiple refers to the co-occurrence of any two loss-of-function variants. Analysis used 672 genome sequences genotyped for >50% of the *lectin-24A* gene.

We next investigated genetic variation in *lectin-24A* expression at the population level. We first sequenced a 557-bp region upstream of the gene in the DGRP panel of inbred lines from North America (44, 45) (*SI Appendix*, Table S2) and selected 20 lines with different haplotypes at the three indels shown in Fig. 4*D*. We crossed these to our resistant line (DGRP-437) and Illumina-sequenced the *lectin-24A* transcript in the F_1_ progeny to look for evidence of allele-specific expression. This assay produced consistent results across replicates (*SI Appendix*, Fig. S6 *A* and *B*), and when we sequenced genomic DNA, the frequency of the two alleles was close to 0.5, indicating that there is no technical bias toward one allele (Fig. 5*A*).

As expected, we found that the four lines carrying the 21 bp deletion (c.-171_-151del) all had very low expression (Fig. 5*A*, haplotype DDD). However, four lines that lack the deletion at this site also had strongly reduced expression (Fig. 5*A*, haplotype DDI). While expression in these lines was low, it was nonetheless 1.7 times higher than lines with the 21 bp deletion (quasibinomial GLM, DDD versus DDI: *t* = 4.319, *P* < 0.001). To confirm these results, we also measured *lectin-24A* expression in a sample of inbred lines by qPCR. These results supported our conclusion that lines with the 21 bp deletion had the lowest expression, but the expression was also reduced in lines with the DDI haplotype (*SI Appendix*, Fig. S6*C*). Twenty-five of the 130 fully genotyped lines carried one of the low expression haplotypes (DDD or DDI). After removing lines with the 21 bp deletion, alternate alleles at four sites in the 1,000 bp region upstream of the *lectin-24A* start codon (3718317, 3718354, 3718388, and 3718452) showed complete association with the loss of capacity to induce *lectin-24A* expression and are therefore candidate *cis*-regulatory polymorphisms (*SI Appendix*, Table S3).

To examine *lectin-24A* expression in a larger sample at a different life stage, we reanalyzed published RNA-sequencing data from adult DGRP flies (46). We found that the haplotypes that were associated with low expression in infected larvae also had lower expression in uninfected adults (*SI Appendix*, Fig. S7). This effect in uninfected flies is consistent with the results in uninfected larvae, where alleles of both the gene and reporter construct that carry the 21 bp deletion have reduced expression (Fig. 4 *A* and *D*).

To test whether the *cis*-regulatory polymorphisms in *lectin-24A* are associated with parasitoid resistance at the population level, we estimated the melanization ability of 194 lines in the DGRP panel. In total, we examined whether 39,696 flies across 1,060 replicate vials melanized wasp larvae. The lines varied greatly in their melanization rate, with most being very susceptible to infection. The DGRP lines with the low expression haplotypes (DDD or DDI) had significantly lower melanization rates compared to lines capable of expressing *lectin-24A* (Fig. 5*B*). After accounting for the expression haplotype in an ANOVA, variants in the coding sequence did not have a significant effect on melanization rates (*SI Appendix*, Table S4). All the lines with the highest melanization rates had high-expression haplotypes. However, most lines with high-expression haplotypes were susceptible to infection, indicating that *lectin-24A* expression is not sufficient for resistance. This is consistent with our finding above that this gene only provides strong resistance in specific genetic backgrounds. The finding that high expression haplotypes are associated with parasitoid resistance provides confirmation that this gene underlies resistance.

### Loss-of-Function Alleles Have Arisen Repeatedly in Natural Populations.

We have evidence for two alleles that cause the loss of *lectin-24A* expression—the 21 bp deletion and the DDI haplotype (Fig. 5*A*). We next examined whether loss-of-function variants are segregating in the gene’s protein-coding sequence. It has previously been reported that there are alleles of *lectin-24A* containing premature stop codons in natural populations (47). As many more genomes have been sequenced since that analysis, we searched 1,039 published genomes from flies collected globally for variants that are likely to result in null alleles of *lectin-24A* (48). We identified a 165-bp deletion in the protein-coding sequence that resulted in a shift in the reading frame and a premature stop codon (p.Phe217_Glu273del*) and three point mutations that introduced premature stop codons either within or before the lectin-24A carbohydrate recognition domain. Lines containing these premature stop codons were able to up-regulate *lectin-24A* following parasitoid wasp infection (*SI Appendix*, Fig. S8), suggesting that these variants cause the loss of gene function independently of the loss-of-expression mutations.

To understand the geographical distribution of putative loss-of-function alleles in *lectin-24A*, we examined their frequency in 26 populations. Southern African populations have the highest frequency of loss-of-function alleles, with over half of alleles carrying a premature stop codon, and many alleles carrying multiple loss-of-function mutations (Fig. 5*C*). Outside of southern Africa, premature stop codons are rare, but the 21-bp deletion (c.-171_-151del) that abolished expression is widespread, reaching frequencies over 30% in some locations. These numbers underestimate the true frequency of loss-of-function alleles as we cannot identify the loss-of-expression variant on the DDI haplotype from sequence alone.

### Natural Selection Has Driven the Loss of *lectin-24A*.

Given the importance of *lectin-24A* in defending flies against parasitoid wasps, the finding that likely nonfunctional alleles are common in nature is unexpected. We therefore explored the evolution of the gene in more detail. First, we examined whether predicted nonfunctional alleles are the ancestral or derived states by aligning the *lectin-24A* gene region with the homologous region from three closely related species—*Drosophila mauritiana*, *Drosophila simulans,* and *Drosophila sechellia*. All three species contain the 21 bp sequence (AAATAAGGCTATCTGGGATCA; c.-171_-151del; *SI Appendix*, Fig. S9) that is required for the gene to be induced after infection and do not contain any premature stop codons in the coding sequence. Therefore, in all cases, the loss-of-function allele is the derived state.

**Fig. 6.**
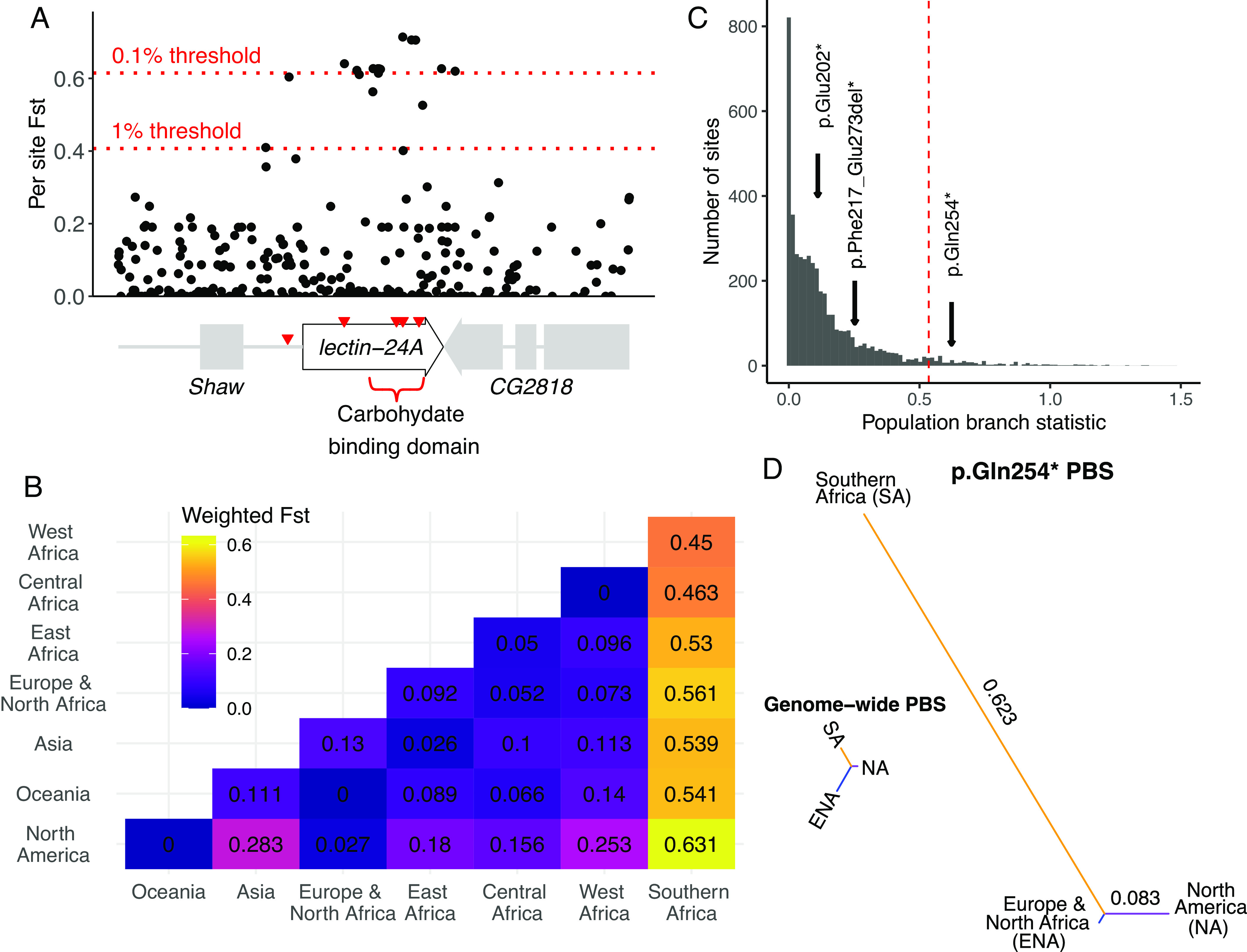
Evidence of local adaptation in *lectin-24A.* (*A*) Per site Weir and Cockerham *F_ST_* (dm5; 2L:3715513–3719050) across 26 global fly populations. *F_ST_* for the top 1% and 0.1% of 23,635 variants in short introns is indicated by dotted red lines. Red inverted triangles indicate loss-of-function variants. (*B*) *F_ST_* for *lectin-24A* protein coding sequence from pairwise comparisons between seven geographic regions. (*C*) Population branch statistic (PBS) for 4,433 variants in short introns and three *lectin-24A* premature stop codons for the three largest geographic regions. The premature stop codons are indicated with arrows. The red dotted line is the 95th percentile. (*D*) PBS trees for the polymorphic stop codon p.Gln254* and a genome-wide tree based on 4,433 variants in short introns. Both trees are on the same scale.

The variable frequency of loss-of-function alleles in nature (Fig. 5*C*) suggests that these alleles may be favored by natural selection in some populations but not others. In line with previous analyses of a smaller dataset (47), multiple SNPs in *lectin-24A* had very high levels of genetic differentiation in the 1,039 published genomes (48), with several SNPs being below the 0.1% percentile of a null distribution generated using 23,635 variants that occur in neutrally evolving short introns (Fig. 6*A*). Pairwise comparisons between geographical regions showed that this pattern was driven by the southern African populations being highly differentiated from other regions (Fig. 6*B*). Across the gene, there are numerous variants that are near fixation across southern Africa but are rare elsewhere in the world (*SI Appendix*, Fig. S10) due to a divergent haplotype that is common only in southern Africa (*SI Appendix*, Fig. S11). Two of the three premature stop codons and the coding sequence deletion (p.Phe217_Glu273del*) were at their highest frequency (16.7 to 56.9%) in southern Africa. The other premature stop codon (p.Leu81*) was not found in southern Africa but was segregating at 4 to 5% in North America, Europe, and North Africa and Central Africa.

As the loss of *lectin-24A* has a large effect on susceptibility to infection, we tested whether natural selection has driven the premature stop codons to a high frequency in southern Africa using the population branch statistic (PBS) (Fig. 6*C*). This used pairwise *F_ST_* estimates between the three geographical regions with large numbers of published genomes to generate a tree, with longer branches indicative of larger changes in allele frequency along that branch (49). To generate an empirical null distribution, we calculated the PBS for 4,433 variants that short introns (which are regarded as putatively neutral), had a minor allele frequency greater than 5%, and were typed >40% of samples in each region. We found that p.Gln254* had an extremely high PBS in southern Africa when compared to the genome-wide PBS, indicating that this variant is under positive selection in that region (Fig. 6*D*). We also performed a selection test using Ohana (50) which controls for admixture and historical population structure. We first used genome-wide data to generate a covariance matrix of allele frequencies between ancestry components, and then scanned for local distortions due to positive selection. We used 4,433 variants in short introns to generate an empirical null distribution. We found a strong signature of positive selection on p.Gln254* along the Southern African branch (*SI Appendix*, Fig. S12; *K* = 5, log-likelihood ratio statistic = 22.3, *P* < 0.001). The same pattern was also apparent across a range of different numbers of ancestry components (*K;* *SI Appendix*, Fig. S12).

## Discussion

We have presented several lines of evidence that polymorphisms on the gene *lectin-24A* affect susceptibility to parasitoid wasp infection. First, in a QTL analysis, the marker that is most strongly associated with resisting infection falls within this gene. Second, in the resistant line, this gene is strongly up-regulated after infection, resulting in the production of a protein that localizes to the surface of the parasite. In the susceptible line, this gene is not up-regulated due to a mutation in a *cis*-regulatory sequence. Third, within populations, flies with haplotypes that have low expression are more susceptible to infection. Finally, when we introduce loss-of-function mutations by mutating *lectin-24A* in the resistant line, the flies become susceptible, and when we overexpress the protein, they become more resistant.

In nature, *Leptopilina* wasps are capable of infecting and killing 90% of *D. melanogaster* in some fruits in a single generation (51). Therefore, our finding that numerous loss-of-function alleles are segregating in a gene that protects flies against these parasites is unexpected. Furthermore, our population genetic analysis demonstrated that natural selection has favored null alleles of *lectin-24A* in southern Africa, suggesting that expressing this gene may sometimes reduce fitness.

Artificial selection experiments in *Drosophila* have shown that when populations evolve resistance to parasitoid infection, the fitness of uninfected flies is strongly reduced, indicating that alleles that increase parasitoid resistance can pleiotropically reduce other components of fitness (23, 52). This cost has been attributed to an increase in hemocyte numbers in genetically resistant flies (23, 52), and recent experiments have demonstrated that high hemocyte numbers reduce the accumulation of lipids in the fat body that are important during nutrient scarcity (53). Could *lectin-24A* also contribute to the cost of evolving resistance, explaining why natural selection has favored the loss of this gene? An argument against this idea is that this gene is strongly up-regulated after infection, and inducible expression is thought to avoid the production of costly gene products except in useful contexts (54). However, in the case of *lectin-24A*, lines with inducible haplotypes also have higher baseline expression, which could give rise to the cost of expression in the absence of infection, perhaps due to autoimmune damage.

Our conclusion that loss-of-function mutations in *lectin-24A* have a selective advantage in some populations may be an example of the “less is more” process, which postulates that gene loss and pseudogenization can be beneficial, particularly following drastic shifts in environmental conditions (55–57). As natural selection specifically favored loss-of-function alleles of *lectin-24A* in southern Africa, the balance of these costs and benefits appears to have shifted in different environments, resulting in the susceptible allele having an advantage. Interestingly, studies of *Diptericin A*, which confers resistance to gram-negative bacteria such as *Providencia rettgeri*, have also found that loss-of-function alleles segregate at higher frequencies in the south of Africa (58). In both cases, this may reflect differences in the parasite pressure. However, while the *L. boulardi* group is thought to occur in Southern Africa (59), the prevalence, genotype, and frequency of the parasitoids in that region are unknown. *lectin-24A* can be triggered by different wasp species (36, 60), and it is possible that this gene does not protect flies against all parasitoid species. Similarly, some genotypes of *L. boulardi* are highly effective at suppressing the immune response and are rarely melanized. These factors may mean that this immune defense may not provide any protection against infection in some populations, so functional alleles of this gene could reduce fitness due to pleiotropic effects. Alternatively, costs of resistance may only become apparent when food is scarce (23, 52), so geographical differences in selection may be due to differences in harmful pleiotropic effects of the resistant allele on some other trait. Pathogen defense could also employ a different pathway in this geographical region, for instance, via a protective symbiont such as *Spiroplasma* (61). If this is the case, losing a costly melanization response could be beneficial even in infected flies as the parasite might be killed in another way. If the melanization response against parasitoids is costly, then we might expect other genes involved in this process to be lost when parasitoid pressure is low. This appears to be the case in a species called *D. sechellia.* Lamellocyte-mediated encapsulation arose recently in the melanogaster subgroup, and this was associated with the appearance of 11 genes that are strongly induced after infection (62). Strikingly, three of these have presumed loss-of-function mutations in *D. sechellia* (62, 63). This includes two genes—*PPO3* (63) and *Tep1* (35)— known to have important roles in melanotic encapsulation. *Drosophila sechellia* is thought to have escaped from parasitoid infection by feeding on a fruit that is toxic to parasitoids (64). Therefore, in this species, the molecular machinery underlying the antiparasitoid immune response appears to have been lost over a short period of evolutionary time once it was no longer required.

*lectin-24A* appears to be a hotspot of adaptive evolution in the *Drosophila* immune system (47). It has arisen recently in the common ancestor of *D. melanogaster* and *D. simulans* and is one of the most rapidly evolving proteins in the genome (47). In *D. simulans*, there has been a recent and strong selective sweep (37, 47), while in *D. melanogaster*, it has exceptionally high geographical variation in allele frequencies (47). These observations suggest that it may be a key player in the coevolution of *Drosophila* and parasitoids.

*Drosophila* kills parasitoids using a cellular immune response. However, parasitoid infection also triggers a strong transcriptional response in the fat body, resulting in the secretion of humoral immune factors. The function of these molecules is largely unknown. *lectin-24A* is massively up-regulated following infection by *A. tabida* (36, 60) and *L. boulardi* (37, 38) but not by wounding or bacterial infection (37). We found that it localizes to the surface of the parasitoid egg before the attachment of hemocytes. It may function as an opsonin, binding to the parasite to promote hemocyte attachment. An understanding of *lectin-24A’s* molecular function may provide insights into why it evolves so fast and why it appears to reduce the fitness of flies in some populations.

## Materials and Methods

We screened the inbred DGRP lines (44, 45) for levels of melanization following parasitoid wasp infection and chose two lines showing a large difference in melanization rates. We mapped the locus responsible for differential melanization ability using QTL mapping. Then, we identified candidate genes by intersecting the genes occurring within the locus governing melanization ability with genes showing differential expression following exposure to parasitoid wasps (35). We confirmed the necessity of our candidate gene (*lectin-24A*) by knocking it down using CRISPR/Cas9-mediated targeted mutagenesis. We also assessed differential *lectin-24A* induction in the resistant and susceptible lines following parasitoid infection using qPCR. We investigated whether natural selection had a role in favoring null alleles in *lectin-24A* in global fly populations. A detailed description of the methods is in *SI Appendix*.

## Supplementary Material

Appendix 01 (PDF)

## Data Availability

Miseq reads for cDNA and gDNA for allele-specific expression of *lectin-24A* were deposited into the NCBI Sequence Read Archive under Bioproject PRJNA789229 (65). The DGRP-437 *lectin-24A* coding gene sequence and the germline Cas9 mutant sequence were deposited into GenBank: OM100576–OM100577. Scripts and processed data files are available in Zenodo (66). All other data are included in the manuscript and/or *SI Appendix*.
