# Supplementary material for: Natural selection has driven the recurrent loss of an immunity gene that protects Drosophila against a major natural parasite
Source: Proc Natl Acad Sci U S A. 2023 Aug 8;120(33):e2211019120. doi: 10.1073/pnas.2211019120 (PMC10438844; doi:10.1073/pnas.2211019120)
Supplement: Supplementary file 1 — Appendix 01 (PDF) [file pnas.2211019120.sapp.pdf]

PNAS

Supplementary Information for

Natural selection has driven the recurrent loss of an immunity gene that protects *Drosophila* against a major natural parasite

Ramesh Arunkumar [1†*], Shuyu Olivia Zhou[1†], Jonathan P. Day[1†], Sherifat Bakare[1,2], Simone Pitton[1,3], Yexin Zhang[1], Chi-Yun Hsing[1], Sinead O'Boyle[1,4], Juan Pascual-Gil[1,5], Belinda Clark[1], Rachael J. Chandler[1,2], Alexandre B. Leitão[1*], Francis M. Jiggins[1*].

[1] Department of Genetics, University of Cambridge, Downing Pl, Cambridge, CB2 3EH, UK
[2] Department of Biochemistry, University of Surrey, Guildford, Surrey, GU2 7XH, UK
[3] Biosciences Department, Università degli Studi di Milano, Via Giovanni Celoria, 26, 20133 Milano MI, Italy
[4] University College Dublin, Belfield, Dublin 4, Ireland
[5] Universidad Autónoma de Madrid, Ciudad Universitaria de Cantoblanco, 28049, Madrid, Spain

[†] Equal contribution

* **Corresponding Authors**: Ramesh Arunkumar (ar885@cam.ac.uk), Alexandre B. Leitão (alexandre.leitao@research.fchampalimaud.org), Francis M. Jiggins (fmj1001@cam.ac.uk)
Address for correspondence: Francis M. Jiggins, Department of Genetics, University of Cambridge, Downing Pl, Cambridge, CB2 3EH, UK
Phone number for correspondence: +44 (0)1223 333175

This PDF file includes:

Supplementary text
Figures S1 to S16
Tables S1 to S11
SI References

Supplementary Information Text

Extended Materials and Methods

Drosophila melanogaster stocks maintenance

We used the DGRP panel, which is a set of genome-sequenced inbred lines derived from a natural population in Raleigh, North Carolina, USA (1, 2). Based on preliminary data we selected two lines, DGRP-437 (Bloomington Drosophila Stock Center, BDSC#25194) and DGRP-892 (BDSC#28258) to serve as our resistant and susceptible lines in this study. Fly lines were maintained in glass vials on a cornmeal diet (per 1200ml water: 13g agar, 105g dextrose, 105g maize, 23g yeast, 35ml Nipagin 10% w/v). The vials were kept in a 25°C room with 70% of relative humidity and a light-dark cycle of 14 hour/10 hour. After a maximum of 20 days, adult flies were transferred into new clean vials.

Leptopilina boulardi stock maintenance

Leptopilina boulardi is an endoparasitoid wasp (Hymenoptera; Figitidae) infecting the larval stages of *Drosophila* (3). We used the low virulence *L. boulardi* strain G486 (Gif/Yvette, stock number 486) (4) created from an isofemale strain selected from a population of Brazzaville, Congo. We maintained wasps in the laboratory on an outcrossed population of *D. melanogaster* collected from Cambridge. To do so, *D. melanogaster* eggs laid overnight on agar plates in population cages were collected using phosphate buffed saline (PBS) and a bristle brush to ensure all eggs were removed from the agar plate and they were transferred into 15ml falcon tubes. Using a P1000 Gilson pipette with the end of the tips cut off, 500ul of the eggs were transferred into a 1.5ml microcentrifuge tube. Subsequently, a P20 Gilson pipette with the ends of the tip cut was used to transfer 6μl of eggs into plastic cornmeal food vials. After sex identification using the antennae size, 2-3 females and 1 male *L. boulardi* wasps were added to each vial. The vials were kept in a 25°C room with a relative humidity of 70% and a light-dark cycle of 14 hour/10 hour for at least 24 days. Once the adult wasps emerged, we administered CO_2 to anaesthetize them and put them into plastic cornmeal food vials supplemented with honey and kept at room temperature.

DGRP screen

We screened for parasitoid resistance in 194 DGRP lines. Due to the large number of lines, assays were split across multiple days. Each DGRP line was assayed on two separate days. On a given day, 15 unique DGRP lines were assayed. This set of 15 was chosen randomly without replacement. For each DGRP line, adult flies were allowed to lay eggs overnight on 90 mm plates (per plate: 0.565g agar, 0.625g glucose, 6.25ml apple juice, 18.75ml water, 0.375ml Nipagin 10% w/v) covered with yeast (Sigma-Aldrich: YSC2) mixed in water. Eggs were washed with PBS and transferred into 15ml centrifuge tubes. The resulting egg suspension was allowed to settle on the bottom of the tube. 15μl of eggs was transferred into 50mm diameter cornmeal plates. Plates were incubated at 25°C for 48 hours. Groups of 40 second instar larvae were gently transferred with forceps into each plastic vial with cornmeal food (one replicate) and immediately infected for 3 hours with 3 *L. boulardi* G486 females. 2-5 replicate vials per DGRP line were assayed on a given day. Tubes were then incubated for 10 days at 25°C. The adults that emerged were assayed for the presence of capsules and the melanization ratio was calculated with the following formula:

$$Melanization\ ratio = \frac{\#Flies\ with\ capsules}{40 - \#Non\ infected\ flies}$$

Scoring melanization rates in larvae – resistance assay

The female wasp lays its eggs inside the Drosophila larva's hemocoel through the ovipositor. If wasp larvae survive *Drosophila* immune defense, they will feed on its tissue and emerge from the pupae of the fly. If the immune response is successful, flies encapsulate wasp eggs, and this results in black melanized capsules easily visualized under a microscope.

We had used two different assay strategies in 2018 and 2019, which were largely similar except in how they controlled *D. melanogaster* larval density. For the resistance assays performed in 2018, 100-200 flies were maintained in population cages with apple agar plates covered with yeast mixed in water. The plates were changed every morning and evening. Plates from the first day after egg transfer were discarded. Plates containing eggs laid overnight from the following three days were retained and we performed larval transfer. Eggs on the surface of the 90mm agar plates were washed with PBS. This solution was poured into a 15ml falcon

tube, and the eggs were allowed to settle to the bottom. Using a cut P1000 tip, 500µL of solution saturated with eggs was transferred into a 1.5µL microcentrifuge tube. Then, five 30mm plates containing cornmeal were scratched in the middle. A cut P200 tip was used to transfer 13µL of solution from the microcentrifuge tube containing eggs to each 30mm cornmeal plate, in a line around the edge of the scratched area. The 30mm cornmeal plates were incubated at 25°C with a relative humidity of 70% and a light-dark cycle of 14 hour/10 hour. Two days after incubation, larvae were transferred from the plates into plastic vials containing cornmeal media using a pair of forceps under a Leica MZ6 stereomicroscope. From each plate, we attempted to make four vials containing 40 larvae each. We added three female wasps using a paintbrush to each vial after administering CO_2 to anaesthetize them. We incubated vials at 25°C for three hours to allow the wasps to infect the larvae and removed the wasps after that. Vials were kept at 25°C for another two days to allow them to develop.

We simplified the resistance assays in 2019 as we found we were able to attain a similar egg density by pipetting 5ul of eggs in PBS suspension from the 1.5µL microcentrifuge tube. Rather than using larger population cages, we placed ~50 flies in inverted 100ml laboratory plastic beakers with the opening covered with 30mm apple agar plates with yeast using the same recipe as above. Eggs from these plates collected into microcentrifuge tubes as above. We transferred 5ul of egg suspension per stock directly into plastic cornmeal food vials without explicitly controlling for number of larvae. The remaining steps were similar to the 2018 assays.

We dissected developed fly larvae to identify whether the wasp had survived or been melanized. To do so, we added 15% (w/v) sugar solution in ddH_20 and dissolved the cornmeal allowing the larvae to float to the top. We transferred individual larvae onto separate drops of ddH_20 on a clear plastic lid and dissected them using two pairs of forceps. We removed the cuticle of the larvae to check and allow for wasp larvae, if any, to settle to the bottom of the droplet of water. We scored flies as having melanized the wasp if the wasp larvae within the fly were all melanized and/or immotile. We score fly larvae as having failed to melanized if there is at least one non-melanized and motile wasp larvae in its body. We excluded fly larvae without any wasp as these were uninfected samples. We dissected 20 infected larvae from each vial. We used gloves during dissection and continuously cleaned the forceps during the dissection with ethanol.

Characterizing melanization of resistant and susceptible parental lines

We tested for difference in melanized rates between DGRP-437 and DGRP-892 using the method described above and analyzed the data with a generalized linear mixed model with a logit link function using the equation below:

$$\text{glmer}(\text{Melanization ratio} \sim \text{DGRP line} + (1|\text{Replica}), \text{family} = \text{"binomial"})$$

We also characterized the melanization response in the resistant and susceptible DGRP lines used for genetic mapping by injecting them with mineral oil containing homogenized parasitoid wasp. Twenty male *L. boulardi* and *A. tabida* were homogenized in 200µl of paraffin oil (Sigma-Aldrich M5904) using a pestle and injected into fly larvae using the protocol established in (5). Injected larvae were moved using forceps to cornmeal food vials where each vial contained 40 larvae. Vials were incubated in the 25°C room with a 14hr light-10hr dark cycle and 70% humidity for 10 – 48 hours depending on the scheduled dissection time. Prior to dissection, 15% w/v sugar solution was used to remove the larvae from food. The larvae were dissected on a PBS droplet that caused the injected oil droplets to float to the surface and the oil droplets were classed by their melanization state. The following binomial generalized linear model was built:

$$\text{glm}(\text{Melanization ratio} \sim \text{DGRP line} + \text{Time post injection}, \text{family} = \text{"binomial"})$$

The Wald χ^2 statistic was used to assess significance of the fixed effects. A likelihood ratio test comparing the above model to a more complicated model containing an interaction term between line and time post injection, dissection date, injection date, dissection time and injection time was not significant, so the simpler model was used.

Wasp embryo development in the resistant and susceptible DGRP lines was observed at 18-, 24- and 26-hours post *L. boulardi* infection. After performing the 2019 larval resistance assay protocol, the wasp embryo was isolated on a microscope slide and the *D. melanogaster* larval tissue was removed. A cover slide was placed on top of the reaction well and it was transferred into a humidity chamber. The images of the wasp embryo were taken on a Leica Microscope with an 8x1 objective.

Dominance of resistance genotype

We assessed the dominance of the resistance phenotype by crossing DGRP-437 with DGRP-892. We performed the crosses in two directions: virgin females of DGRP-437 were crossed to DGRP-892 males in one cross and vice-versa for the reciprocal cross. We used ~50 males and ~100 females for each cross and placed them in 135mm by 100mm (length by width) population cages. We tested for resistance to *L. boulardi* in the heterozygote progeny resulting from these crosses at the larval stage. We followed the 2018 protocol for the resistance assay and distinguished male and female larvae by the size of the developing gonads, seen as translucid region in posterior part of the fat body. We tested if parental genotypes (cross) and sex of progeny influenced melanized in the F_1s using binomial generalized linear mixed-effects model in the lme4 v1.1-23 R package (6). Wasp melanized was included as the binomial response variable. The following model was fit:

$$\text{glmer}(\text{Melanization} \sim \text{Cross} + \text{Sex} + (1|\text{Replica}), \text{family} = \text{"binomial"})$$

We performed Tukey's multiple comparison test on the fitted model using *glht* function from the multcomp v1.4-14 R package (7) and adjusted the resulting *p*-values using the Holm approach to compare the melanized levels in reciprocal crosses post-hoc. A likelihood ratio test was used to investigate if sex impacted melanization.

Chromosome substitution lines assay

We performed standard genetic crosses using DGRP-437 and DGRP-892 to generate lines with unique combinations of chromosomes from the susceptible and resistant parents to identify the chromosome(s) associated with melanization. We set up crosses to create six substitution lines carrying varying combinations of the X, II and III chromosomes from DGRP-437 and DGRP-892. Double balanced chromosome stocks used for the crosses, *f*/FM7 and w^*; *If*/CyO; MKRS/TM6B, were obtained from the Fly facility in the Department of Genetics, University of Cambridge.

To confirm the crosses were successful we genotyped the X, II and III chromosomes in the substitution lines using EcoR1 cut sites and primers designed by (1). Primers X2, 2L1 and 3L3 amplified regions that carried a cutting site that differed between DGRP-437 and DGRP-892 (Figure S13). After DNA extraction and PCR, we performed the EcoRI restriction digest. For the digest, we used 0.6µL EcoR1-HF (NEB: R3101), 3µL 10x NEBBuffer 2.1 (NEB: B7202S), 16.4µL nuclease-free water (Ambion: AM9930) and 10µL PCR product per sample. We performed the digest using a Veriti™ 96-Well Thermal Cycler with the following protocol: 1) 37°C for 1 hour and 30 minutes, 2) 65°C for 20 minutes. We ran the undigested and digested PCR products on a 2% agarose (Bioline: BIO-41025) gel in TBE with 4µL EtBr solution (Sigma-Aldrich: E1510). We used 6X loading dye (Termo Scientific: R0611) for loading the samples running it alongside Hyperladder 1kbp (Bioline: BIO-33053). The samples were run on a BioRad PowerPac™ HC submerged in TBE at 120mV for about 50 minutes.

We assayed resistance levels in the larvae for chromosome substitution lines and the parental DGRP lines using a mixture of the 2018 and 2019 approaches described above (see section: resistance assay). We tested for differences in resistance by fitting a generalized binomial (logistic) linear mixed model by maximum likelihood in the lme4 v1.1-23 R package (6). In our model, wasp melanization was included as the binomial response variable. The chromosome substitution line was included as a fixed effect. We included the assay vial ('replica') and date of dissection date as a random effect to allow for overdispersion. The latter factor would account for the differences in assay methodology in 2018 and 2019. The fitted model was:

$$\text{glmer}(\text{Melanization} \sim \text{Line} + (1|\text{Replica}) + (1|\text{Dissection date}), \text{family} = \text{"binomial"})$$

Further, we performed the Tukey's multiple comparison test on the fitted model using *glht* function from the multcomp v1.4-14 R package (7) and adjusted the resulting *P*-values using the Holm approach to identify the significant comparisons. We also tested for epistasis between chromosomes II and III using a multiplicative model (8). We fitted a glmer including the two autosomes and their interaction as fixed effects using the formula:

$$\text{glmer}(\text{cbind}(\#\text{resistant}, \#\text{susceptible}) \sim \text{chrII} * \text{chrIII} + (1|\text{Replica}), \text{family} = \text{"binomial"})$$

We performed an analysis of variances employing χ^2 statistic to assess the impact of the chromosomes and their interaction on melanization ratios.

DNA extraction using Chelex resin

We collected single flies in strip tubes, froze them at -18°C and performed DNA extractions subsequently. First, we prepared a 5% (w/v) Chelex solution (Sigma Aldrich: C7901) in ddH_20 and placed a magnetic bead within it. We autoclaved the mixture. We stirred the prepared Chelex solution on a magnetic stirrer (StarLab: N2400-3010) at 700rpm and added 150µL of solution using a cut P200 tip to each PCR tube as the solution was being stirred. Further, we added ~ six Zirconia 1.0 mm diameter beads (BioSpec: 11079110z) to each PCR tube. We then homogenized the flies using the TissueLyser II (Qiagen: 85300) for two minutes at 25Hz. We repeated the homogenization and checked that the flies were lysed at the end. We spun the tubes using a Beckman Coulter Optima™ MAX-XP tabletop ultracentrifuge at 1,000g for two minutes. Then, we added 1.5µL of proteinase K (Sigma Aldrich: P8044) at 20mg/mL in nuclease-free water to each sample and incubated the tubes at 56°C overnight. We spun the tubes again using the ultracentrifuge at 1,000 g for two minutes. We transferred ~100µL of supernatant into a new PCR plate taking care not to pipette any of the Chelex beads. We incubated the PCR plates at 94°C for 15 minutes to deactivate the proteinase K.

Primer design and PCR for genotyping

SNPs and indels that vary between DGRP-437 and DGRP-892 were used to genotype progeny resulting from crosses using the two parental DGRP stocks. We attained variant positions from the DGRP Freeze 2.0 website (http://dgrp2.gnets.ncsu.edu, last accessed May 2019). The DGRP Freeze 2.0 was mapped to version 5 of the genome but the recombination and physical maps from Flybase (FB2019_02) (9) are aligned to the version 6. Therefore, we converted the coordinates from version 6 to 5 using the *Coordinator Converter* function in Flybase. We searched for SNPs or indels 40 – 200bp long in each interval of interest. We then attained the sequence 1,000bp on either side of that indel. We used Primer3 (http://primer3.ut.ee, last accessed May 2019) to design primers to amplify the variant. We opted for products of sizes 300-1000 and only selected those that amplified the variant of interest.

In most cases, we performed PCR by mixing 0.5μL of 10μM dNTP (NEB: N0447), 2.5μL 10x ThermoPol® Reaction Buffer (NEB: B9004S), Taq DNA Polymerase (NEB: M0267X), 0.5μL of 10μM of forward primer, 0.5μL of 10μM of reverse primer, 18.875μL nuclease-free water (Ambion: AM9930) and 2 μL genomic DNA per sample. We performed the touchdown PCR reaction on the Veriti™ 96-Well Thermal Cycler with the following protocol: 1) 95°C for two minutes, 2) 10 cycles touchdown of 95°C for 30 seconds, 62°C to 52°C for 30 seconds where the temperature drops by 1°C every cycle, 68°C for seconds and 3) 25 cycles of 95°C for 30 seconds, 52°C for 15 seconds, 68°C for 30 seconds.

QTL mapping of phenotyped larvae

We performed QTL mapping to identify the genetic regions on the chromosome II associated with resistance to infection by the parasitic wasp. We crossed 100-200 female X^{892}; II^{892}; III^{437} and 100-200 male X^{892}; II^{437}; III^{437} to create F_1 individuals heterozygous for the second chromosome (X^{892}; $II^{437/892}$; III^{437}). We backcrossed 100-200 F_1 heterozygote males to 100-200 female X^{892}; II^{892}; III^{437} in a single 210mm by 100mm population cage to generate our recombinant mapping population. We then parasitized larvae in this mapping population following the 2018 resistance assay protocol. Once an infected larva was dissected and scored, we placed the dissected fly larvae into PCR tubes kept on ice taking care not to sample any melanized capsule or wasp larvae. Once we collected 24 samples, we moved the PCR tubes to a -18°C freezer. Then, we extracted the DNA following the Chelex DNA extraction protocol described above and kept them at -18°C. We designed primers to target indel polymorphisms in DGRP lines 437 and 892 to genotype multiple positions within chromosome II (Table S5). We ran the PCR products on a 1.2% agarose gel with EtBr for 45-60 minutes until the bands were well resolved. We took image of the gels with a BioRad Gel Doc™ XR+ to score the genotype of each sample varying exposure time between 3 and 9 seconds.

We performed QTL mapping using the standard genetic map of *D. melanogaster* from Flybase. We only used samples that were fully typed for all ten markers. We used the R/QTL v1.46-2 R package (10) for QTL mapping. We first calculated genotyped probabilities between markers, assuming a genotyping error probability of 0.05 and step size of 1 cM using the Haley-Knott method, and then used linear regression to test whether these probabilities were associated with the phenotype. We also performed composite interval mapping using a window size of 10. We used the LOD drop of 1.5 from the maximal LOD score to establish the interval on the genetic map that is likely to contain the gene affecting the phenotype.

High resolution QTL mapping with informative recombinants

To increase the resolution of our QTL, we performed additional genotyping of adults that melanized the wasp within the identified interval of interest. We performed the same cross as before in cages with the goal of generating large numbers of flies. We set up the cross three times in separate 210mm by 100mm population cages. For the first cage, we followed the same approach as was done to score resistant and susceptible larvae in 2018, which included both egg and larval transfer (see section: resistance assay). In the second cage, we had 50-100 female X^{892}; II^{892}; III^{437} and 50-100 male X^{892}; II^{437}; III^{437} and in the third one we had 100-200 female X^{892}; II^{892}; III^{437} and 100-200 male X^{892}; II^{437}; III^{437}. For the second and third cages, we collected eggs laid over a period of two weeks after each cage was first set up. We transferred 5μL eggs in PBS solution, collected from 90mm agar plates covered in yeast after cage set-up, directly to cornmeal plastic vials without yeast. In addition to using plates from the morning after an overnight egg lay, we transferred eggs collected on plates from the evening into plastic vials. On some dates, eggs lay occurred over a 24-hour period. For most vials, 2-4 days after egg transfer, we transferred one female wasp and placed it in the vials for 1-3 days. Vials were kept at 25°C with a relative humidity of 70% and a light-dark cycle of 14hr-10hr. We waited

for 13 days post egg transfer for all flies to emerge. We checked for flies with melanized capsules after administering CO_2 to anaesthetize them. We discarded flies without any capsules from the infected vials. We collected ~1,900 flies with melanized capsules from infected vials and ~500 control flies from uninfected vials. Flies were collected in PCR strip tubes and frozen at -18°C.

As uninfected adults occasionally produced melanotic nodules that can be mistaken by melanotic capsules, we tested these flies for wasp DNA using quantitative PCR (qPCR). We designed primers from a randomly chosen location of the *L. boulardi* genome (scaffold_2167) obtained from NCBI (ASM312160v1) (11). The forward primer was AGCAGCGATTGAAACAGTTGT starting at 16,463bp and the reverse primer was TGATTGTTGAACACGTCGGA starting at 16,679bp. Alongside melanized *D. melanogaster* samples, we performed qPCR on DNA extracted from *L. boulardi* strain G468 with Chelex beads and uninfected *D. melanogaster* flies. For each reaction, we added 5μL SensiFAST SYBR® Hi-ROX mix (Bioline: BIO-92020), 2.75μL nuclease-free water, 2μL of DNA and 0.125 μL of each of 10 μM forward and 10 μM reverse primers. We performed the reactions with MicroAmp™ Fast Optical 96-Well Reaction Plate with Barcode (Applied Biosystems: 4346906) and covered the plates with MicroAmp™ Optical Adhesive Film (Applied Biosystems: 4311971). We performed qPCR using the StepOnePlus™ Real-Time PCR System v2.2.3 (Applied Biosystems: 4376600) with the following protocol: 1) 95°C for two minutes, 2) 40 cycles of 95°C for five seconds and 60 °C for 30 seconds. Melt curves were obtained immediately following the step and hold protocol: multiple cycles of 15 seconds at 95°C and 60°C to 95°C for one minute where the temperature increased by 0.3°C every cycle. We analyzed melt curves using the StepOne™ v2.3 software. Wasp DNA samples amplified had a peak melting temperature close to 77-78°C whereas DNA from uninfected *D. melanogaster* samples did not amplify (Figure S14). Sample DNA were extracted with Chelex resin using the approach detailed above (see section: DNA extraction using Chelex resin). Wasp infection status in the F_2s were determined by comparing the melt curves to those obtained for the wasp DNA samples. We only retained samples that amplified wasp DNA for our analysis.

We extracted DNA from the flies that had melanized the wasp and uninfected flies and genotyped them using the 3 cM and 27 cM indel primers (Table S5) based on the interval attained from QTL mapping of resistant and susceptible larvae. Flies that were not typed at both markers were excluded. Following that, we selected flies that had melanized the wasp and had a breakpoint between 3 cM and 27 cM. For these flies, we also genotyped the indel markers 7 cM, 10.3 cM, 12 cM and 17 cM. For markers with missing values, we imputed genotypes if the flanking genotypes were the same.

We did additional Sanger sequencing using SNP based primers (Table S5) on samples with breakpoints in the interval of interest. In this case, we only performed Sanger sequencing for individuals where the flanking indel markers indicated there was a recombination breakpoint. We first did PCR as detailed previously (see section: Primer design and PCR for genotyping). Then, 10μL of primer product was cleaned using 1μL Shrimp Alkaline Phosphatase (NEB: M0371), 0.1μL Exonuclease I (NEB: M0293) and 3.9μL of nuclease-free water at 37°C for 1 hour and then the enzymes were inactivated at 80°C for 15 minutes. We then performed sequencing using the BigDye® Terminator v3.1 cycle sequencing kit (Applied Biosystems: 4337455). For each reaction, we added 1μL of BigDye® Terminator v3.1 Ready Reaction Mix, 2μL of 5X sequencing buffer, 2μL of cleaned PCR product, 4μL of nuclease-free water and 1μL of 3.3μM forward primer. We performed the sequencing reaction on the Veriti™ 96-Well Thermal Cycler with the following protocol: 1) 95°C for one minute and 2) 25 cycles touchdown of 95°C for 30 seconds, 50°C for 20 seconds and 60°C for four minutes. We sent the samples to Source Bioscience (https://www.sourcebioscience.com/, last accessed May

2019) for sequencing clean-up and Sanger sequencing. The sequenced chromatograms were analyzed using Geneious software v 11.0.3. Samples sequenced using the same primer were aligned. Low quality samples where multiple base calls were overlapping across the entire sequence were excluded. Heterozygous SNP variants were called when there were two clear base peaks at a given position. Following Sanger sequencing, we imputed missing genotypes based on flanking markers with the help of the zoo v1.8-8 R package.

CRISPR Cas9-mediated germline mutagenesis of lectin-24A

The necessity of *lectin-24A* for resistance to parasitoid wasps was tested by using CRISPR Cas9-mediated mutagenesis to generate lines with mutations or deletions in *lectin-24A* in the resistant background. Guide RNA (gRNA) constructs were designed to encode two separate gRNAs in duplicate using pCFD5-w as the backbone plasmid (pCFD5_w was a gift from Michael Boutros (Addgene plasmid # 112645); http://n2t.net/addgene:112645; RRID:Addgene_112645). gRNA for *lectin-24A* were identified using the Target Finder (12) against dm6 using high stringency and only allowing NGG PAM sites. Two sequences on chromosome 2L were selected both with 0 predicted off-target effects. The first sequence was between 3717384 and 3717406 (GGTCTCCA|AAGATTCATGCATGG) and the second sequence was between 3717584 and 3717606 (CATACTCC|ATAACTGGCTTCAGG). The two gRNA were separated by 178bp and occurred close to the start of *lectin-24A* coding region. Three fragments comprising tRNA, gRNA core and gRNA sequence were amplified using 10ng of pCFD5 as template in PCR reactions using the Lectin24a_PCR1,2,3 forward and reverse primers (using the protocol detailed in http://www.crisprflydesign.org/grna-expression-vectors/ and Table S6) and Q5 2xMaster Mix polymerase (NEB # M0492S). 600ng of pCFD5 was digested with BbsI restriction enzyme (NEB # R0539S). PCR amplified fragments and BbsI-digested pCFD5 vector were gel purified, quantified using Qubit HS DNA kit (Thermofisher # Q32854) and assembled using HiFi assembly master mix (NEB # E2621S) at 50°C for 1 hour with a 1:2:2:2 molar ratio of vector to the three fragments being cloned. Colonies were screened for correct sized (1.2 kbp) inserts using PCR with primers U63seqfwd and pCFDseqrev (Table S6), positive plasmids were purified and sequenced using the same primers. DNA for micro-injection of embryos was prepared using a plasmid midiprep kit (Qiagen # 12941).

To generate *lectin-24A* mutants we first generated a transgenic fly line expressing the two gRNAs. The pCFD5-w LectinKO gRNA construct was microinjected into *D. melanogaster* line attP2Ar5 expressing a phiC31 integrase under the control of *vasa* promotor on chromosome 4 and harboring an attP 2Ar5 landing site on the X chromosome (BDSC#24480) (13) by Bestgene Inc, USA. Mutations of *lectin-24A* were obtained by expressing Cas9 controlled by the *vasa* promotor (BDSC#51323). Transformants were used in the crossing scheme outlined in Figure S15 to generate germline mutations in *lectin-24A*. Individual males were screened by PCR for large deletions or insertions using primers that amplified the open reading frame of *lectin-24A*. Those products not exhibiting noticeable changes in PCR product length were sanger sequenced for mutations at the site of each gRNA. Through this, we obtained a mutant with a 4bp insertion at 129bp after the start codon (TTAT) and a 2bp deletion and a 1bp insertion at 328bp after the start codon resulting in 97 truncated amino acid protein (Figure S16) that we named as *lectin-24A*$^{\Delta 129}$. For the mutant and wild-type DGRP lines, larval melanization was scored using the methods above (see section: *Scoring melanization rates in larvae - resistance assay*) without controlling for larval density (the mutant lines produced very few larvae so there was little chance for overcrowding). 50-100 individuals were directly placed into a cornmeal vial for overnight egg lay. They adults were removed the next morning and larvae were infected with 2-3 parasitoid wasps at the 2/3 instar stage.

CRISPR Cas9-mediated somatic mutagenesis of lectin-24A

We created a fly line with the DGRP-437 background and expressing the Cas9 protein ubiquitously under the control of *Act5C* regulatory sequence (Act-Cas9; II^{437}; III^{437}) using a line expressing Cas9 on the X chromosome (BDRC#54590). We generated two different transgenic fly lines expressing the two gRNAs targeting *lectin-24A* in the pCFD5-w construct (see section: *CRISPR Cas9-mediated germline mutagenesis of lectin-24A*). The construct was inserted at the attP landing site of the X (2Ar5) or III (68A4) chromosome of the BDSC#24480 or BDSC#25710 line, respectively. We crossed females from each of these two lines expressing the guides to males expressing Cas9 in the DGRP-437 background (Act-Cas9; II^{437}; III^{437}) to create somatic knockouts in the F_1. The F_1 larvae were assayed for resistance to parasitoid wasp strain G486 using the methods above (see section: *Scoring melanization rates in larvae – resistance assay*) without controlling for larval density – the Cas9-expressing line does not produce many larvae so there was little chance for overcrowding. We placed around 15 females and 5 males into a cornmeal vial for overnight egg lay, and the adults were removed the next morning. Larvae were infected with three female wasps at 2/3 instar stage. The lines that were used for the microinjection were also crossed to the Cas9-expressing line to serve as controls in the assay. Only the female F_1 heterozygotes from the cross between the lines with X chromosome insertions and the Cas9-expressing line were used in the melanization assay.

To check whether somatic mutagenesis was successful, we pooled genomic DNA from five adult F_1 flies for the knockouts and two adult F_1 flies for the controls, using the Monarch® Genomic DNA Purification Kit (NEB: T3010L). The *lectin-24A* open-reading frame was amplified using primers: 5'- ATGTTTAGATTGTCAGTCTTAG - 3' and 5'-ATGCCATACTGGCATATG - 3' to generate a 845bp amplicon. We ran the PCR products on a 1% agarose gel and Sanger sequenced the products to check for mutagenesis (see section: *High resolution QTL mapping with informative recombinants*).

lectin-24A expression in DL2 cells

In order to express lectin-24A in *Drosophila* DL2 cells, it was cloned into the expression vector pMT-puro (gift from David Sabatini; Addgene plasmid #17923; http://n2t.net/addgene:17923; RRID:Addgene_17923) as a lectin-24A-Flag-strepII tag fusion. The open reading frame of lectin-24A was amplified from DGRP-437 genomic DNA using primers GGATCTAGATCGGGGTACACCACCATGTTTAGATTGTCAGTCTTAGTTCTGAACT TACTC and CTTTGTAGTCGATGCCATACTGGCATATGAATCTTTTTCATAAG that added a consensus kozak sequence and removed the stop codon. The Flag-StrepII tag was amplified from a plasmid containing the 3xFLAG-StrepTagII-mVenus-StrepTagII cassette (plasmid provided by D. Korona) (14) using primers TATGGCATCGACTACAAAGACCATGACGGTGATTATAAAGAT and CTGATCAGCGGGTTTTATTTTTCGAACTGCGGGTGGC. The expression vector pMT-puro was digested with the KpnI and PmeI restriction enzymes to remove the V5 and His-tags. The vector was then used in a HiFi assembly reaction with the two amplified fragments to form pMT-puro-lectin-24A-Flag-Strep. pMT-puro-Lectin24-A-Flag-Strep plasmid was purified from *E. coli* using a Qiagen miniprep kit (Qiagen: 27106) and used to transfect *Drosophila* DL2 cells in a final volume of 2.2ml media using Effectene transfection reagent (Qiagen 301425) according to the manufacturer's instructions. 48 hours after transfection, protein expression was induced by adding $CuSO_4$ to a final concentration of 1mM. After incubating for 24 hours, cells were removed by centrifugation and StrepII-tagged lectin-24A was precipitated from the media by incubating with 20ul washed Strep-Tactin XT beads (IBA life Sciences 2-4090-010) for 30 minutes. Beads were collected using a magnetic rack, then washed

twice in 1ml cold PBS supplemented with protease inhibitor before they were boiled in sample buffer +/- 1:100 beta-mercaptoethanol.

Visualizing lectin-24A localization

The transgenic lines HmlΔ-GAL4 UAS-GFP (BDSC#30140) was used to visualize hemocyte localization. *lectin-24A* fused with C-terminal *Msn*-mCherry (15) was used to visualize its expression in *Drosophila* larvae. To generate *Msn*-mCherry tagged *lectin-24A* constructs, pCFD5 was digested with XbaI and BglII to remove the dU6-3 promoter and gRNA scaffolds and the backbone vector was gel purified. A 3.4kbp genomic fragment encoding the open reading frame and a 2.5kbp *lectin-24A* promoter was amplified using Q5 polymerase 2x master mix (NEB # M0492S) with a touch down 62-52 PCR program: 95°C for 2 minutes followed by 10 cycles of 95°C for 15s, 62°C for 30s ramped down by 1°C every cycle, 72°C for 3 minutes followed by 25 cycles of 95°C for 15s, 52°C for 30s, 72°C for 3 minutes. Primers CCATTTAGCCGATCAATTGACGAGTACTTCTACGCCGC and CTTTGTAGTCGATGCCATACTGGCATATGAATC that incorporated sequences complementary to the vector and mCherry at the 5' and 3' end of the fragment, respectively, were used to allow for Gibson assembly. The template in the PCR was genomic DNA isolated from either the DGRP-437 or DGRP-892 lines. Similarly, mCherry with a FLAG-tag was amplified using primers GTATGGCATCGACTACAAAGACGATGACGACAAG and TTTAACTGGCTACTCGTCCATGCCGCC. The 3'UTR of *lectin-24A* was amplified using primers GGACGAGTAGCCAGTTAAAAACCAAAAAAAATC and CTGTTGCCGAGCACAATTGTCATTTCTTCATTTCGCCAC. Amplified fragments were gel purified, quantified using Qubit HS DNA assay kit (Thermofisher # Q32854) and used in a HiFi assembly reaction (NEB # E5520) with digested pCFD5 using an equimolar ratio of vector to fragments with a 1-hour incubation at 50°C. Plasmids were sequenced with internal primers used to amplify the fragments. The vermillion marker was then excised by digesting plasmids with HindIII and replaced with the mini-white gene purified by digesting plasmid pWallium20 backbone plasmid (https://fgr.hms.harvard.edu/publications/transgenic-rnai-project-harvard-medical-school-resources-and-validation) with HindIII. The construct was injected into *D. melanogaster* line attP2Ar5 by the Fly Facility (Department of Genetics, University of Cambridge, UK) and transformants were selected by eye colour.

To generate the secreted-mCherry construct with the DGRP-437 *lectin-24A* promoter, the *lectin-24A* open reading frame was removed from the DGRP-437 Lectin-FLAG-mCherry construct using the Q5 site-directed mutagenesis (NEB# E0554S), with the primers GACTACAAAGACGATGAC and GGTTCCTGCCGAGAATTC.

We extracted the mCherry-tagged lectin-24A to ensure that it remained intact following the fusion. To do this, early third instar larvae from transgenic lines containing DGRP-437 Lectin-FLAG-mCherry or DGRP-892 lectin-24A-FLAG-mCherry were infected with *L. boulardi* G486 or left uninfected for 24 hours. Larvae were harvested, washed in PBS, dried on filter paper, and homogenized in 20ul of lysis buffer containing 1% IGEPAL, 10mM Tris pH 7.5, 150mM NaCl supplemented 1 tablet to 50ml with protease inhibitor (Roche: 04 693 159 001) by grinding with a pestle. Samples were centrifuged at 17000g at 4°C for 5 minutes to pellet debris and the supernatant was removed to a fresh tube. Protein was quantified using Bradford Assay. 20ug was added to 4ul of 4xSDS loading buffer supplemented with 1:25 beta-mercaptoethanol.

Western blotting

Presence of intact lectin-24A protein, mCherry tagged lectin-24A, and oligomerization was verified through western blotting. Samples were separated by SDS-PAGE using a NuPAGE 4-

12% Bis-Tris Gel (Thermo Fisher: NP0322) including a lane containing PageRuler plus pre-stained protein ladder (Thermofisher: 26619). Protein was transferred to Amersham Protran 0.45 um Nitrocellulose blotting membrane (GE Healthcare: 10600008) and blocked in TBST with 5% milk for 30 minutes. Anti-FLAG M2 monoclonal antibody (Sigma: F1804) was diluted 1:5000 in 5% milk in TBST and the membrane was incubated with this over-night at 4ºC. After washing, the membrane was incubated with IRDye 800CW Goat anti-mouse secondary (LiCor: 326 32210) (1:5000 dilution in TBST with 5% milk). To determine loading quantities or larval protein samples membranes were stripped and re-probed with anti-a-tubulin primary antibody (Sigma: T6199) diluted 1:10000 in 5% milk, washed and incubated with IRDye 800CW Goat anti-mouse secondary. Membranes were detected using Li-Cor Odyssey XF Imager for 10 minutes detection time for the 800nm wavelength and 2 minutes for the 700nm wavelength.

Confocal microscopy imaging of wasp egg with tagged lectin-24A

We dissected wasp eggs at 2-12 hours post infection from heterozygous F_1 larvae of the following crosses: (1) lectin-24A-mCherry females x HmlΔ-GAL4 UAS-GFP males (2) secreted-mCherry females x HmlΔ-GAL4 UAS-GFP males. Wasp eggs were mounted in PBS between two cover slips with a spacer (0.12mm in depth) and imaged using a Zeiss LSM 980 confocal microscope with a 20x objective. The laser intensities and detector gains were the maintained for all treatments. Several images per egg were obtained with 1µm z-step to cover the egg volume, and maximum projection images for the fluorescent channels were obtained in ImageJ.

Over-expression of Flag-tagged lectin-24A in larvae

The *lectin-24A* open reading frame with a C-terminal Flag-tag was cloned into the pUAST attb GFP expression vector. pUAST attb GFP was digested with BglII and KpnI restriction sites, excising the GFP open reading frame which was removed using gel purification. The *lectin-24A* open reading frame with a 3' Flag-tag was amplified from the DGRP-437 *lectin-24A* mCherry plasmid using the primers GAATTGGGAATTCGTTAACAACCACCATGTTTAGATTGTCAGTCTTAG and CAAAGATCCTCTAGAGGTACCTACTTGTCGTCATCGTCTTTG. We used the Q5 polymerase (New England Biolabs #M0492) to incorporate a 5' Kozak consensus sequence and leave overhangs that enabled HiFi assembly with the digested vector. The *lectin-24A* flag-tag PCR fragment was assembled with the digested pUAST attb vector using HiFi assembly (New England Biolabs #E2621) following the manufacturers recommendations to make pUAST-attb-*lectin-24A*-Flag. This plasmid was microinjected into *D. melanogaster* line attP2Ar5 expressing a phiC31 integrase under the control of vasa on chromosome 4 and harboring an attP2 site on the X chromosome (13). Germline transformants were selected using mini-white marker. Transformants were made homozygous and for resistance assays were crossed to GAL4 expressing line C7 that expresses GAL4 in the larval fat body (16). Larvae were infected and assayed for resistance to *L. boulardi* strain G486 with the same method as for the DGRP screen. To control for larval density in the resistance assays, around 5 males with 35 females were maintained in small cages with apple agar media on 50 mm plates covered with yeast paste. The flies were allowed to carry out egg lay overnight for about 18 hours, and eggs on the surface of the agar plate were washed off with PBS then transferred to a 15ml falcon tube and the eggs were allowed to settle to the bottom. 500µL of solution saturated with eggs were transferred into a 1.5µL microcentrifuge tube. Eggs were allowed to settle to the bottom of the tube again, then 6µL of egg solution containing about 30 eggs is transferred to plastic vials containing cornmeal media, and larvae were allowed to develop for two days before infection.

lectin-24A promoter indel haplotype characterization

We Sanger sequenced the upstream and coding regions of *lectin-24A* in 171 DGRP lines as indel variants in *lectin-24A* were not identified in the genome sequences from DGRP Freeze 2.0. We amplified a 603bp fragment (dm6:2L:3717682-3718285) using the GGCGCCTCCTTCCACTATTT (forward) and TGGCTAACAAGGAGTAAGTTCAGA (reverse) primers. *lectin-24A* coding sequence and neighboring regions were subject to Sanger sequencing as detailed above (see section: Fine mapping with resistant recombinant adults) in 171 DGRP lines. The primers were designed by Primer3 (http://primer3.ut.ee, last accessed July 2019) using the upstream and coding sequence of *lectin-24A* obtained from Flybase (FB2019_02)(9). In some DGRP lines, heterozygous sites were encountered and, therefore, regions downstream of these sites were not properly sequenced. Sequences were aligned using Sequencer v.4.5 with the Dirty Data algorithm and default parameters. The c.-334_-333insACATTCAT indel alignments were manually curated for consistency with DGRP Freeze 2.0 (2L:3,718,040).

Having identified previously unknown indel variants, we identified the ancestral state by aligning the upstream indel variants and coding regions to closely related species. Orthologues of *lectin-24A* were obtained from Ensembl Metazoa (accessed 19-11-2020) for *D. sechellia* (dsec_caf1\GM18119) and *D. simulans* (ASM75419v3\GD22727). These were the only two species containing one-to-one orthologues with > 75% identity. The gene region and 2,000bp upstream were obtained for each species. Then, the *D. sechellia* and *D. simulans* sequences and were aligned with Sanger sequences for DGRP-437 and DGRP-892. Geneious 11.0.3 was used for alignments using the native global alignment algorithm with free end gaps, 51% similarity cost matrix and gap open and extension penalties of 50 and 6, respectively. A total of five iterations were done with a guide tree used for alignments. We also identified the ancestral state for the 300bp promoter region immediately upstream of the resistant DGRP-437 *lectin-24A* start codon. We performed a nucleotide megaBLAST (17) using the 300bp region as a query against the genomes of *D. melanogaster* (v6, NCBI: AE014134.6), *D. simulans* (Prin_Dsim_3.1, NCBI: NC_052520.2), *D. sechellia* (ASM438219v1, NCBI: NC_045949.1), and *D. mauritiana* (ASM438214v1, NCBI: NC_046667.1). We extracted the top hit for each species and performed a multiple sequence alignment using MUSCLE (18). No significant BLAST hits were found for >50% of this 300bp region in other members of the Drosophila genus.

lectin-24A fluorescent reporter constructs

lectin-24A promoter reporter constructs were made using the pWallium20 backbone plasmid. An 8,494bp fragment of pWallium20 was created by restriction digest using HpaI and PstI enzymes (NEB # R0105S and R0140T) followed by gel purification and quantification using a Qubit HS DNA assay kit (Thermofisher # Q32854). The DGRP-437 (489bp upstream on translation start site) or DGRP-892 (453bp upstream on translation start site) *lectin-24A* minimal promoter and was amplified using primers Lec500promF and lecpromvenusRv with Q5 2xMM PCR reagents (NEB # M0492S). The Venus open reading frame incorporating the ATG start codon of the *lectin-24A* open reading frame was amplified using primers venusLecPromF and venusLecPromR using pDsRed-attP (Addgene: Plasmid #51019) as a template. The pWallium20 backbone and the two PCR fragments were then assembled using a HiFi assembly reaction (NEB # E5520) using an incubation at 50°C for 1 hour. Transformation plasmids were then purified by miniprep (Qiagen # 27104). Plasmid sequences were verified by sanger sequencing with primers Lec500PromRv, venusLecPromR and venusLecPromF. *lectin-24A* promoter (LP) constructs containing different allelic combinations of the three upstream indels and six upstream SNPs (3717919_SNP, 3717922_SNP, 3717950_SNP,

3717956_SNP, 3718036_SNP, 3718037_SNP) that were polymorphic between DGRP-892 and DGRP-437. DGRP-892 alleles were substituted into the DGRP-437 LP sequence (LP437) by amplifying PCR fragments using primer combinations and template DNA as shown in Table S7. The fragments were then assembled with HpaI and PstI digested pWallium20 as for LP437 and LP892. All constructs (Table S8) were injected into *D. melanogaster* line attP2Ar5 and transformants were selected by eye color.

The regulatory activity of each lectin promoter reporter construct was evaluated by preparing samples of larval tissue lysate. At least eight independent samples were made for each promoter construct. Fifteen third instar larvae are collected for each sample, and tissuelysed in 100ul of PBS with about ten 1.0mm diameter zirconia/silica beads (Thistle Scientific # 11079110z) using Qiagen TissueLyser II (Qiagen # 85300) for 2 minutes at 30Hz. The samples were immediately spun down at 4000rpm for 10 minutes at 4ºC, and 50ul of the supernatant for each sample were transferred into a well in a flat clear-bottom black polystyrene 96-well plate (Corning # 3603) for fluorescence reading with a SpectraMax iD3 Plate Reader (Molecular Devices) using the SoftMax Pro 7 software. Venus fluorescence intensity is measured with excitation at 485nm and emission at 535nm. The rest of the supernatant for each sample is used to measure the total protein level of the sample with a Bradford assay (Merck # B6916), following reagent protocol. The total protein level was used as a normalization of Venus intensity. The whole larvae images were taken with a Leica MZ16F Fluorescence Stereoscope fitted with a GXCAM HiChrome-S color camera using the GXCAPTURE software captured at a 5ms exposure time. The GFP2 filter was used with an excitation of 480/40nm and emission measured at 510nm.

The assays took place over five days. We fitted a mixed effect linear model with relative Venus quantity as the response variable, the construct as a fixed effect and the experimentation date as a random factor. We performed the Tukey's multiple comparison test on the fitted model using *glht* function from the multcomp v1.4-14 R package (7) and adjusted the resulting *p*-values using the single-step method to identify the significant comparisons.

Transcription factor binding sites in lectin-24A promoter region

We scanned for the presence of putative transcription factor binding sites of three major immune pathways in the 498bp upstream region of *lectin-24A*. We used SnapGene (www.snapgene.com) to search for the binding site consensus motifs for Toll/NF-κB (Dif/dl – GGGHHNNDVH) (19), Imd/NF-κB (Rel – GGRDNNHHBS) (19), JAK/STAT (Stat92E – TTCNNNGAA) (20), and GATA (rp/pnr/GATAd/GATAe – WGATAR) (21) in the DGRP-437 and DGRP-892 *lectin-24A* promoter regions.

Allele-specific expression of lectin-24A in DGRPs

In order to understand the impact of upstream indels on expression, we selected multiple DGRP lines with varying combinations of the three upstream indels and tested if parasitoid infection upregulated *lectin-24A* in those lines. 10 male flies from 20 DGRP lines with varying combinations of the upstream *lectin-24A* promoter indels were crossed with 10 virgin females of DGRP-437. Females were allowed to lay eggs overnight in cornmeal vials with yeast. Adult flies were removed the next day and tipped into a new vial, which served as the second biological replicate. F_1 larvae in each vial developed for an additional 48 hours before they were infected with three female *L. boulardi* wasp strain G486 for three hours. 24 hours post infection, 10 larvae for each sample were homogenized in RTL buffer and RNA was extracted using an RNA easy miniprep kit (Qiagen: 74104), including optional on-column DNase treatment (Qiagen: 79254). cDNA was prepared using 1µl of template RNA with the GoScript Reverse Transcriptase system (Promega: A5003) primed with random hexamers, according to

the manufacturer's instructions. For each F_1 cross, genomic DNA (gDNA) was also extracted from eight adult flies using the Blood and Tissue DNA extraction kit (Qiagen: 69504).

Sequencing libraries were prepared from each sample. PCR was performed (see section: Primer design and PCR for genotyping) on the gDNA for one of the biological replicates and cDNA from both biological replicates targeting the *lectin-24A* gene region on chromosome 2L between 3,717,069 and 3,717,568 (dm6). *lectin-24A* sequences obtained from Flybase (FB2020_03) were used to design primers TCGTCGGCAGCGTCAGATGTGTATAAGAGACAGACAAATGGAATACTTGTACGGAAAT (forward) and GTCTCGTGGGCTCGGAGATGTGTATAAGAGACAGGACGCCCAGGAAGTATGATT (reverse) that include adapter overhang sequences. Two technical replicates of each PCR reaction were performed. PCRs were performed using Q5 DNA polymerase (NEB: M0491S) in a total volume of 50µl using 2µl of template cDNA or gDNA. PCR amplicons were cleaned using 20µl of Ampure XP beads (Beckman: A63880) and eluted in 52.5µl of 10mM Tris, pH8. An index PCR reaction was carried out using 5µl of cleaned PCR as template, 25µl of 2 x Kapa HiFi hot start PCR mix (Roche: KK2601), 5µl of each of Truseq XT index 1 and 2 obtained from the Nextera XT DNA Library Prep Kit (Illumina: 15052165) and 10µl of water. After cleaning with 56µl of Ampure beads the libraries were eluted in 27.5µl of Tris pH 8.0 and analyzed/quantified by agarose gel electrophoresis. Libraries were pooled and sequenced on MiSeq nano 300 cycle kit, paired-end sequencing at the DNA sequencing facility at the University of Cambridge Biochemistry Department.

Following sequencing, Illumina BaseSpace Sequence Hub was used to convert Binary Base Call files into FASTQ files for all reads passing filtering. We mapped sequenced reads to a FASTA sequence containing the region of interest obtained from Flybase (FB2020_03) using bwa-mem (22) marking shorter split hits as secondary. We also mapped reads to references incorporating alternate alleles for eight SNPs segregating in the 21 DGRP lines that we tested to estimate read mapping bias (Table S9). Variant calls for the parent DGRP lines were obtained from DGRP Freeze 2.0. Aligned reads were reordered and read groups were added using Picard Toolkit v.2.8.1 (23) and the resulting files were sorted using samtools 1.9 (24). The aligned and processed reads were indexed with Picard Toolkit. We called variants and estimated allele depth in the F_1 gDNA and cDNA using GATK3.7-0 HaplotypeCaller (25). Reads were assigned to DGRP alleles using a SNP (dm6, 2L:3717102) where all tested DGRP lines had the reference allele and DGRP-437 had the alternate allele (Table S9). A Welch two sample t-test was used to test for differences in allelic expression in the F_1 hybrids generated from DGRP-437 and DGRP-892. Indel haplotypes for tested DGRP lines were obtained from the Sanger sequencing calls and association between indel haplotype and allele expression was tested using a χ^2 test. Further, we tested for association between the counts of reference and alternate counts and indel haplotype for cDNA using a quasibinomial GLM.

Using *lectin-24A* expression estimates in adult flies from (26), we built a linear model regressing $\log_2$FPKM on *lectin-24A* upstream indel haplotypes and conducted a Tukey's honest significant difference test to identify differences in melanization rates between DGRP lines with varying haplotypes.

qPCR assays of lectin-24A expression in DGRP lines

We estimated gene expression using qPCR in separate group of 18 DGRP lines with varying indel haplotypes, partially overlapping with the ones referred to above for studying allele-specific expression. We used the 2019 infection assay (see section: resistance assay) and RNA extractions and cDNA protocols were the same as above. Primer3 (http://primer3.ut.ee, last accessed August 2020) was used to design the GATACTGCTTCCCCACCCTG (forward) and

ATCTGATCCTTGCGGGCTTC (reverse) primers to amplify a 108bp fragment in the intron-less *lectin-24A* coding region. RNA was incubated with DNase I (Qiagen RNase-Free DNase set: 79254) for 15 minutes per manufacturer protocol and qPCR was done as described for wasp fragment amplification (see section: Fine mapping with resistant recombinant adults). In addition, we included a 'no RT' (NRT) control in which the RNA was not reverse transcribed as a control for residual DNA contamination. Two replicates were performed per line and the average expression was taken. The expression of the housekeeping gene *RpL32* was estimated with the aid of primers obtained from (27) and ΔCT was calculated as $CT^{lectin\text{-}24A} – CT^{RpL32}$. -ΔCT is an estimate of the $\log_2$ relative *lectin-24A* expression. A linear regression was done to test the significance of the association between indel haplotype and $\log_2$ relative *lectin-24A* expression. A Tukey's honest significance test was done to compare $\log_2$ relative *lectin-24A* expression between indel haplotypes.

We performed a separate qPCR experiment to test for differential candidate gene induction following parasitic wasp infection in DGRP-437 and DGRP-892 using the same methods as above, excluding NRT controls, and custom designed primers (Table S10). We made four replicate measurements per gene per DGRP line per treatment. We tested for a significant effect of the interaction between the DGRP line and the infection treatment on candidate gene expression using an analysis of variance. For *lectin-24A*, we also did an analysis of variance on the model below to test the significance of the fixed effects on ΔCT.

$$\mathrm{lm}(\Delta \mathrm{CT} \sim \mathrm{Line} + \mathrm{Infection\ treatment} + \mathrm{Time\ post\ wasp\ exposure})$$

Variant identification in Drosophila Genome Nexus samples

We used *Drosophila* Genome Nexus data generated and complied by (28) to investigate molecular evolution of *lectin-24A*. First, fastq sequence data was obtained from NCBI SRA using SRA toolkit v2.9.6-1 (http://ncbi.github.io/sra-tools/). Then, Trimmomatic v.0.36 (29) was used to clip adaptor sequences and remove low quality bases from reads. We clipped adaptors sequences using reference file TruSeq3-PE.fa with the following parameters: seed mismatches = 2, palindrome clip threshold = 20, simple clip threshold = 10, minimum adapter length = 1 and keepbothreads = TRUE. For single end reads, we used the reference file TruSeq3-SE.fa. Further, we removed the last three bases from reads, filtered strings of low-quality bases found in sliding windows of sizes 4bp where quality dropped below 20 and ensured reads had a minimum size of 36bp. We mapped trimmed reads to the *D. melanogaster* reference (r5.13) (30) attained from Flybase (FB2019_02) (9) using bwa-mem (22) and reordered and added read group information specifying the sample and library names and Illumina planform using Picard Toolkit v.2.8.1 (23). Samtools 1.9 (24) was used to retain mapped reads with alignment quality above 20. For paired end data, we only retained properly paired and mapped reads. Reads were subsequently sorted by coordinate. Then, duplicate reads were marked and resulting alignment files were indexed using Picard Toolkit. Reads near indels were realigned and mis-encoded quality scores were fixed for some samples using GATK3.7-0 (25). CRISP v0.7 (31) was used to call variants in all samples jointly using a poolsize of 1. Further, the following default parameters were used: quality value offset = 33 for Sanger format, minimum base quality to consider a base for variant calling = 10, minimum read mapping quality to consider a read for variant calling = 20, minimum number of reads with alternate allele required for calling a variant = 4, log10 threshold on the contingency table *p-value* for calling position as variant = -3.5, log10 threshold on the quality values based *p-value* for calling position as variant = -5, maximum number of permutations for calculating contingency table *p-value* = 20000, filter reads with excessive number of mismatches and gaps compared to the reference sequence = TRUE, identify overlapping paired-end reads and treat

as single read in the overlapping region = TRUE, reference allele bias for targeted sequencing data = 0.5, EM algorithm will be used for estimating pooled genotypes and calling variants = TRUE, PCR duplicates will be ignored = TRUE. The reference genome was split into 11 regions to perform variant calling in parallel. SnpEff v4.3t (32) was used to annotate variants using the dm5 database. *lectin-24A* region SNPs were subset from the main variant call format (VCF). Preliminary investigations indicated a large deletion occurring in the *lectin-24A* coding region in some samples. This prompted us to use pindel v0.2.4s (33) to systemically call deletions in the *lectin-24A* region alignment files. Indeed, a 171bp region (TCCTGGGCGTCAACGAAAACACAAAAACCGGTGACTTTGTGTCTGCAGCCTCCG GAAAAAGTTGTTTGTATCACGAGTGGGGTCCTGGTGAACCCCATCACAATAATG ACCAAGAGCGATGCGTTTCAATCTTGCGAAAACTCATGCATGTGGGCAATTGTAC TTATGAAA) was substituted with a 6bp sequence (TAATCAT) near the 3′-end of *lectin-24A* in some lines, which results in a premature stop codon following the first base in the substituted sequence. We identified samples as having the coding deletion if they were fully genotyped for regions flanking the coding indel but did not contain any variant calls in the region with the presumed deletion. We confirmed calls by using pindel to call the deletion and by manually checking alignment files for split reads. The deletion calls were manually checked using Integrative Genomics Viewer v2.9.2 (34) for split reads. The deletion calls were added for further analyses. In order to genotype samples for the three upstream indels of *lectin-24A*, trimmed reads were mapped to a reference modified using GATK3.7-0 FastaAlternateReferenceMaker to include the insertions for all three indels and the above alignment processing steps were repeated. *lectin-24A* region alignments were isolated from the main file after coordinate-based sorting and downstream processing steps and variant calling was performed.

Variants that were identified were masked and quality-checked using the R script dgn_lectin.R *lectin-24A* region SNPs and the upstream indels were combined into a single file. Coding and noncoding region SNPs were named using protein and DNA based HGVS nomenclature, respectively, obtained from SnpEff annotations with the aid of the R package sjmisc (35). Variant names were modified to reflect ancestral and derived alleles rather than reference and alternate alleles. Indel names were manually given using HGVS rules and upstream SNP positions were modified to account for the ancestral upstream insertions, which are not found in the unmodified *D. melanogaster* reference. SRA run names were converted to sample names using the individual description provided by (28). For individuals with multiple SRA associations, genotype calls made in any of those sequencing files were retained unless they conflicted with each other. Multiallelic sites were only retained if all alleles had the same effect as predicted by SnpEff. The very recent migrant ZW184 was removed from population genetic analyses (see (36)). Regions identified as being affected by identity by decent, admixture or heterozygosity by (28) were masked from analyses. Following sample-specific site masking, individuals with more than 50% missing data for *lectin-24A* region variants were removed from analyses. Only the 26 populations that each contained more than three samples were retained for the analyses. Further, only variants that had an allele frequency >0.05 in two or more populations and with genotype quality greater than or equal to 10 were retained.

The consistency and reproducibility of the variant calls were confirmed by comparing to the constituent variant call datasets for subsets of the *Drosophila* Genome Nexus through Sanger sequencing. There was an exact match of variant calls using the approach detailed above and the calls published in DGRP Freeze2.0 (1, 2) and the Global Diversity Lines (36) (Table S11). Further, Sanger sequencing (using approach detailed in section: Fine mapping with resistant recombinant adults) of a subset of lines from the SD and SP populations from Southern Africa, received through the courtesy of John Pool, indicated an exact match for key upstream indels

and loss-of-function variant calls including the 165bp deletion segregating in some lines (Table S11). The following forward (GGCGCCTCCTTGGACTATTT) and reverse (CTGTTGCCGAGCACAATTGTCATTTCTTCATTTCGCCAC) primers were used for amplifying the fragment for sequencing. We performed qPCR for *lectin-24A* expression following infection and no infection of 14 lines from Southern Africa containing one or more of the presumed loss-of-function mutations in the *lectin-24A* coding sequence. Our goal was to test if any loss of expression mutations occur alongside the loss-of-function mutations, nullifying any effect of the latter. The method used in section '*qPCR assays of lectin-24A expression in DGRPs'* was repeated for qPCR assay.

Drosophila Genome Nexus population genetics analyses

Ancestral and derived status for variants in the 200 kbp surrounding *lectin-24A* was obtained from dm5 using samtools. Using species-specific BLAST from Flybase (FB2019_02) (9), the orthologous regions in *D. sechellia* and *D. simulans* were obtained. Sequences from the three species were then aligned using MAFFT as implemented on the EBI server (37). Following multi-species alignment, the allele that also occurred either in *D. sechellia* or *D. simulans* was designated as the ancestral state. However, if both the reference and alternate alleles or neither of them were found in the other two species, an ancestral state classification was not given, and these sites were removed from analyses.

VCFtools v0.1.15 (38) was used to calculate per-site Weir and Cockerham F_{ST} and weighted pairwise F_{ST} along the entire *lectin-24A* coding region between geographic regions. Gene diagrams for the per site F_{ST} plot was generated using the R package gggenes v0.4.1 (https://wilkox.org/gggenes/index.html). In order to calculate the significance threshold, per site F_{ST} was calculated for variants that occurred in bases 8-30 of short introns (50 to 65bp) as they are most likely to be evolving neutrally (39). The same variant processing, masking, and filtering steps as done for *lectin-24A* region-specific analyses were applied for the silent SNPs. However, no window-level masking for uncalled genotypes was applied and 1,039 individuals from the eight geographic regions were used for calculating the F_{ST} for silent SNPs. F_{ST} was only calculated when there were at least 22 populations each containing at least four samples genotyped for a given site. The top 1% and 0.1% F_{ST} percentiles were calculated using 23,635 variants in short introns.

For subsequent *lectin-24A* specific analyses, only populations that contained four or more individuals where at least 50% of the *Lectin-24* region was genotyped were used resulting in 672 individuals from 26 populations being retained for the analyzed. *Lectin-24* coding region F_{ST} was obtained using VCFtools. The R packages adegenet v2.1.3 (40) and hierfstat v0.5-7 (41) were used to calculate population-level allele frequencies. Pairwise linkage disequilibrium between sites was estimated using the R package Ldheatmap v1.0-4 with input VCFs read and processed into a genotype matrix using the R package VariantAnnotation v1.36 (42).

A neighbor-joining was constructed to infer the relatedness between samples. Processed genotype calls were formatted into a VCF. An indexed *D. melanogaster* reference was used alongside bcftools v1.11-21 (43) consensus to attain a reconstructed coding region alignment for all analyzed samples. A neighbor joining tree was constructed using the reverse complement of the alignment with the software MEGAX (44). The tree was constructed using a Jukes Cantor substitution model. The best fitting substation model was assessed using the R package phangorn v2.5.5 (45). The R package APE v5.4-1 (46) was used to read and process alignment files. For tree construction, uniform rates were applied for all sites and the pairwise deletion option was used for dealing with gaps and missing data. Trees were further processed in R using the package ggtree v2.4.1.

Population branch statistic

We estimated the PBS to test for positive selection on premature stop codons in *lectin-24A*. We identified the ancestral state of stop gained mutations by extracting 100bp on either side of the mutation using samtools. These query sequences were used to perform a nucleotide BLAST using the discontiguous megaBLAST (17) with the subject organism restricted to either *D. sechellia* or *D. simulans*. Alleles for sites containing premature stop codons were designated as the ancestral state if they occurred in either *D. sechellia* or *D. simulans*. Only premature stop codons designated as being the derived state in *D. melanogaster* were used in our analyses. The gene transfer file for *D. melanogaster* reference (30) was used for calculating the distance of the premature stop codon from the actual stop codon. To provide a null distribution of this statistic, we also estimated the PBS for variants in short introns that we identified in the per-site F_{ST} analyses. Allele frequencies for stop codons in the three geographic regions (227 samples from North America, 132 samples from Europe and North Africa and 317 samples from Southern Africa) with the largest number of published genomes out of the 1,039 available in the *Drosophila* Genome Nexus were calculated. Only polymorphic sites where the premature stop codon or variant in short introns that had a minor allele frequency greater than 5% and were typed >40% of samples in each region were used for subsequent analyses. Pairwise F_{ST} for the premature stop codons and 4,433 variants in short introns in these three regions was calculated using VCFtools and we calculated the PBS using the equation from (47). PBS trees were plotted using the R package APE v5.4-1 (46).

Detecting positive selection on p.Gln254 by modelling ancestry components.*

For modelling the ancestry components with Ohana (48), we used biallelic SNPs that had a minor allele frequency greater than 5% and were typed in >40% of samples in each geographical region. We sampled one SNP every 500bp for faster processing. We removed SNPs in linkage with each other using plink2 v1.90b3.31 (49) by assessing linkage disequilibrium for every 50 SNPs and used a step size of 5 and a variance inflation factor of 2. We retained 87,965 variants. We performed structure inference (qpas) under k=2, 3, 4, 5 and 6 and used an epsilon of 0.0001. Then we inferred population variances and covariances (nemeco) using an epsilon of 0.0001. We converted the resulting matrix into a newick tree (convert) and used the Nemetree (https://jade-cheng.com/graphs/nemetree/) to visualize it. Finally, we produced admixture-corrected allele frequencies (qpas) and performed the selection scan (selscan) for the whole genome dataset from the three regions. We also performed a Southern African ancestry specific selection scan.

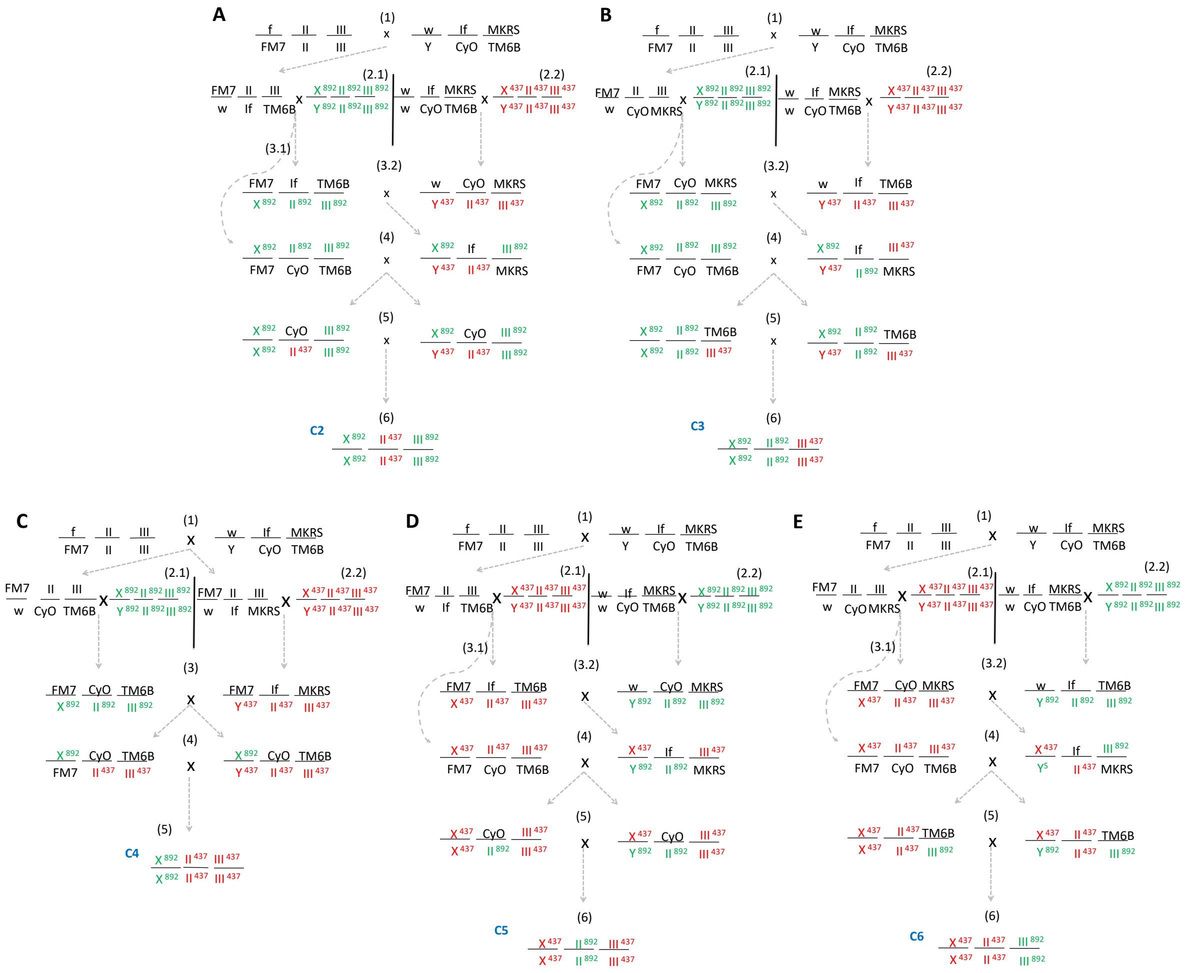

Figure S1. Crosses to generate lines with varying combinations of the 437 and 892 chromosomes. (A) C2 – X ^{892}; II ^{437}; III ^{892}. **(B)** C3 – X ^{892}; II ^{892}; III ^{437}. **(C)** C4 – X ^{892}; II ^{437}; III ^{437}. **(D)** C5 – X ^{437}; II ^{892}; III^{37}. **(E)** C6 – X ^{437}; II ^{437}; III ^{892}.

A

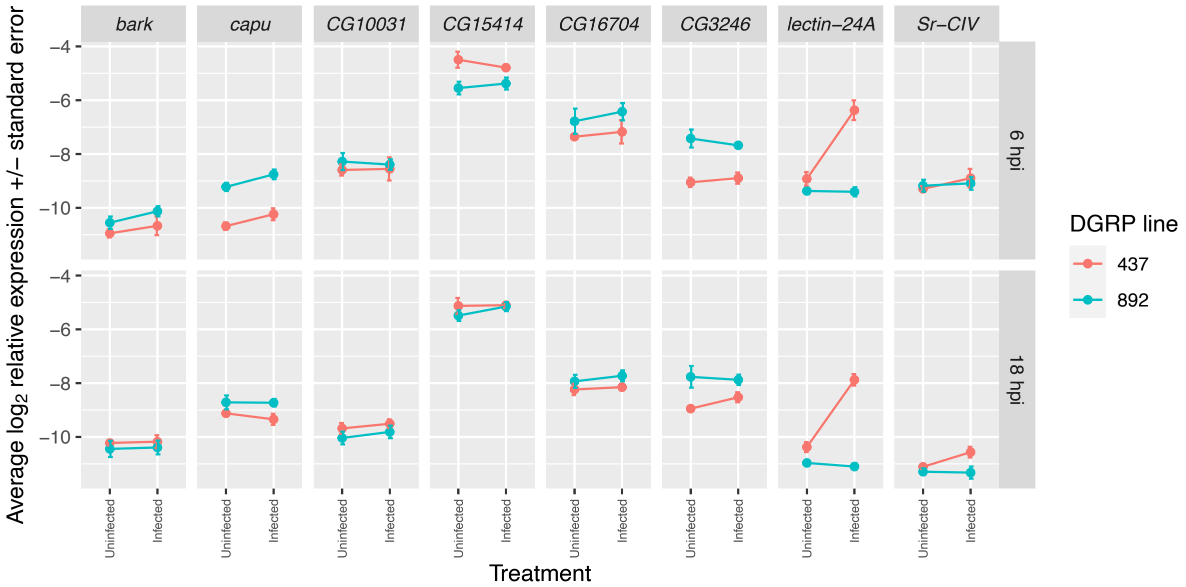

B

Gene	Hours post infection (hpi)	Degrees of freedom	F-value	P-value
bark	18 hpi	1	0.0002	0.989
bark	6 hpi	1	0.092	0.767
capu	18 hpi	1	0.319	0.582
capu	6 hpi	1	0.004	0.949
CG10031	18 hpi	1	0.015	0.906
CG10031	6 hpi	1	0.064	0.805
CG15414	18 hpi	1	0.584	0.459
CG15414	6 hpi	1	1.082	0.319
CG16704	18 hpi	1	0.093	0.766
CG16704	6 hpi	1	0.059	0.812
CG3246	18 hpi	1	1.151	0.305
CG3246	6 hpi	1	0.869	0.370
lectin-24A	**18 hpi**	**1**	**63.263**	**<0.001**
lectin-24A	**6 hpi**	**1**	**27.768**	**<0.001**
Sr-CIV	18 hpi	1	2.980	0.110
Sr-CIV	6 hpi	1	0.405	0.537

Figure S2. Candidate gene expression 6 and 18 hours post infection (hpi) with parasitoid wasps in DGRP-437 and DGRP-892. (A) Candidate gene expression was calculated relative to *RpL32*. There were four replicate measurements per gene per DGRP line per treatment. **(B)** An analysis of variance to test the effect of the interaction between treatment and DGRP line on gene expression. At a Bonferroni threshold of 0.003125, the interaction terms for *lectin-24A* at 6 hpi and 18 hpi are significant. The data for *lectin-24A* is also presented in Figure 4A.

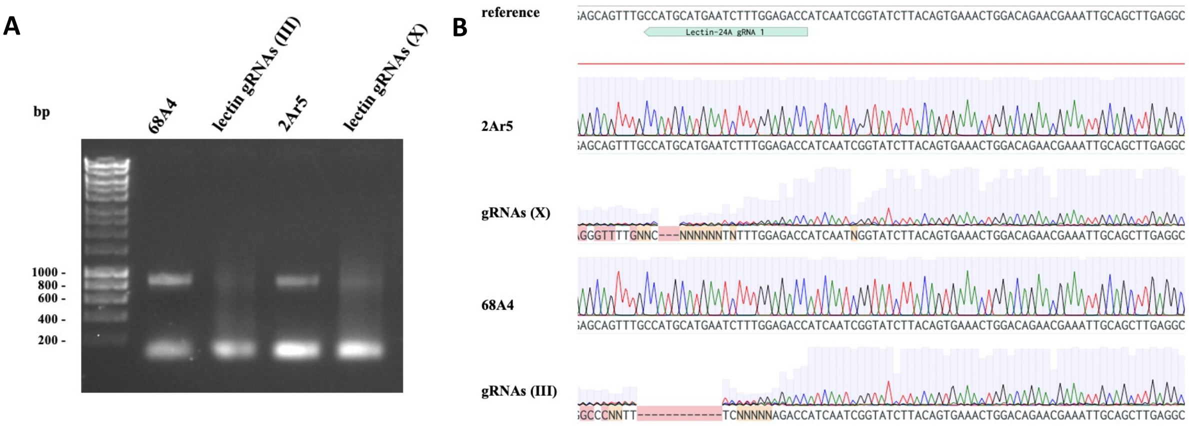

Figure S3. Somatic mutagenesis of *lectin-24A* in F_1 heterozygotes ubiquitously expressing guide RNAs targeting *lectin-24A* and Cas9 protein. (A) Gel of PCR products from amplifying the *lectin-24A* open-reading frame using pooled genomic DNA from the F_1 progeny of lines expressing guide RNAs targeting *lectin-24A* crossed to a line ubiquitously expressing Cas9 compared to control larvae. The PCR products from the control larvae show a clear band corresponding to the expected amplicon size of 845bp, whereas the PCR products from larvae with somatic knockouts of *lectin-24A* does not show a clear band at the expected size suggesting that the gene has been mutated into many smaller sizes. **(B)** Alignment of Sanger sequencing results of the PCR products shown in **(A)** using the reverse primer. The sequences in the two knockout lines become illegible from the position of the guide RNA target site, indicating successful somatic mutagenesis.

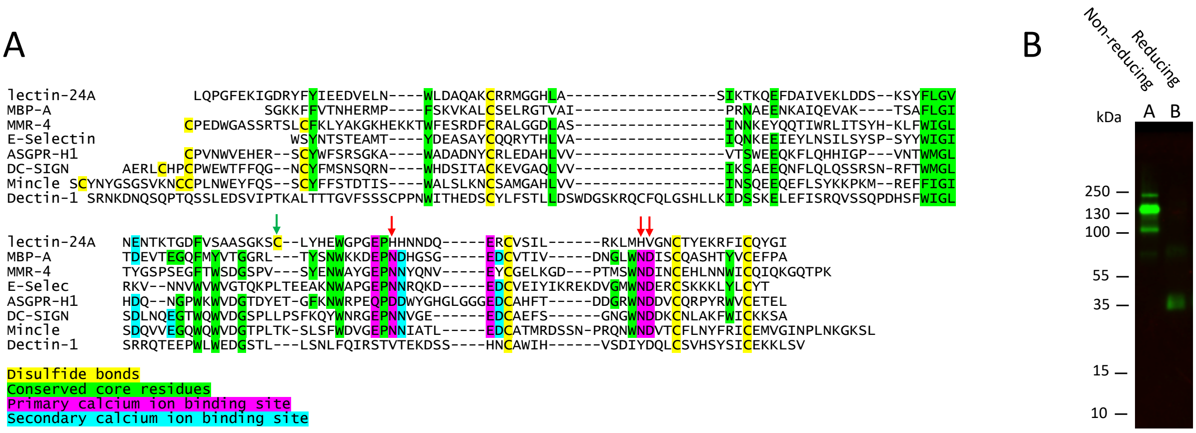

Figure S4. The carbohydrate binding domain of lectin-24A and tetramer formation. (A) Alignment of the carbohydrate recognition domain from lectin-24A and other C-type lectins. Key conserved residues that define the domain architecture are highlighted in yellow (cysteines) and green (other residues). The presence of these residues confirms that the domain will likely have a C-type lectin fold. Other colors highlight residues needed to form Ca^{2+} binding sites, including the primary Ca^{2+} binding site that forms the carbohydrate-binding site. The red arrow highlights three essential residues in the primary Ca^{2+} binding site that have been lost from lectin-24A. Note these have also been lost in Dectin-1, which has a C-type lectin fold yet binds sugar independently of Ca^{2+}. The green arrow shows an additional cysteine residue (4 additional cysteines are found outside the carbohydrate recognition domain). **(B)** lectin-24A secreted from *Drosophila* cells forms b-mercaptoethanol (2-ME) sensitive tetramers. Samples under reducing (with 2-ME) conditions exhibit the apparent 36kDa polypeptide size predicted

from translation of the open reading frame of *lectin-24A* with the FLAG and strep tags. Samples under non-reducing conditions (without 2-ME) exhibit a main polypeptide band of 140kDa.

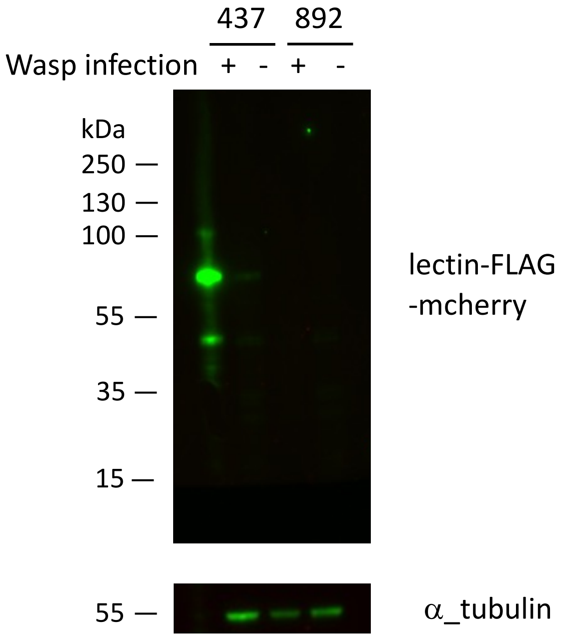

Figure S5. Transgenic FLAG-mCherry-tagged lectin-24A expressed in *Drosophila* larvae is upregulated after wasp infection. A 2.5kb promoter fragment and the open reading frame of *lectin-24A* from DGRP-437 or DGRP-892 were cloned to incorporate a FLAG and mCherry tag at the C-terminus. Transgenic flies were generated, and larvae were infected or left uninfected with *L. boulardi* G486. Protein extracts from these larvae were analyzed by Western blotting using an anti-FLAG tag antibody to detect tagged lectin-24A. Blots were stripped and re-probed with anti α-tubulin antibody to show total protein loading. Tagged DGRP-437 lectin-24A exhibits a predominant band of approximately 70kDa compared with the expected size of 60kDa. FLAG-mCherry-lectin-24A is upregulated upon infection (437+ compared to 437-).

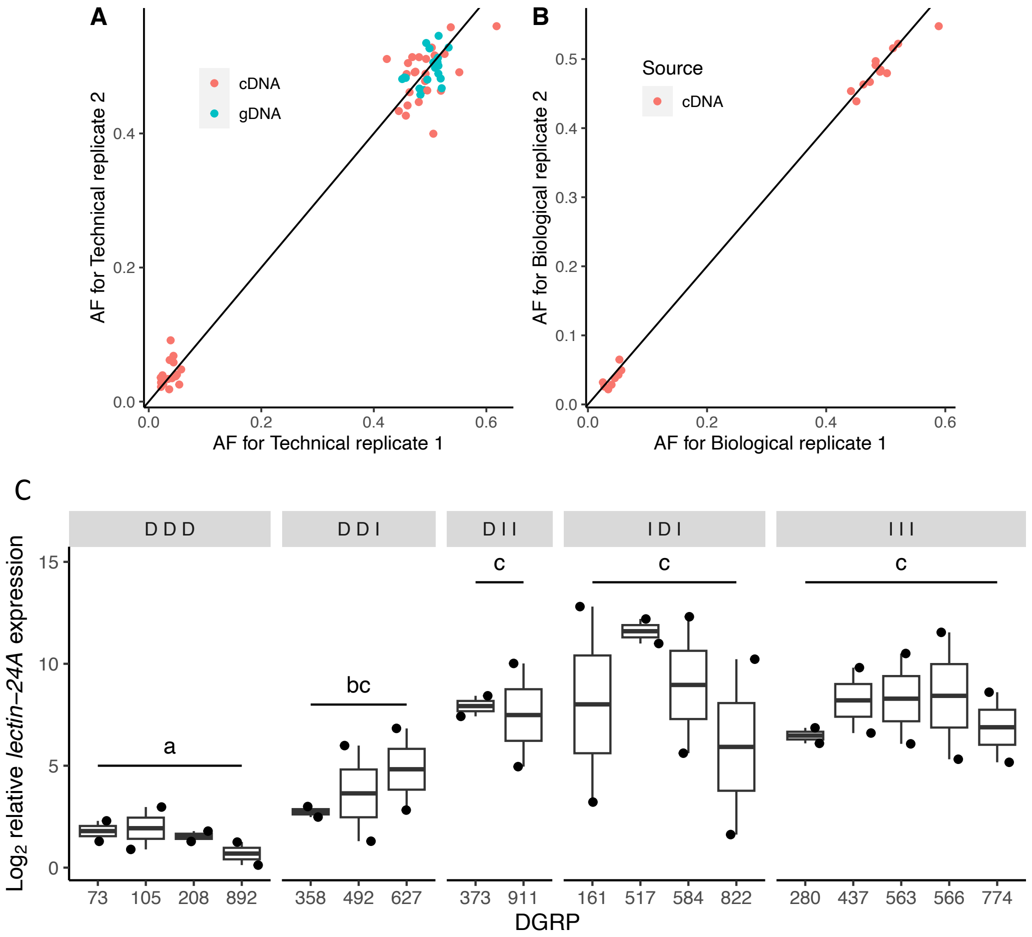

Figure S6. Genetic variation in in the expression of *Lectin 24A*. (A and B) Comparison of replicate measurements of *lectin-24A* allele-specific expression. Panel **A** compares two technical replicates on the same nucleic acid sample, and Panel **B** compares two biological replicate measurements on each inbred line. Each line was crossed to the high expression line DGRP-437, *lectin-24A* was amplified by PCR from genomic DNA (gDNA) or mRNA (cDNA), and then Illumina sequenced. The axes represent the frequency of reads derived from the test line (AF) as opposed to the DGRP-437 reference. There were no biological replicates of the genomic DNA (gDNA) controls. The read counts from the two technical replicates were summed for each biological replicate. **(C)** Expression of *lectin-24A* in inbred lines from the DGRP panel 24 hours post infection. Lines were selected to include different indel haplotypes upstream of the gene. The indel haplotype is ordered as c.-439_-433del, c.-334_-333insACATTCAT and 21bp indel (c.-171_-151del), and the deletion 'D' or insertion 'I' state for each indel is depicted. Each point is an independent biological replicate and is the mean of two biological replicates. The haplotypes were compared with Tukey's honest significant difference test, $p<0.05$ between groups, $p>0.05$ within groups. Expression is relative to the reference gene *RpL32*.

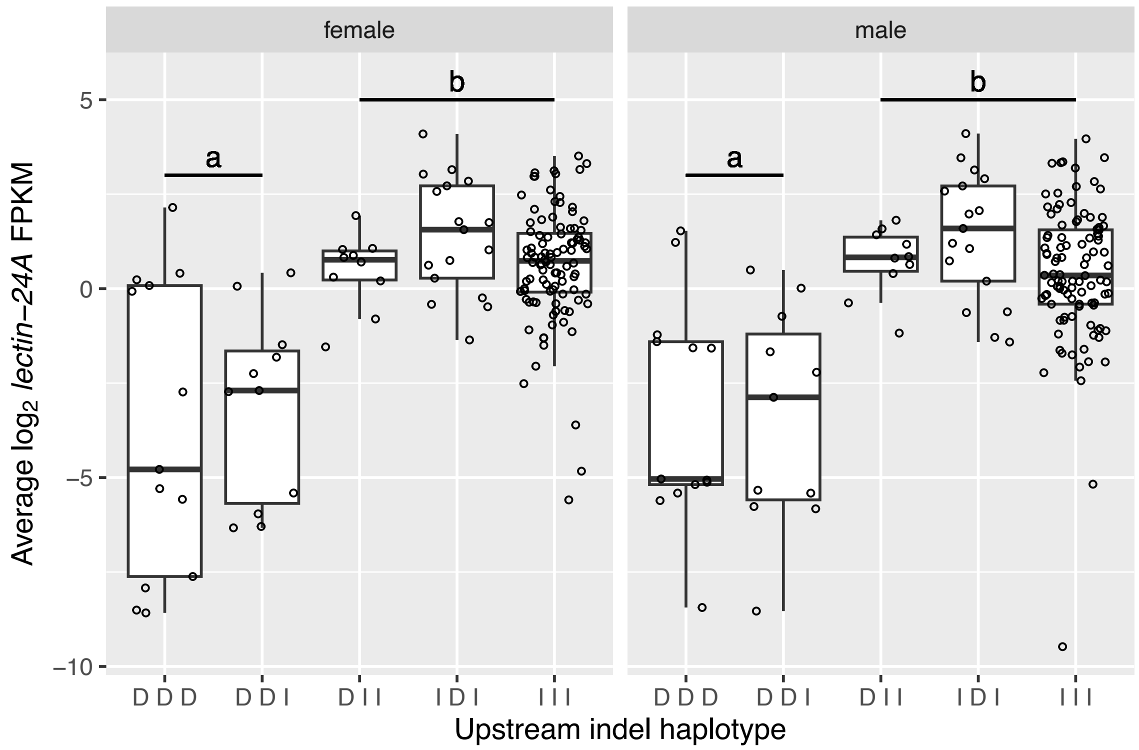

Figure S7. Adult *lectin-24A* expression (log_2FPKM) in uninfected DGRP lines (n=145). DGRP lines are grouped by their upstream *lectin-24A* indel haplotype. The indel haplotype is ordered as c.-439_-433del, c.-334_-333insACATTCAT and 21bp indel (c.-171_-151del), and the deletion 'D' or insertion 'I' state for each indel is depicted. The haplotypes were compared with Tukey's honest significant difference test, p<0.05 between groups, p>0.05 within groups.

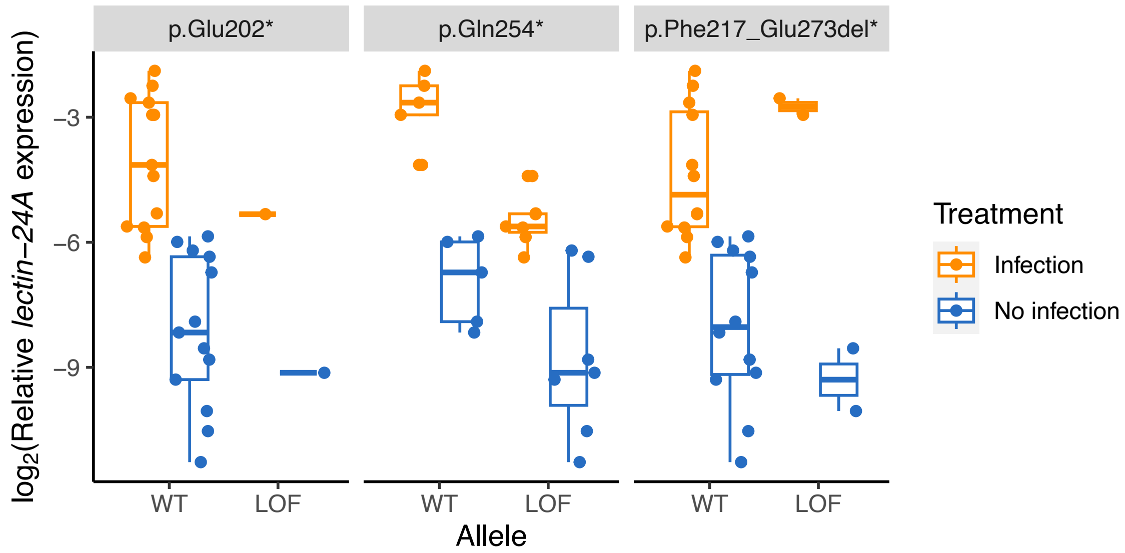

Figure S8. Expression of *lectin-24A* in Southern African lines with premature stop codons. *lectin-24A* expression is calculated relative to *RpL32*. Loss-of-function (LOF) lines carry the stop codon and wild-type (WT) lines do not. Expression was measured 24 hours post infection with parasitoid wasps.

```
DGRP-437_300bp_upstream      ATGAGTGATGTAGAAAGCCTTTTCAAATTTCATATTTAAAGAATTATTTAATAAATTCCT
Drosophila_melanogaster      ATGAGTGATGTAGAAAGCCTTTTCAAATTTCATATTTAAAGAATTATTTAAAAAATTACT
Drosophila_mauritiana        ------------------------------------------------------------
Drosophila_sechellia         ------------------------------------------------------------
Drosophila_simulans          ------------------------------------------------------------

DGRP-437_300bp_upstream      TTTAATTCGTTTTGATAATATTTAAATATTTCACAATATTGTATACATAGCAACTATTTT
Drosophila_melanogaster      TTTAATTCGTTTTGATAATATTTAAGTAATTCACAATATTGTATACATAGCAACTATTTT
Drosophila_mauritiana        ------------------------------------------------------------
Drosophila_sechellia         ------------------------------------------------------------
Drosophila_simulans          ------------------------------------------------------------

DGRP-437_300bp_upstream      TTACTGAATAAATAAGGCTATCTGGGATCAAGTTTTTTAAGTAGAAACACAAGACTGAAG
Drosophila_melanogaster      TTACTGAAT---------------------AGTTTTTTAAGTAGAAACACAAGACTGAAG
Drosophila_mauritiana        ------AATAAGTAAGGCTATCTGGGATCAAGTTTTTTAAATAGAATTACAAGACTGAAG
Drosophila_sechellia         ------AATAAGTAAGGCTATCTGGGATCAAGTTTTTTAAATAGAATTACAAGACTGAAG
Drosophila_simulans          ------AATAAGTAAGGCTATCTGGGATCAAGTTTTTTAAATAGAATTACAAGGCTGAAG
                                   ***                     ********** *****  ***** ******

DGRP-437_300bp_upstream      -AAAATAAAGCAATCAAGTAATTGATAAGCGCCTATAGAAACGTATAAAAGCAGAAAACT
Drosophila_melanogaster      -AAAATAAAGCAATCAAGTAATTGATAAGCGCCTATAGAAACGTATAAAAGCAGAAAACT
Drosophila_mauritiana        -AAAATAAAGCAATCAAGTAATTGATAAGCGCCTATTGAAACGTATAAAAG---------
Drosophila_sechellia         AAAAAAAAAGCAATCAAATAATTGATAAGCGCCTATTGAAACGTATAAAAGCAGGAAACT
Drosophila_simulans          -AAAATAAAGCAATCAAGTAATTGATAAGCGCCTATTGAAACGTATAAAAGCAGGAAACT
                              **** *********** ****************** **************

DGRP-437_300bp_upstream      CAGTCCATTTATGTAGTACTACAAAGCTGTTGATATTAAACGCATCTAAATAAATAAAAT
Drosophila_melanogaster      CAGTCCATTTATGTAGTACTACAAAGCTGTTGATATTAAACGCATCTAAATAAATAAAAT
Drosophila_mauritiana        CAGTGCATTTATGTACTACTACTAACATGGTGATATAAAACGCACCTAAA----------
Drosophila_sechellia         CAGTCCATTTATGTAGTACTACTAACATGGTGATATAAAACGCACCTAAA----------
Drosophila_simulans          CAGTCCATTTATGTAGTACTACTAACATGGTGATATAAAACGCACCTAAA----------
                             **** ********** ****** **  ** ****** ******* *****

DGRP-437_300bp_upstream      C
Drosophila_melanogaster      C
Drosophila_mauritiana        -
Drosophila_sechellia         -
Drosophila_simulans          -
```

Figure S9. MUSCLE nucleotide alignment of the 300bp region immediately upstream of the DGRP-437 start codon against three closely related *Drosophila* species. The genomes of *D.* melanogaster (v6, NCBI: AE014134.6), *D. simulans* (Prin_Dsim_3.1, NCBI: NC_052520.2), *D. sechellia* (ASM438219v1, NCBI: NC_045949.1), and *D. mauritiana* (ASM438214v1, NCBI: NC_046667.1) were used as the subject sequences for the nucleotide megaBLAST to extract the relevant regions for alignment. The sequence in yellow corresponds to c.-171_-151del.

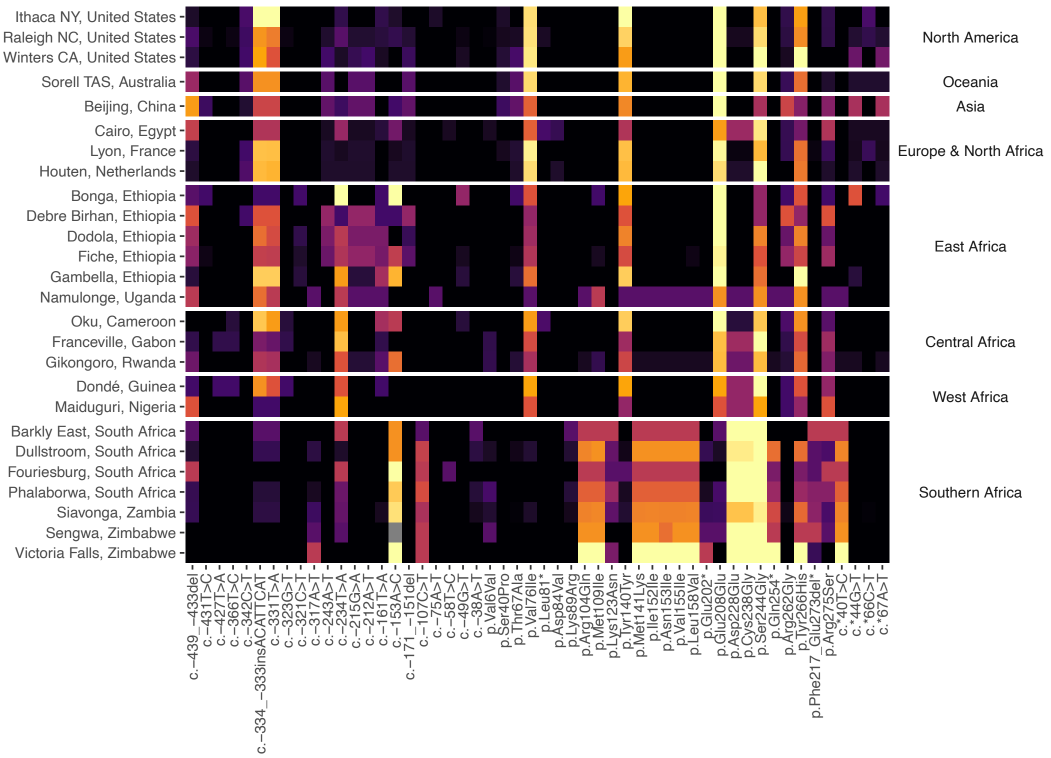

Figure S10. Geographic variation in the derived allele frequency of variants in *lectin-24A*. Includes variants found in 412bp upstream of the start codon (inclusive of three promotor indels) and 67bp downstream of the stop codon. Populations (left) are grouped into regions (right). Data is from 672 genomes in the *Drosophila* Genome Nexus that were genotyped for >50% of the *lectin-24A* gene region.

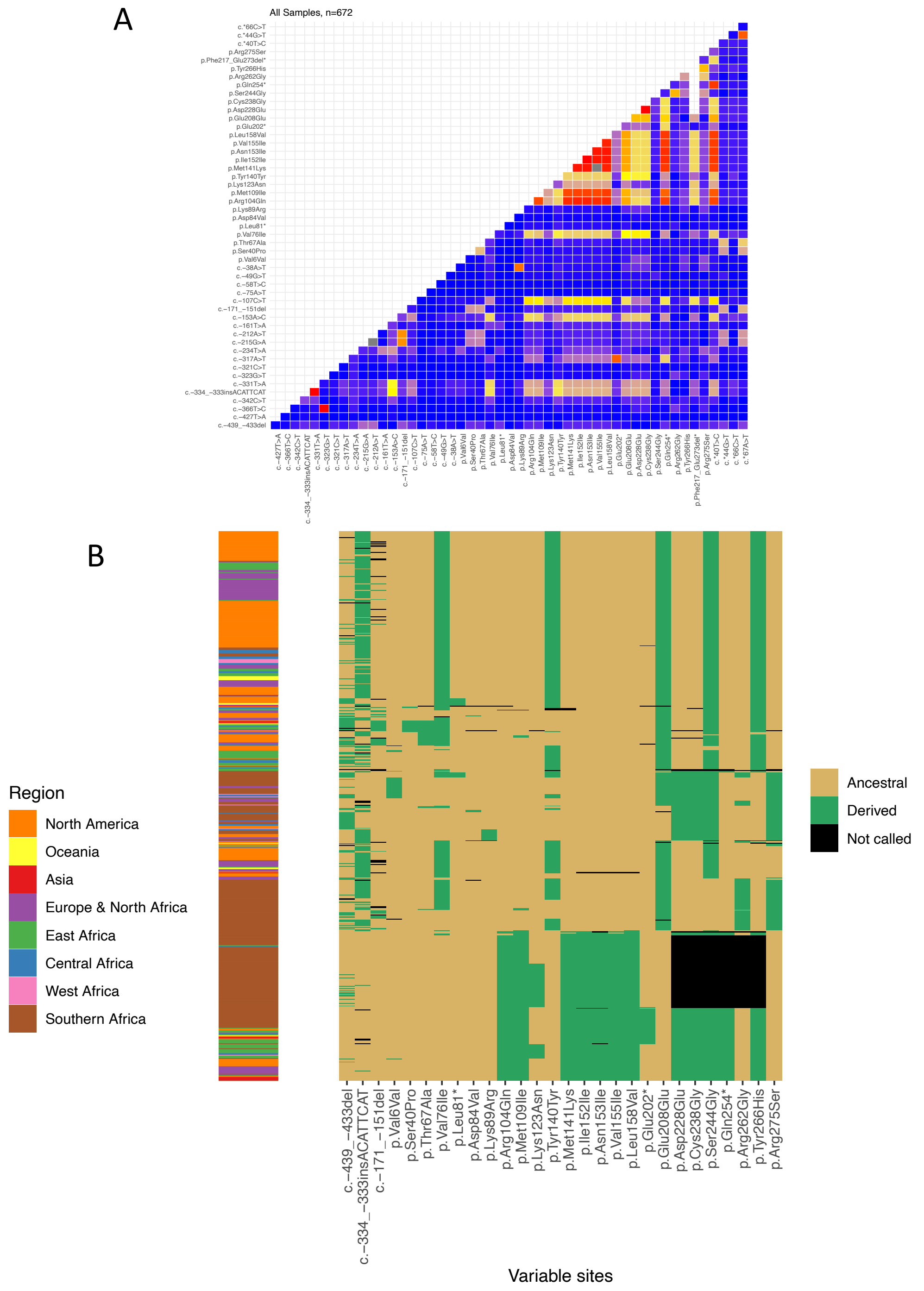

Figure S11. Haplotype Structure in *lectin-24A*. (A) Linkage disequilibrium (LD) between *lectin-24A* coding sequence and variants found in 412bp upstream of the start codon (inclusive of three promotor indels) and 67bp downstream of the stop codon. LD across all 672 samples of the *Drosophila* Genome Nexus that were genotyped for >50% of the *lectin-24A* region are shown. **(B)** Genotypes of upstream indels, nonsynonymous and premature stop codon polymorphisms in 672 *lectin-24A* sequences. Samples were clustered using a neighbor-joining tree and color-coded by the region from where they were obtained. Upstream indels were not used for tree construction but are shown in the genotype matrix. Note that samples with blocks of variants that are not called in the 3' end of the *lectin-24A* coding sequence have the 165bp deletion p.Phe217_Glu273del*. In both panels, data are from the *Drosophila* Genome Nexus.

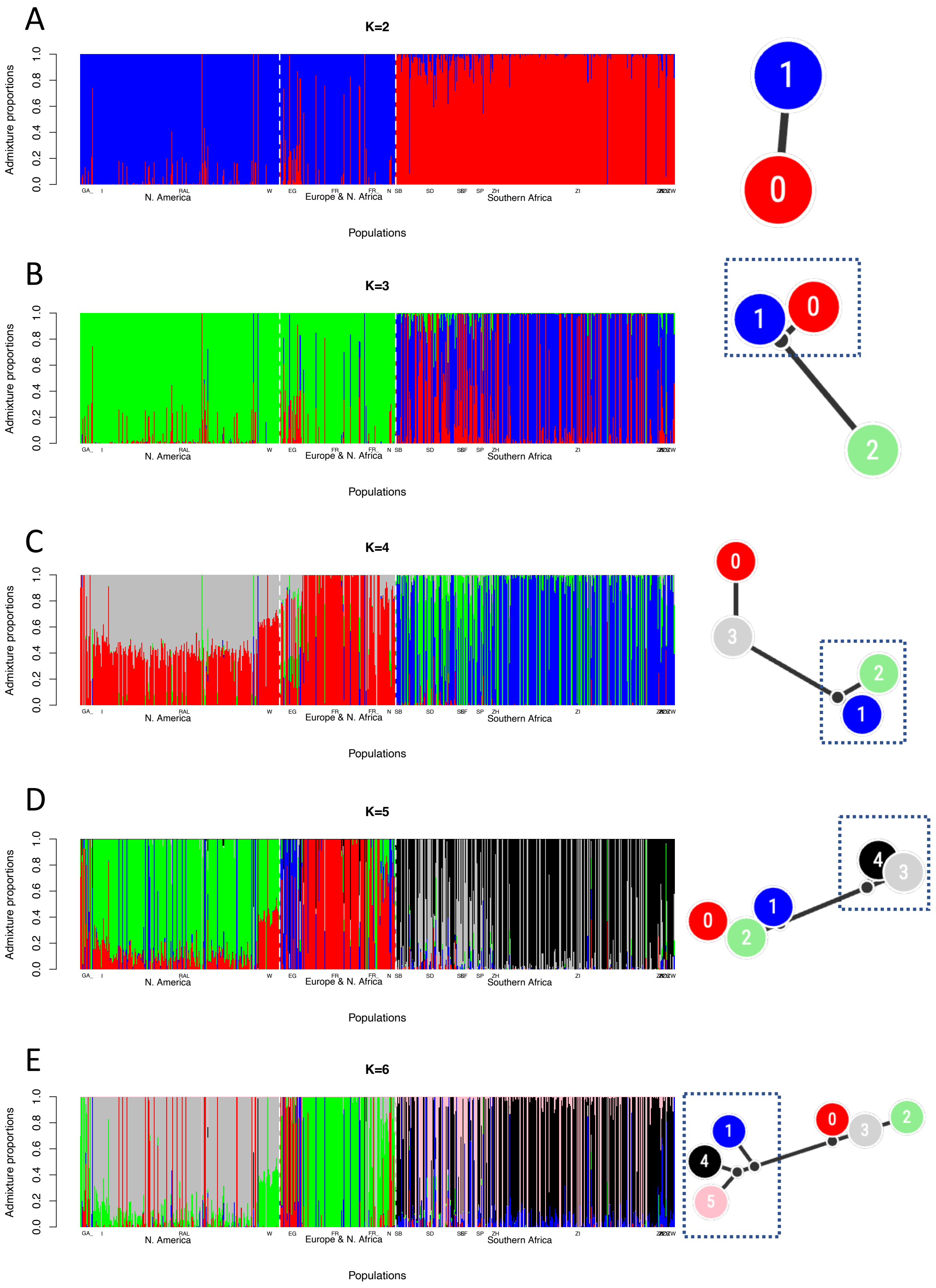

F

K	Log likelihood ratio test statistic (global)	P-value (global)	Log likelihood ratio test statistic (Southern Africa)	P-value (Southern Africa)
2	1.52	0.05	-	-
3	21.38	<0.001	22.52	<0.001
4	18.97	0.001	22.26	<0.001
5	17.06	0.008	22.36	<0.001
6	21.37	0.005	27	<0.001

Figure S12. Detecting positive selection in *Drosophila melanogaster* populations from North America (n= 317), Europe and North Africa (n=132) and Southern Africa (n= 227) by modeling ancestral admixture components. (A-E) Modeling ancestry components under K=2 to K=6. The white dotted lines in the admixture plots delineate the three regions. The dotted line boxes on the trees indicate branches leading to the African populations. **(F)** Log likelihood test statistic for p.Gln254* and associated *p*-values. *P*-values were generated by using 4,433 variants occurring within short introns to generate an empirical null distribution. The global and Southern African branch specific statistics are reported.

A

Primer	Forward	Reverse	PCR Product Size (bp)	EcoR1 Cut Size 1 (bp)	EcoR1 Cut Size 2 (bp)	Diagnostic choromsome	DGRP-437	DGRP-892	$X^{892}; II^{437}; III^{892}$	$X^{892}; II^{892}; III^{437}$	$X^{892}; II^{437}; III^{437}$	$X^{437}; II^{892}; III^{437}$	$X^{437}; II^{437}; III^{892}$
**X2**	GGATGCGGAT AGTCCACCTA	GTTAAGGGTTT GCTGGGACA	1174	451	723	X	Cut	Uncut	Uncut	Uncut	Uncut	Cut	Cut
**2L1**	GCTACCCAAA GGCAGATTCA	GTATCTTCGCG GGCTAAATG	1131	394	737	II	Uncut	Cut	Uncut	Cut	Uncut	Cut	Uncut
**3L3**	ACTATGCACA GGGGTCGTTC	CTGTGGCATTT TGGTCGAGT	1195	465	730	III	Cut	Uncut	Uncut	Cut	Cut	Cut	Uncut

B

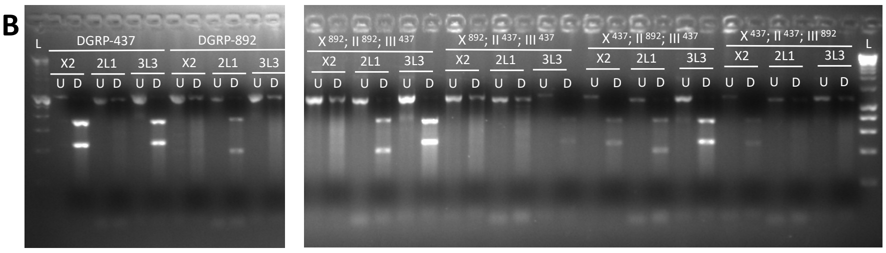

C

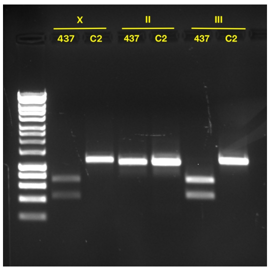

L: Bioline HyperLadder™ 1kb, U: undigested; D: EcoR1 digested

Figure S13. Genotype confirmation for chromosome substitution lines. (A) Primers designed by (1) that we used for confirming the genotypes of the chromosome substitution lines and the expected product sizes. Amplified fragments contain EcoR1 cut sites that variably cut the DGRP-437 and DGRP-892 chromosomes. **(B)** First genotype confirmation. We did not genotype X^{892}; II^{437}; III^{892} line in the first round. **(C)** Genotype confirmation of X^{892}; II^{437}; III^{892} (labelled as C2) alongside DGRP-437 as a control. Here, only digested products were run.

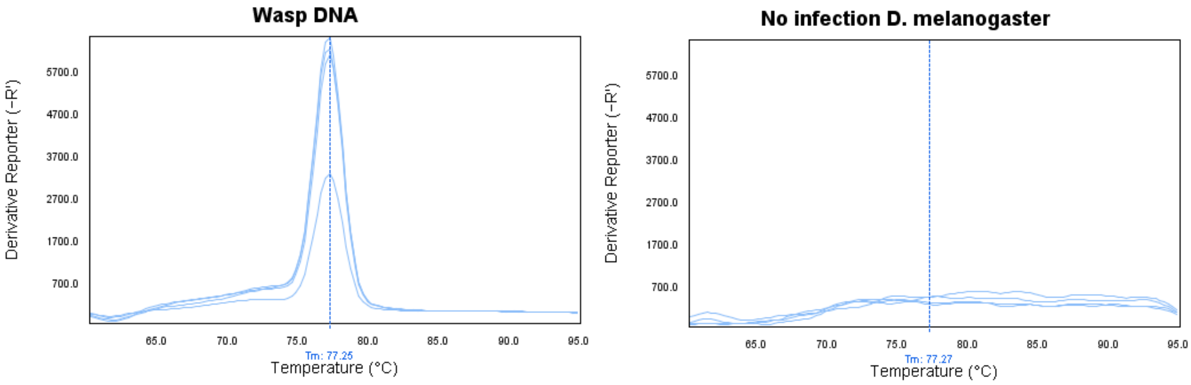

Figure S14. Amplification of parasitoid wasp DNA and DNA from uninfected DGRP-437/DGRP-892 hybrids using wasp-specific primers. Four technical replicates for each DNA were included. The peak primer melting temperature (Tm) under wasp DNA amplification is indicated by a vertical line.

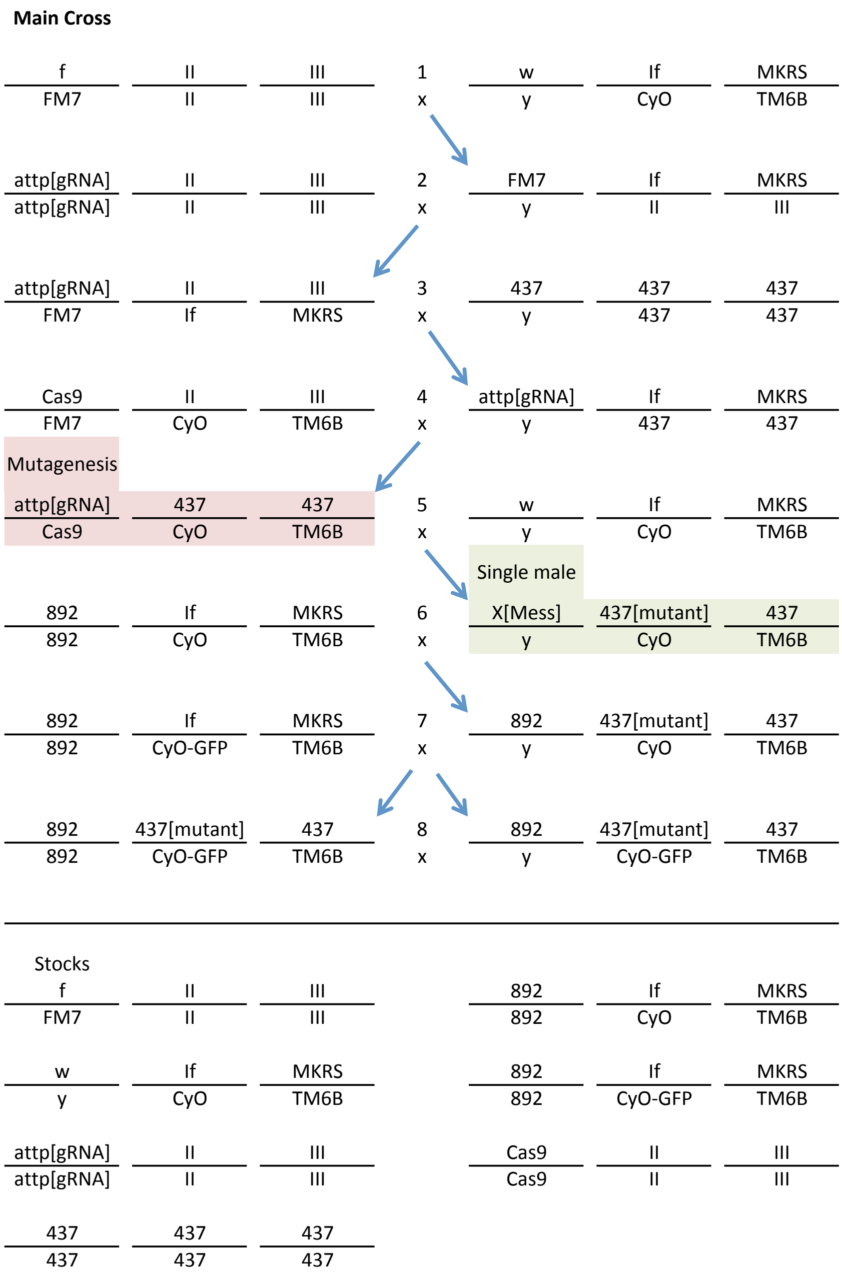

Figure S15. Crosses to generate germline mutants for *lectin-24A* in the resistant (X^{892}; II^{437}; III^{437}) background. The stock attP2Ar5 containing two guide RNAs, which were integrated at an attP site on the X chromosome, and the Cas9 containing stock (BDSC#51323) were used for mutating *lectin-24A* alleles. GFP tagged CyO was eventually removed from the mutated lines.

```
>Wild-type lectin-24A ORF
ATGTTTAGATTGTCAGTCTTAGTTCTGAACTTACTCCTTGTTAGCCATGAATTCTCGGCAGGAACCGCGAAAATTGAAAT
TCAGCCATTACCTGCCCTATGCAACGGATACTGCTTCTCCACCCTGAAG----CCAGTTATGGAGTATGTTGCTATCCAC
CAGGACAAATGGAATACTTGTACGGAAATATTAGCGAACGAAACCCGCAAGGATCAGATCCAGTTGAATATCCAGCTGGA
TGCCTTGAAAGCAGACGTTTCCAACATAAAGGCATCGCAGCTGTCAAAGGATGAGAAGCTGGACAGGATGGAGCGAGAGC
AGTTTGCCATGCATGAATCTTTGGAGACCATCAATCGGTATCTTACAGTGAAACTGGACAGAACGAAATTGCAGCTTGAG
GCGATCAAAAACACAATGGATTACATGAAGGCCCAAATGGATGGCTATTTCTCAGCCATAAATGGAGTTCAATGCCTACA
GCCAGGATTTGA-GAAGATAGGCGATAGATATTTTTACATCGAAGAAGATGTTGAGCTAAATTGGCTGGATGCTCAGGCC
AAATGTCGTCGAATGGGAGGTCACCTAGCCTCTATAAAAACTAAACAGGAGTTTGACGCAATCGTAGAGAAACTCGATGA
TTCGAAATCATACTTCCTGGGCGTCAACGAAAACACAAAAACCGGTGACTTTGTGTCTGCAGCCTCCGGAAAAAGTTGTT
TGTATCACGAGTGGGGTCCTGGTGAACCCCATCACAATAATGACCAAGAGCGATGCGTTTCAATCTTGCGAAAACTCATG
CATGTGGGCAATTGTACTTATGAAAAAAGATTCATATGCCAGTATGGCATC
>Mutant lectin-24A
-----------------------------------------------TGNATTCTCGGCAGGAACCGCGAAAATTGAAAT
TCAGCCATTACCTGCCCTATGCAACGGATACTGCTTCTCCACCCTGAAGTTATCCAGTTATGGAGTATGTTGCTATCCAC
CAGGACAAATGGAATACTTGTACGGAAATATTAGCGAACGAAACCCGCAAGGATCAGATCCAGTTGAATATCCAGCTGGA
TGCCTTGAAAGCAGACGTTTCCAACATAAAGGCATCGCAGCTGTCAAAGGATGAGAAGCTGGACAGGATGGAGCGAGAGC
AGTTT—CATGC--CAATCTTTGGAGACCATCAATCGGTATCTTACAGTGAAACTGGACAGAACGAAATTGCAGCTTGAG
GCGATCAAAAACACAATGGATTACATGAAGGCCCAAATGGATGGCTATTTCTCAGCCATAAATGGAGTTCAATGCCTACA
GCCAGGATTTNAAAAAAAATAGGCAATNAAAATTTTTANNTCCAAAAAAAAAGTTTANCTAAATTGGCTGGATGNTCAGGCA
AAA-----------------------------------------------------------------------------
```

Figure S16. Frameshift mutations introduced in the resistant *lectin-24A* allele. A 4bp insertion and a 2bp deletion (indicated in yellow), which were 129bp and 328bp downstream of the start codon, respectively, were introduced via Cas9 mediated mutagenesis. Their locations correspond approximately to the target region of the guide RNAs. The first 4bp insertion results in a frameshift and causes a premature stop codon (indicated in red) that results in a truncated 97 amino acid protein missing the entire carbohydrate binding domain.

Table S1. Table of 107 genes under the chromosome II quantitative trait locus between 10 - 11.6 cM (3.43-4.03 Mbp). Gene descriptions were obtained from Flybase (FB2019_02) (9). Cell in orange are non-coding RNAs. Cells in yellow indicate genes occurring within marker positions flanking the peak at 10.3 cM.

Pos start	Pos end	ID	Name	Alias
3404460	3426719	FBgn0031530	Pgant2	Polypeptide N-Acetylgalactosaminyltransferase 2
3445992	3447589	FBgn0031533	CG2772	FBgn0063009,BcDNA:RE45077
3449683	3452106	FBgn0031534	Snx1	Sorting nexin 1
3452466	3453755	FBgn0031535	CG12795	SO:0000010,SO:0000087,GO:0008270
3453697	3457064	FBgn0031536	Cog3	Cog3
3457302	3460367	FBgn0266670	Sec5	Secretory 5
3460322	3461952	FBgn0031538	CG3246	SO:0000010,SO:0000087,GO:0008289
3463290	3463400	FBgn0263526	mir-4971	mir-4971 stem loop
3462218	3466112	FBgn0005616	msl-2	male-specific lethal 2
3466157	3467334	FBgn0021967	ND-PDSW	NADH dehydrogenase (ubiquinone) PDSW subunit
3467342	3470004	FBgn0031540	Pif1	Pif1 ortholog
3470182	3472364	FBgn0051776	CG31776	FBgn0031541,FBgn0063621,CG8845a,CG8845,dppGalNAcT10
3472603	3475338	FBgn0051956	Pgant4	Polypeptide N-Acetylgalactosaminyltransferase 4
3475365	3476058	FBgn0266912	lncRNA:CR45372	long non-coding RNA:CR45372
3476343	3477648	FBgn0264549	lncRNA:CR43928	long non-coding RNA:CR43928
3478434	3479612	FBgn0261560	Thor	Thor
3480755	3491071	FBgn0031542	CG15414	SO:0000010,SO:0000087
3504032	3504182	FBgn0286737	snoRNA:Or-CD16	snoRNA:Orphan CD box 16
3493986	3508119	FBgn0014396	tim	timeless
3508598	3509036	FBgn0266837	lncRNA:CR45299	long non-coding RNA:CR45299
3509099	3509440	FBgn0266838	asRNA:CR45300	antisense RNA:CR45300
3509058	3510102	FBgn0051954	CG31954	FBgn0031543,SP107,CG3229
3510328	3514656	FBgn0284253	LeuRS	Leucyl-tRNA synthetase
3514872	3517264	FBgn0031544	CG17593	SO:0000010,SO:0000087,GO:0005737,GO:0012505,GO:0005509,GO:0005783
3517273	3519651	FBgn0031545	CG3213	bs30c03.y1
3519677	3522555	FBgn0031546	CG8851	SO:0000010,SO:0000087
3522521	3524056	FBgn0031547	Sr-CIV	Scavenger receptor class C%2C type IV
3525131	3527588	FBgn0031548	CG8852	CT9284
3527387	3530317	FBgn0031549	Spindly	Spindly
3530789	3532678	FBgn0031550	IFT57	Intraflagellar transport 57
3537844	3538333	FBgn0265367	lncRNA:CR44308	long non-coding RNA:CR44308
3539247	3548423	FBgn0024244	drm	drumstick
3562038	3563118	FBgn0265368	lncRNA:CR44309	long non-coding RNA:CR44309
3563308	3563540	FBgn0265960	lncRNA:CR44747	long non-coding RNA:CR44747
3575350	3575806	FBgn0264370	lncRNA:CR43822	long non-coding RNA:CR43822
3576210	3576623	FBgn0264363	CG43815	SO:0000010,SO:0000087,GO:0008150,GO:0003674,GO:0005575
3578013	3580769	FBgn0004892	sob	sister of odd and bowl

3587068	3587469	FBgn0284410	lncRNA:TS2	long noncoding RNA: testis-specific 2
3588485	3590301	FBgn0264371	lncRNA:CR4 3823	long non-coding RNA:CR43823
3604224	3606756	FBgn0002985	odd	odd skipped
3616296	3617619	FBgn0265369	lncRNA:CR4 4310	long non-coding RNA:CR44310
3619097	3621573	FBgn0015663	Ugt36A1	UDP-glycosyltransferase family 36 member A1
3621110	3621704	FBgn0031554	CG15418	SO:0000010,SO:0000087,GO:0005615,GO:0004867
3636967	3637073	FBgn0263523	mir-4972	mir-4972 stem loop
3622074	3656953	FBgn0000721	for	foraging
3662745	3692114	FBgn0085369	Drgx	Dorsal root ganglia homeobox
3667977	3668551	FBgn0259951	Sfp24Ba	Seminal fluid protein 24Ba
3668691	3669134	FBgn0259953	Sfp24Bd	Seminal fluid protein 24Bd
3669261	3669775	FBgn0259952	Sfp24Bb	Seminal fluid protein 24Bb
3670131	3670581	FBgn0261054	Sfp24Bc	Seminal fluid protein 24Bc
3692123	3692587	FBgn0031558	CG16704	FBgn0047025,CT37177,BcDNA:RH31195
3692699	3693182	FBgn0031559	CG3513	SO:0000010,SO:0000087,GO:0004867
3693184	3693905	FBgn0051779	Acp24A4	Accessory gland protein 24A4
3694105	3694418	FBgn0263096	CR43364	SO:0000087,SO:0000042
3694431	3695020	FBgn0262721	CG43165	SO:0000010,SO:0000087,GO:0005615,GO:0004867
3695032	3695411	FBgn0031560	CG16713	CT37195
3696218	3696658	FBgn0031561	IM33	Immune induced molecule 33
3697242	3697872	FBgn0031562	CG3604	FBgn0063121,BcDNA:LP04037
3698451	3699092	FBgn0031563	CG10031	SO:0000010,SO:0000087,GO:0008150,GO:0004867,GO:00056 15
3699464	3699826	FBgn0259954	CG42464	SO:0000010,SO:0000087,GO:0004867
3700195	3700658	FBgn0259955	CG42465	SO:0000010,SO:0000087,GO:0004867
3700674	3701092	FBgn0259956	Sfp24C1	Seminal fluid protein 24C1
3701290	3701699	FBgn0259957	CG42467	SO:0000010,SO:0000087,GO:0004867
3701826	3702248	FBgn0262621	CG43145	lincRNA.26
3702614	3702947	FBgn0265370	lncRNA:CR4 4311	long non-coding RNA:CR44311
3703659	3704253	FBgn0031564	CG2816	SO:0000010,SO:0000087,GO:0004867
3704106	3705488	FBgn0051778	CG31778	FBgn0031565,FBgn0047241,FBgn0063619,BcDNA:HL03084
3706551	3707063	FBgn0051777	CG31777	FBgn0031565,FBgn0063620,BEST:RH19272
3707230	3711748	FBgn0015376	cutlet	cutlet
3711881	3713105	FBgn0051955	CG31955	FBgn0031565
3713360	3716810	FBgn0031566	CG2818	SO:0000010,SO:0000087,GO:2001070,GO:0046475,GO:00473 89
3716800	3717773	FBgn0040104	lectin-24A	lectin-24A
3718185	3729145	FBgn0003386	Shaw	Shaker cognate w
3730466	3754617	FBgn0031568	CG10019	FBgn0043805,GH25970,BEST:GH25970
3757256	3769525	FBgn0263846	CG43707	CG31959,FBgn0051959,FBgn0031569,CG31772,FBgn005177 2,CG31772,CG31959,CG10020
3765494	3765863	FBgn0263842	CG43703	SO:0000010,SO:0000087,GO:0004867
3767621	3767688	FBgn0262392	mir-1004	mir-1004 stem loop
3769926	3770454	FBgn0263843	lncRNA:CR4 3704	long non-coding RNA:CR43704
3771700	3784121	FBgn0004893	bowl	brother of odd with entrails limited
3784947	3785622	FBgn0051960	CG31960	FBgn0031570,CG10022,CBP1

3785738	3786343	FBgn0051958	CR31958	FBgn0031570,CG10022,CG31958
3787167	3802726	FBgn0031571	bark	bark beetle
3803545	3810671	FBgn0021800	Reph	Regulator of eph expression
3810801	3813410	FBgn0031573	CG3407	cg3407
3815438	3815948	FBgn0264885	lncRNA:CR44076	long non-coding RNA:CR44076
3817724	3818534	FBgn0264886	lncRNA:CR44077	long non-coding RNA:CR44077
3825675	3827099	FBgn0003430	slp1	sloppy paired 1
3836840	3839185	FBgn0004567	slp2	sloppy paired 2
3845130	3845716	FBgn0266318	lncRNA:CR44983	long non-coding RNA:CR44983
3851951	3852604	FBgn0267007	lncRNA:CR45451	long non-coding RNA:CR45451
3854288	3854994	FBgn0267005	lncRNA:CR45449	long non-coding RNA:CR45449
3856478	3857172	FBgn0267006	lncRNA:CR45450	long non-coding RNA:CR45450
3861405	3862097	FBgn0264887	lncRNA:CR44078	long non-coding RNA:CR44078
3862668	3867507	FBgn0031574	TTLL4B	Tubulin tyrosine ligase-like 4B
3867697	3871425	FBgn0031575	Cep97	Centrosomal protein 97kDa
3871271	3872458	FBgn0051957	CG31957	SO:0000010,SO:0000087,GO:0006413,GO:0003743
3872646	3902860	FBgn0000256	capu	cappuccino
3911796	3914288	FBgn0051773	CG31773	FBgn0031577,CG3390
3902928	3991713	FBgn0051774	fred	friend of echinoid
4000018	4000806	FBgn0264341	CG43797	SO:0000010,SO:0000087
4001084	4001620	FBgn0266883	lncRNA:CR45344	long non-coding RNA:CR45344
4004080	4004480	FBgn0085205	CG34176	SO:0000010
4004984	4005670	FBgn0031579	CG15422	SO:0000010,SO:0000087
4006041	4006606	FBgn0031580	CG15423	SO:0000010,SO:0000087
4007690	4007984	FBgn0264295	CG43773	FBgn0031581,CG10039
4008653	4009090	FBgn0264296	CG43774	FBgn0031581,CG10039
4031377	4115749	FBgn0000547	ed	echinoid

Table S2. Sanger sequencing calls for the three upstream promoter indels in 171 DGRP lines. The indels were sequenced in the order: c.-439_-433del, c.-334_-333insACATTCAT and 21bp indel (c.-171_-151del). When a heterozygous indel was encountered, indicating that the line was not fully inbred, the remaining indels were not sequenced and, therefore, indicated with NA. I: insertion allele, D: deletion allele and H: heterozygous. The c.-334_-333insACATTCAT call from DGRP Freeze2.0 (1, 2) and a SNP that occurs within the 21bp insertion are also indicated.

Line	c.-439_-433del	c.-334_-333insACATTCAT	c.-171_-151del	Snp at position 3 in c.-171_-151del
21	I	I	NA	NA
26	D	D	NA	NA
31	I	I	NA	NA
32	I	D	NA	NA
38	I	I	I	G
40	I	I	I	A
41	I	I	NA	NA
42	I	I	I	G
45	I	I	I	A
48	I	D	I	G
57	I	I	I	G
59	I	I	I	G
69	D	D	D	NA
73	D	D	D	NA
75	I	I	I	G
85	I	H	NA	NA
88	H	NA	NA	NA
91	I	I	I	G
93	I	D	I	G
100	H	NA	NA	NA
101	I	H	NA	NA
105	D	D	D	NA
109	I	I	I	G
129	I	I	I	A
136	I	I	I	G
138	D	D	D	NA
142	I	I	I	G
153	I	I	I	G
161	I	D	I	G
176	I	I	I	A
177	I	I	I	G
181	D	D	D	NA
189	I	I	I	A
195	I	I	I	A

208	D	D	D	NA
217	I	I	I	A
228	D	D	D	NA
229	I	I	I	A
235	I	I	I	A
237	I	H	NA	NA
256	I	I	I	G
280	I	I	I	G
287	I	I	I	G
301	H	NA	NA	NA
303	I	I	I	G
304	I	I	I	G
306	I	I	I	A
309	I	I	I	G
313	I	I	I	A
317	I	I	I	G
318	I	I	I	G
319	I	I	I	A
320	I	I	I	A
321	I	I	I	A
324	I	I	I	G
332	I	I	I	A
336	I	I	I	G
338	I	H	NA	NA
340	I	I	I	G
348	D	D	NA	NA
350	D	D	I	G
352	I	H	NA	NA
354	I	D	I	G
355	I	D	I	G
356	I	I	I	A
357	I	I	I	A
358	D	D	I	G
360	I	I	I	A
361	I	I	I	A
362	I	D	I	G
365	I	I	I	G
370	I	I	I	A
371	I	I	I	A
373	D	I	I	G
374	I	I	I	G
375	D	D	D	NA

377	I	H	NA	NA
379	I	I	I	G
380	I	I	I	G
381	NA	D	I	G
382	I	I	I	G
385	I	I	I	A
386	D	D	I	G
390	I	D	I	G
391	I	I	I	G
392	I	I	I	A
395	I	I	I	A
397	I	I	I	A
399	I	I	I	A
405	I	H	NA	NA
406	D	D	I	G
409	D	I	I	G
426	H	NA	NA	NA
427	I	I	I	A
437	I	I	I	A
439	I	I	I	G
440	I	I	I	G
441	I	I	I	G
443	D	D	I	G
461	D	I	I	G
486	D	I	I	G
491	I	I	I	G
492	D	D	I	G
502	I	H	I	G
508	I	I	I	G
509	D	I	I	A
517	I	D	I	G
528	I	H	NA	NA
530	I	I	I	G
531	I	I	I	G
535	I	I	I	A
551	I	I	I	G
555	I	I	I	G
559	D	D	D	NA
563	I	I	I	A
566	I	I	I	A
584	I	D	I	G
589	I	I	I	G

595	I	D	I	G
596	I	I	I	A
627	D	D	I	G
703	I	I	I	A
705	I	I	I	G
712	I	D	I	G
714	D	I	I	G
716	I	I	I	G
721	I	I	I	G
732	I	I	I	G
737	I	I	I	G
738	I	I	I	G
748	D	D	I	G
757	I	I	I	G
774	I	I	I	G
776	I	I	I	G
783	I	I	I	G
786	I	I	I	G
787	I	I	I	A
790	I	I	I	G
796	I	D	I	G
802	I	I	I	A
804	I	I	I	G
808	I	I	I	G
810	I	I	I	G
812	I	H	NA	NA
818	I	I	I	A
819	I	I	I	A
820	D	I	I	G
821	I	I	I	A
822	I	D	I	G
832	I	D	I	G
843	I	I	I	A
849	I	I	I	A
850	I	I	I	A
852	I	I	I	A
853	I	I	I	A
855	I	I	I	G
857	I	I	I	G
859	I	I	I	A
861	I	I	I	A
879	I	I	I	A

882	I	I	I	A
884	I	I	I	G
890	I	I	I	A
892	D	D	D	NA
894	I	D	I	G
897	I	I	I	G
900	I	I	I	G
907	I	I	I	G
908	I	I	I	A
911	D	I	I	G
913	I	I	I	G

Table S3. Association of reference and alternate alleles for biallelic variants present in 1,000bp upstream of the *lectin-24A* start codon with capability to induce expression following parasitoid infection. Sixteen DGRP lines which all carried the insertion allele for the 21bp indel expression were studied. The alleles were classified as to whether they were induced after infection based on allele-specific expression data.

Variant	DGRP lines containing reference allele		DGRP lines containing alternate allele	
	High expression haplotype count	Low expression haplotype count	High expression haplotype count	Low expression haplotype count
3717777 _SNP	11	4	1	0
3717785 _SNP	12	4	0	0
3717811 _SNP	12	4	0	0
3717881 _SNP	6	0	2	4
3717889 _SNP	11	4	0	0
3717919 _SNP	0	0	11	4
3717922 _SNP	0	0	11	4
3717941 _SNP	7	0	4	2
3717950 _SNP	0	0	11	3
3717956 _SNP	0	0	12	3
3718030 _SNP	12	4	0	0
3718036 _SNP	3	4	8	0
3718037 _SNP	3	4	6	0
3718049 _SNP	12	4	0	0
3718073 _SNP	12	4	0	0

3718137_SNP	11	4	1	0
3718144_SNP	11	4	1	0
3718199_SNP	12	4	0	0
3718222_SNP	12	4	0	0
3718266_SNP	12	4	0	0
3718287_SNP	12	4	0	0
3718317_SNP	12	0	0	4
3718337_SNP	12	4	0	0
3718340_SNP	11	4	1	0
3718354_SNP	12	0	0	3
3718388_SNP	12	0	0	3
3718452_SNP	12	0	0	4
3718506_SNP	12	4	0	0
3718528_SNP	11	4	1	0
3718554_SNP	12	4	0	0
3718615_SNP	12	4	0	0
3718624_SNP	9	4	2	0
3718649_SNP	12	4	0	0
3718679_DEL	12	4	0	0
3718701_SNP	12	4	0	0
3718712_SNP	12	4	0	0
3718750_SNP	12	4	0	0

Table S4. Type 1 ANOVA tests where the four coding sequence variants are the second predictor for melanization rates. The expression haplotype is the first predictor. *P*-values were adjusted using a false discovery rate correction.

**Variant id**	**Chromosome**	**Position**	***F*-value**	**Df**	***P*-value**	**Adjusted *P*-value**
p.Lys209Lys	2L	3717102	2.36375286	1	0.12461643	0.49846571
p.Tyr140Tyr	2L	3717309	0.61894591	1	0.43168877	0.61985499
p.Thr67Ala	2L	3717530	0.13545543	1	0.71294229	0.71294229
p.Ser40Pro	2L	3717611	0.53462588	1	0.46489124	0.61985499

Table S5. Primers to amplify indel and SNP primers for quantitative trait locus (QTL) mapping of the second chromosome of *Drosophila melanogaster*. Genetic and physical positions were obtained from version 6 of *D. melanogaster* genome from Flybase (FB2019_02) (9). As genetic positions were only identified to the closest integer on Flybase, positions in between were assigned based on their respective physical location. Indel size variation and number of SNPs differentiating DGRP-437 and DGRP-892 were based on DGRP Freeze 2.0 (1, 2).

Location (Chr-cM)	Forward primer	Reverse primer	Forward primer start (bp)	Reverse primer start (bp)	Type	437size (bp)	892 size (bp)	#SNPs
2L-3	CATTACACTTGCAGCCGGAA	GGTGTACCGAATGAGATTGCG	2007685	2008065	Indel	380	325	8
2L-5	ACACCAAATACGCCAAGCAA	AGGCTGAGGTATGCGTTCTT	2243388	2243634	Indel	247	292	0
2L-7	CGCAATCAGAGCCAGCATTT	CCAACCGGTCCAAAGTTCAG	2804176	2804735	Indel	524	523	8
2L-10.3	CGTCGATGGCAATATCTCCG	CCTATCGCCACTGTATCCCA	3522926	3523290	Indel	364	304	6
2L-12	CTGGACAGCTGGATCTCGAT	TCGCTCTGTCGCTCTGTTAT	4250761	4251130	Indel	369	416	5
2L-17	ATGGTGGAGGCGCTTAAGT	ACTCTGTCACTGTTGCTGTTG	5511730	5512284	Indel	490	545	4
2L-27	TTCCACGGTAGCAAAATGGC	CCCCGTCTCCCTCTTTCTTT	7564467	7564772	Indel	305	195	0
2L-34	AGGATTTGAGGGGAGTGCAA	GAGCAGAGGTAGAGGCAGAG	8762316	8762746	Indel	430	484	8
2L-54	AATTGCACACGGAGGACAAC	GCCAATTAGAGCTCCACTGC	19518131	19518468	Indel	416	337	3
2R-67	TTTGATGGATGAGGAGCCGT	CTGATGCAGTGTTTACCCCG	12748432	12748868	Indel	479	436	20
2R-104	ATCGACTCAAGTGGCTGTCA	GCACGATCAAGTTTCACCGA	23666654	23667009	Indel	355	406	4
2L-8	TGTCGTGGTTTTACAAGCCG	TGGAGACGCTCGATCTAACC	3056629	3057129	SNP	501	500	10
2L-8.5	GCTCTGGTTGGGTAGGGAG	AGTGCGGATGGTCAGTGTAA	3214280	3214818	SNP	538	538	6
2L-9	GCAGTATTCGCGGCATTTTG	TGAATTCGCAGCGTTGAACA	3325117	3325661	SNP	544	544	13
2L-10	TGTGGGGACGGGGATTTATT	GTAAGCGTGAGAGGGAGACA	3425610	3426126	SNP	516	516	8
2L-10.7	GCCAGGATTTGAGAAGATAGGC	TCTTCATTTCGCCACTTAGCT	3716618	3717252	SNP	634	634	5
2L-11	TCAGTACCACCGCTCCAAAT	TCGTTTCGGCAATAGTTAATGGT	3802116	3802665	SNP	549	549	1
2L-11.3	GTAAATTCCGCGACTGGGTC	AAAATGTTGGTAGGGGCAGC	3930005	3930570	SNP	565	565	17
2L-11.6	GAGGAGAGCAGCAACGACTA	CCAATTGACAGGAGCCAACA	4032729	4033264	SNP	535	535	16

Table S6. Primers used for generating pCFD5-w lectin-24A knockouts (KO) for integrating two guide RNAs targetting *lectin-24A*. Sequence in bold refereces to the insert and nonbold is the backbone vector.

Name	Sequence
Lectin24aKO_PCR1_Fw	GCGGCCCGGGTTCGATTCCCGGCCGATGCA**GGTCTCCAAAGATTCATG CA**GTTTTAGAGCTAGAAATAGCAAG
Lectin24aKO_PCR1_Rv	**GAAGCCAGTTATGGAGTATG**TGCACCAGCCGGGAATCGAACCC
Lectin24aKO_PCR2_Fw	**CATACTCCATAACTGGCTTC**GTTTTAGAGCTAGAAATAGCAAG
Lectin24aKO_PCR2_Rv	**TGCATGAATCTTTGGAGACC**TGCACCAGCCGGGAATCGAACCC
Lectin24aKO_PCR3_Fw	**GGTCTCCAAAGATTCATGCA**GTTTTAGAGCTAGAAATAGCAAG
Lectin24aKO_PCR3_Rv	ATTTTAACTTGCTATTTCTAGCTCTAAAAC**GAAGCCAGTTATGGAGTA TG**TGCACCAGCCGGGAATCGAACCC
U63seqfwd	ACGTTTTATAACTTATGCCCCTAAG
pCFDseqrev	GCACAATTGTCTAGAATGCATAC

Table S7. Primers used to generate fluorescent reporter constructs carrying different alleles for the three indels and six SNPs polymorphic between DGRP-892 and DGRP-437 occurring in the 1,000bp region upstream of *lectin-24A*.

Primer	Sequence
Lec500bppromF	GCTATACGAAGTTATCTGCAGGAATCCGAACAAACCGAACC
Lec500bppromR	CCTTGCTCACCATGATTTTATTTATTTAGATGCGTTTAATATCAACAGCTT TG
venusLecPromF	TAAAATCATGGTGAGCAAGGGCGAGGAG
venusLecPromR	ATTATAAGCTGCAATAAACAAGTTTTACTTGTACAGCTCGTCCATGCC
lecpromD21frag1R	TACTTAAAAAACTATTCAGTAAAAAATAGTTGCTATGTATACAATATTGT GAAATATT
lecpromD21frag2F	TTTTTACTGAATAGTTTTTTAAGTAGAAACACAAGACTGAAGAAAATAAA GCAATCAAG
1_2insmutfrag1R	GCACATTGGCGCCATACTCAAGTGAATGTCAGAGC
1_2insmutfrag2F	GGCGCCAATGTGCAACACTGCGTATGAGTGATG
snpmutfrag1R	CTTAAATATTATCAAAACGAATTAAAAGTAATTTTTTAAATAATTCTTTAA ATATGAAATTTG
Snpmutfrag2F	CTTTTAATTCGTTTTGATAATATTTAAGTAATTCACAATATTGTATACATA GC
lecPrdelta7Rv	CTGCGCATGCACTCGATTCTGAGATGAAAAACAAATC
LecPrdelta8Fw	ATCTCAGAATCGAGTGCATGCGCAGTGTCAC
LecPrdelta8Rv	ACATCACTCATACGCAGTGTTGCACATTGGC
LecPr21bpFw	GTGCAACACTGCGTATGAGTGATGTAGAAAGC
LecPr2snpF	CATTCATACTTGAGTATGGCGCCAATGTGC
LecPr2snpRv	TTGGCGCCATACTCAAGTATGAATGTGAATGTCAGAG
Truedelta8Fw	GATGTTGATTTGTTTTTCATCTCAGAATCGAGTG
truedelta8Rv	TTGGCGCCATACTTGAGTGAATGTCAGAGCAAAAG
21bp2snpFw	GACATTCACTCAAGTATGGCGCCAATGTGC
7bptrue8Rv	CTGAGATGAAAAACAAATCAACATCCCTCCAC

Table S8. *lectin-24A* promoter (LP) constructs carrying different alleles for the three indels and six SNPs polymorphic between DGRP-892 and DGRP-437 occurring in the 500bp region upstream of *lectin-24A*. Δ refers to the removal of the insertion allele from the DGRP-437 *lectin-24A* upstream sequence (LP437). For SNPs, DGRP-892 alleles were substituted with the DGRP-437 alleles in LP437.

Construct	Primers used	Template
LP437	Lec500bppromF/Lec500bppromR	DGRP-437
	venusLecPromF/venusLecPromR	venus
LP892	Lec500bppromF/Lec500bppromR	DGRP-892
	venusLecPromF/venusLecPromR	venus
Δc.-171_-151del	Lec500bppromF/lecpromD21frag1R	DGRP-437
	lecpromD21frag2F/lec500bppromR	DGRP-437
	venusLecPromF/venusLecPromR	venus
Δc.-439_-433del, Δ333insACATTCAT	Lec500bppromF/1_2insmutfrag1R	DGRP-892
	1_2insmutfrag2F/lec500bppromR	DGRP-437
	venusLecPromF/venusLecPromR	venus
3717919_SNP, 3717922_SNP, 3717950_SNP, 3717956_SNP	Lec500bppromF/snpmutfrag1R	DGRP-437
	Snpmutfrag2F/lec500promR	DGRP-437
	venusLecPromF/venusLecPromR	venus
Δc.-439_-433del	Lec500promF/lecPrdelta7Rv	DGRP-892
	LecPrdelta8Fw/Lec500PromR	DGRP-437
	venusLecPromF/venusLecPromR	venus
Δ333insACATTCAT, 3718036_SNP, 3718037_SNP	LecPrdelta8Fw/LecPrdelta8Rv	DGRP-892
	LecPr21bpFw/Lec500PromR	DGRP-437
	Lec500PromF/LecPrdelta7Rv	DGRP-437
	venusLecPromF/venusLecPromR	venus
3718036_SNP, 3718037_SNP	LecPr2snpF/LecProm500Rv	DGRP-437
	LecProm500Fw/LecPr2snpRv	DGRP-437
	venuslecPromF/venusLecPromR	venus
Δ333insACATTCAT	LecProm500Fw/7bpRvtrue8	DGRP-437
	Truedelta8Fw/truedelta8Rv	DGRP-892
	21bp2snpFw/LecProm500Rv	DGRP-437
	venuslecPromF/venusLecPromR	venus

Table S9. SNPs segregating in the 21 DGRP lines used for testing *lectin-24A* allele expression. Calls for eight SNPs in the amplified region between 3717069 and 3717568 on chromosome 2L were obtained from DGRP Freeze2.0 (1, 2). 2L:3717102 was the polymorphic site used for estimating allele-specific expression.

**Line**	**2L_3717 102**	**2L_3717 224**	**2L_3717 262**	**2L_3717 291**	**2L_3717 309**	**2L_3717 487**	**2L_3717 503**	**2L_3717 530**
Ref allele	C	A	T	A	A	A	T	C
Alt allele	T	T	C	G	G	T	C	T
105	0	0	0	0	0	0	0	0
136	0	0	0	0	2	0	0	2
161	0	0	0	0	2	0	0	2
208	0	0	0	0	0	0	0	0
217	0	0	0	0	2	0	0	2
228	0	0	0	0	0	0	0	0
280	0	0	0	0	2	0	0	2
350	0	0	0	0	2	2	0	2
386	0	0	0	0	2	2	0	2
406	0	0	0	0	2	2	0	2
409	0	0	0	0	2	0	0	2
427	0	0	0	0	2	0	0	2
437	2	0	0	0	2	0	0	2
486	0	0	0	0	2	0	2	2
509	0	0	0	0	0	0	0	2
517	0	2	0	0	2	0	0	2
584	0	0	0	0	2	0	0	2
627	0	0	0	0	2	2	0	2
787	0	0	0	0	2	0	0	2
820	0	0	0	0	2	0	0	2
822	0	0	2	2	2	0	2	2
892	0	0	0	0	0	0	0	0

Table S10. Primers for candidate gene qPCR.

**Gene**	**Forward primer**	**Reverse primer**
capu	ACGCGGCTCATCTTATGTACC	CCGCTGGAACTCTTCGGATT
bark	ACCATCGAACCGGGTGTTAC	ATTCTGGAGGTTGGTGTGCC
CG8852	TCAGCGAGCATTCACCACAT	TCCAGGACGGTAGATCAGCA
Sr-CIV	AGCACGGATTTGACAGAAGT	TAAGAACGATCGCCCAGAGC
CG3246	TCAAGGCGTATGGCTTGTCC	AGGCCACCGTTGAATCTCAT
CG15414	TGCAGCTAGCGGAATATACAC	TTCAGTCGGTGGTAAGGCAT
CG16704	TCAAGGGTACTTGCCGTTCC	AAGGTGAAGCACTCGTTCGT
CG10031	TGGTACCAGATCCCAAATGCC	CCGTACTTGAAGGTCTCGCA
lectin-24A	GCAGCCTCCGGAAAAAGTTG	GAAACGCATCGCTCTTGGTC

Table S11. Comparison of *lectin-24A* variant calls using the method used in this paper versus those made by DGRP Freeze2.0 (1, 2), Global Diversity Lines (36) and Sanger sequencing. Green cells indicate a match between datasets being compared and orange cells indicate a mismatch.

DGRP Freeze2.0 - SNPs			
**Variant**	**Allele DGN**	**Reference allele DGRP Freeze2.0**	**Alternate allele DGRP Freeze2.0**
p.Arg275Ser	Reference	130	0
p.Arg275Ser	Alternate	0	16
p.Tyr266His	Reference	111	0
p.Tyr266His	Alternate	0	40
p.Arg262Gly	Reference	133	0
p.Arg262Gly	Alternate	0	18
p.Ser244Gly	Reference	140	0
p.Ser244Gly	Alternate	0	12
p.Cys238Gly	Reference	143	0
p.Cys238Gly	Alternate	0	6
p.Asp228Glu	Reference	144	0
p.Asp228Glu	Alternate	0	7
p.Glu208Glu	Reference	150	0
p.Glu208Glu	Alternate	0	0
p.Tyr140Tyr	Reference	14	0
p.Tyr140Tyr	Alternate	0	136
p.Asp84Val	Reference	151	0
p.Asp84Val	Alternate	0	0
p.Leu81*	Reference	142	0
p.Leu81*	Alternate	0	9
p.Val76Ile	Reference	141	0
p.Val76Ile	Alternate	0	10
p.Thr67Ala	Reference	9	0
p.Thr67Ala	Alternate	0	143
p.Ser40Pro	Reference	7	0
p.Ser40Pro	Alternate	0	144
p.Val6Val	Reference	151	0
p.Val6Val	Alternate	0	1
c.-49G>T	Reference	148	0
c.-49G>T	Alternate	0	3
c.-153A>C	Reference	102	0
c.-153A>C	Alternate	0	18
c.-161T>A	Reference	124	0
c.-161T>A	Alternate	0	16
c.-212A>T	Reference	13	0
c.-212A>T	Alternate	0	132

c.-215G>A	Reference	13	0
c.-215G>A	Alternate	0	132
c.-234T>A	Reference	102	0
c.-234T>A	Alternate	0	34
c.-243A>T	Reference	11	0
c.-243A>T	Alternate	0	133
c.-323G>T	Reference	148	0
c.-323G>T	Alternate	0	3
c.-342C>T	Reference	121	0
c.-342C>T	Alternate	0	24
c.-366T>C	Reference	149	0
c.-366T>C	Alternate	0	3
**DGRP Sanger - Indels**			
**Upstream Indel**	**Allele DGN**	**Ancestral allele Sanger**	**Derived allele Sanger**
c.-439_-433del	Insertion	101	0
c.-439_-433del	Deletion	0	22
c.-334_-333insACATTCAT	Deletion	27	0
c.-334_-333insACATTCAT	Insertion	0	97
c.-171_-151del	Insertion	100	0
c.-171_-151del	Deletion	0	10
**GDL - SNPs**			
**Variant**	**Allele DGN**	**Reference allele GDL**	**Alternate allele GDL**
c.*67T>A	Reference	7	0
c.*67T>A	Alternate	0	49
c.*66C>T	Reference	52	0
c.*66C>T	Alternate	0	4
c.*44G>T	Reference	6	0
c.*44G>T	Alternate	0	49
c.*40T>C	Reference	50	0
c.*40T>C	Alternate	0	6
p.Arg275Ser	Reference	48	0
p.Arg275Ser	Alternate	0	8
p.Tyr266His	Reference	37	0
p.Tyr266His	Alternate	0	18
p.Arg262Gly	Reference	45	0
p.Arg262Gly	Alternate	0	10
p.Gln254*	Reference	50	0

p.Gln254*	Alternate	0	5
p.Ser244Gly	Reference	46	0
p.Ser244Gly	Alternate	0	8
p.Cys238Gly	Reference	49	0
p.Cys238Gly	Alternate	0	6
p.Asp228Glu	Reference	49	0
p.Asp228Glu	Alternate	0	6
p.Glu208Glu	Reference	49	0
p.Glu208Glu	Alternate	0	7
p.Glu202*	Reference	53	0
p.Glu202*	Alternate	0	3
p.Leu158Val	Reference	50	0
p.Leu158Val	Alternate	0	6
p.Val155Ile	Reference	50	0
p.Val155Ile	Alternate	0	6
p.Asn153Ile	Reference	50	0
p.Asn153Ile	Alternate	0	6
p.Ile152Ile	Reference	50	0
p.Ile152Ile	Alternate	0	6
p.Met141Lys	Reference	50	0
p.Met141Lys	Alternate	0	6
p.Tyr140Tyr	Reference	13	0
p.Tyr140Tyr	Alternate	0	43
p.Lys123Asn	Reference	53	0
p.Lys123Asn	Alternate	0	2
p.Met109Ile	Reference	50	0
p.Met109Ile	Alternate	0	6
p.Arg104Gln	Reference	50	0
p.Arg104Gln	Alternate	0	6
p.Lys89Arg	Reference	56	0
p.Lys89Arg	Alternate	0	0
p.Asp84Val	Reference	55	0
p.Asp84Val	Alternate	0	1
p.Leu81*	Reference	56	0
p.Leu81*	Alternate	0	0
p.Val76Ile	Reference	43	0
p.Val76Ile	Alternate	0	13
p.Thr67Ala	Reference	7	0
p.Thr67Ala	Alternate	0	49
p.Ser40Pro	Reference	4	0
p.Ser40Pro	Alternate	0	51
p.Val6Val	Reference	56	0

p.Val6Val	Alternate	0	0
**SD_SP Sanger - Indels + SNPs**			
**Variant**	**Allele DGN**	**Ancestral allele SD_SP Sanger**	**Derived allele SD_SP Sanger**
c.-439_-433del	Insertion	11	0
c.-439_-433del	Deletion	0	3
c.-334_-333insACATTCAT	Deletion	13	0
c.-334_-333insACATTCAT	Insertion	0	1
c.-171_-151del	Insertion	14	0
c.-171_-151del	Deletion	0	0
p.Leu81*	Wild-type	14	0
p.Leu81*	Stop gained	0	0
p.Glu202*	Wild-type	13	0
p.Glu202*	Stop gained	0	1
p.Gln254*	Wild-type	4	0
p.Gln254*	Stop gained	0	7
p.Phe217_Glu273del*	Insertion	11	0
p.Phe217_Glu273del*	Deletion	0	3

SI References

1. T. F. Mackay *et al.*, The Drosophila melanogaster Genetic Reference Panel. *Nature* **482**, 173-178 (2012).
2. W. Huang *et al.*, Natural variation in genome architecture among 205 Drosophila melanogaster Genetic Reference Panel lines. *Genome Research* **24** (2014).
3. A. R. Kraaijeveld, H. C. J. Godfray, Geographic Patterns in the Evolution of Resistance and Virulence in <i>Drosophila</i> and Its Parasitoids. *The American Naturalist* **153** (1999).
4. S. Dupas, M. Brehelin, F. Frey, Y. Carton, Immune suppressive virus-like particles in a <i>Drosophila</i> parasitoid: significance of their intraspecific morphological variations. *Parasitology* **113** (1996).
5. A. B. Leitão *et al.*, Independent effects on cellular and humoral immune responses underlie genotype-by-genotype interactions between Drosophila and parasitoids. *PLoS Pathogens* **15**, 1-13 (2019).
6. D. Bates, M. Mächler, B. Bolker, S. Walker, Fitting Linear Mixed-Effects Models Using lme4. *Journal of Statistical Software* **67** (2015).
7. T. Hothorn, F. Bretz, P. Westfall, Simultaneous Inference in General Parametric Models. *Biometrical Journal* **50** (2008).
8. M. J. Wade, R. G. Winther, A. F. Agrawal, C. J. Goodnight, Alternative definitions of epistasis: dependence and interaction. *Trends in Ecology & Evolution* **16** (2001).

9. J. Thurmond *et al.*, FlyBase 2.0: the next generation. *Nucleic Acids Research* **47**, D759-D765 (2019).
10. K. W. Broman, H. Wu, S. Sen, G. A. Churchill, R/qtl: QTL mapping in experimental crosses. *Bioinformatics* **19**, 889–890-889–890 (2003).
11. J. Varaldi, D. Lepetit, Deciphering the behaviour manipulation imposed by a virus on its parasitoid host: insights from a dual transcriptomic approach. *Parasitology* **145** (2018).
12. S. J. Gratz *et al.*, Highly Specific and Efficient CRISPR/Cas9-Catalyzed Homology-Directed Repair in <i>Drosophila</i>. *Genetics* **196** (2014).
13. J. Bischof, R. K. Maeda, M. Hediger, F. Karch, K. Basler, An optimized transgenesis system for Drosophila using germ-line-specific phiC31 integrases. *Proc Natl Acad Sci U S A* **104**, 3312-3317 (2007).
14. D. Korona *et al.*, Characterisation of protein isoforms encoded by the Drosophila Glycogen Synthase Kinase 3 gene shaggy. *PLoS One* **15**, e0236679 (2020).
15. T. Tokusumi, D. A. Shoue, Y. Tokusumi, J. R. Stoller, R. A. Schulz, New hemocyte-specific enhancer-reporter transgenes for the analysis of hematopoiesis in Drosophila. *Genesis* **47**, 771-774 (2009).
16. J. Rynes *et al.*, Activating transcription factor 3 regulates immune and metabolic homeostasis. *Mol Cell Biol* **32**, 3949-3962 (2012).
17. Z. Zhang, S. Schwartz, L. Wagner, W. Miller, A Greedy Algorithm for Aligning DNA Sequences. *Journal of Computational Biology* **7** (2000).
18. R. C. Edgar, MUSCLE: multiple sequence alignment with high accuracy and high throughput. *Nucleic Acids Res* **32**, 1792-1797 (2004).
19. M. A. Hanson *et al.*, The Drosophila Baramicin polypeptide gene protects against fungal infection. *PLoS Pathog* **17**, e1009846 (2021).
20. R. Yan, S. Small, C. Desplan, C. R. Dearolf, J. E. Darnell, Jr., Identification of a Stat gene that functions in Drosophila development. *Cell* **84**, 421-430 (1996).
21. L. J. Ko, J. D. Engel, DNA-binding specificities of the GATA transcription factor family. *Mol Cell Biol* **13**, 4011-4022 (1993).
22. H. Li, Aligning sequence reads , clone sequences and assembly contigs with BWA-MEM. **00**, 1-3 (2013).
23. T. Picard, No Title. *Broad Institute, GitHub repository* (2019).
24. H. Li, R. Durbin, Fast and accurate short read alignment with Burrows-Wheeler transform. *Bioinformatics* **25**, 1754-1760 (2009).
25. A. McKenna *et al.*, The Genome Analysis Toolkit: A MapReduce framework for analyzing next-generation DNA sequencing data. *Genome Research* **20** (2010).
26. L. J. Everett *et al.*, Gene expression networks in the Drosophila Genetic Reference Panel. *Genome Res* **30**, 485-496 (2020).
27. B. Longdon, J. D. Hadfield, C. L. Webster, D. J. Obbard, F. M. Jiggins, Host Phylogeny Determines Viral Persistence and Replication in Novel Hosts. *PLoS Pathogens* **7** (2011).
28. J. B. Lack, J. D. Lange, A. D. Tang, R. B. Corbett-Detig, J. E. Pool, A Thousand Fly Genomes: An Expanded <i>Drosophila</i> Genome Nexus. *Molecular Biology and Evolution* **33** (2016).
29. A. M. Bolger, M. Lohse, B. Usadel, Trimmomatic: a flexible trimmer for Illumina sequence data. *Bioinformatics* **30** (2014).
30. R. A. Hoskins *et al.*, Sequence Finishing and Mapping of Drosophila melanogaster Heterochromatin. *Science* **316** (2007).
31. V. Bansal, A statistical method for the detection of variants from next-generation resequencing of DNA pools. *Bioinformatics* **26** (2010).

32. P. Cingolani *et al.*, A program for annotating and predicting the effects of single nucleotide polymorphisms, SnpEff. *Fly* **6** (2012).
33. K. Ye, M. H. Schulz, Q. Long, R. Apweiler, Z. Ning, Pindel: a pattern growth approach to detect break points of large deletions and medium sized insertions from paired-end short reads. *Bioinformatics* **25** (2009).
34. J. T. Robinson *et al.*, Integrative genomics viewer. *Nature Biotechnology* **29** (2011).
35. D. Lüdecke, sjmisc: Data and Variable Transformation Functions. *Journal of Open Source Software* **3** (2018).
36. J. K. Grenier *et al.*, Global Diversity Lines–A Five-Continent Reference Panel of Sequenced <i>Drosophila melanogaster</i> Strains. *G3: Genes|Genomes|Genetics* **5** (2015).
37. F. Madeira *et al.*, The EMBL-EBI search and sequence analysis tools APIs in 2019. *Nucleic Acids Research* **47** (2019).
38. P. Danecek *et al.*, The variant call format and VCFtools. *Bioinformatics* **27** (2011).
39. J. Parsch, S. Novozhilov, S. S. Saminadin-Peter, K. M. Wong, P. Andolfatto, On the utility of short intron sequences as a reference for the detection of positive and negative selection in Drosophila. *Mol Biol Evol* **27**, 1226-1234 (2010).
40. T. Jombart, adegenet: a R package for the multivariate analysis of genetic markers. *Bioinformatics* **24** (2008).
41. J. Goudet, hierfstat, a package for r to compute and test hierarchical F-statistics. *Molecular Ecology Notes* **5** (2005).
42. V. Obenchain *et al.*, VariantAnnotation: a Bioconductor package for exploration and annotation of genetic variants. *Bioinformatics* **30** (2014).
43. H. Li *et al.*, The Sequence Alignment/Map format and SAMtools. *Bioinformatics* **25** (2009).
44. S. Kumar, G. Stecher, M. Li, C. Knyaz, K. Tamura, MEGA X: Molecular Evolutionary Genetics Analysis across Computing Platforms. *Molecular Biology and Evolution* **35** (2018).
45. K. P. Schliep, phangorn: phylogenetic analysis in R. *Bioinformatics* **27** (2011).
46. E. Paradis, J. Claude, K. Strimmer, APE: Analyses of Phylogenetics and Evolution in R language. *Bioinformatics* **20** (2004).
47. X. Yi *et al.*, Sequencing of 50 Human Exomes Reveals Adaptation to High Altitude. *Science* **329** (2010).
48. J. Y. Cheng, A. J. Stern, F. Racimo, R. Nielsen, Detecting Selection in Multiple Populations by Modeling Ancestral Admixture Components. *Mol Biol Evol* **39** (2022).
49. C. C. Chang *et al.*, Second-generation PLINK: rising to the challenge of larger and richer datasets. *Gigascience* **4**, 7 (2015).
